# Elimination of substances from the brain parenchyma: efflux via perivascular pathways and via the blood–brain barrier

**DOI:** 10.1186/s12987-018-0113-6

**Published:** 2018-10-19

**Authors:** Stephen B. Hladky, Margery A. Barrand

**Affiliations:** Department of Pharmacology, University of Cambridge, Cambridge, CB2 1PD UK

**Keywords:** ABC transporters, Amino acid metabolism, Basement membrane, Blood–brain barrier permeability, Branched chain amino acid shuttle, Carrier mechanism, Diffusion, Efflux rate constant, Linear free energy relations, Perivascular convection, SLC transporters, Transcytosis, Trans-stimulation, Volume of distribution

## Abstract

This review considers efflux of substances from brain parenchyma quantified as values of clearances (*CL*, stated in µL g^−1^ min^−1^). Total clearance of a substance is the sum of clearance values for all available routes including perivascular pathways and the blood–brain barrier. Perivascular efflux contributes to the clearance of all water-soluble substances. Substances leaving via the perivascular routes may enter cerebrospinal fluid (CSF) or lymph. These routes are also involved in entry to the parenchyma from CSF. However, evidence demonstrating net fluid flow inwards along arteries and then outwards along veins (the glymphatic hypothesis) is still lacking. *CL*_perivascular_, that via perivascular routes, has been measured by following the fate of exogenously applied labelled tracer amounts of sucrose, inulin or serum albumin, which are not metabolized or eliminated across the blood–brain barrier. With these substances values of total *CL* ≅ 1 have been measured. Substances that are eliminated at least partly by other routes, i.e. across the blood–brain barrier, have higher total *CL* values. Substances crossing the blood–brain barrier may do so by passive, non-specific means with *CL*_blood-brain barrier_ values ranging from < 0.01 for inulin to > 1000 for water and CO_2_. *CL*_blood-brain barrier_ values for many small solutes are predictable from their oil/water partition and molecular weight. Transporters specific for glucose, lactate and many polar substrates facilitate efflux across the blood–brain barrier producing *CL*_blood-brain barrier_ values > 50. The principal route for movement of Na^+^ and Cl^−^ ions across the blood–brain barrier is probably paracellular through tight junctions between the brain endothelial cells producing *CL*_blood-brain barrier_ values ~ 1. There are large fluxes of amino acids into and out of the brain across the blood–brain barrier but only small net fluxes have been observed suggesting substantial reuse of essential amino acids and α-ketoacids within the brain. Amyloid-β efflux, which is measurably faster than efflux of inulin, is primarily across the blood–brain barrier. Amyloid-β also leaves the brain parenchyma via perivascular efflux and this may be important as the route by which amyloid-β reaches arterial walls resulting in cerebral amyloid angiopathy.

**Electronic supplementary material:**

The online version of this article (10.1186/s12987-018-0113-6) contains supplementary material, which is available to authorized users.

## Background

Maintaining the status quo of the cellular environment in the brain is essential for correct functioning of neurons. Thus the brain is protected by being separated from the rest of the body by a set of barriers. These barriers hinder entry of unwanted substances from the circulation but at the same time provide for the removal of potentially toxic substances that have inadvertently entered or been produced within the brain. These barriers will of course present challenges for delivery of nutrients, essential for normal brain growth, metabolism and function.

**Fig. 1:**
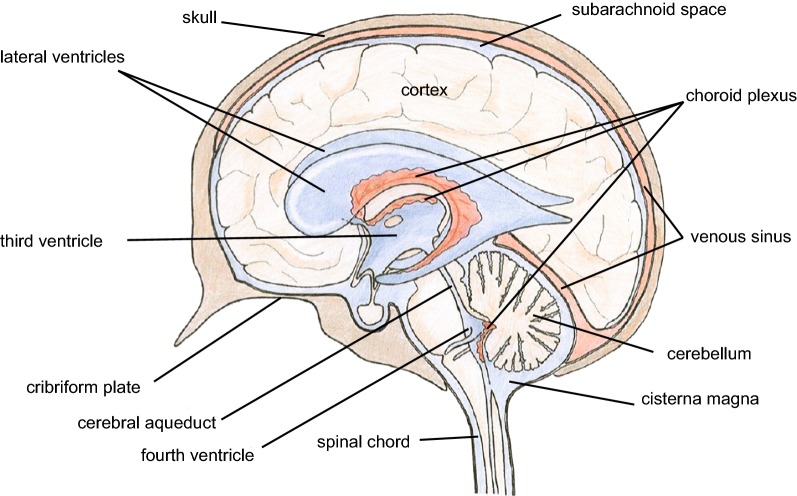
Mid-saggital section of the brain showing locations of the ventricles, cerebral aqueduct, subarachnoid spaces (including the basal cisterns) and choroid plexuses. The choroid plexuses are discrete epithelial structures located in the cerebral ventricles that secrete cerebrospinal fluid (CSF) shown in pale blue, which fills the ventricles and subarachnoid spaces. Normally there is net flow of CSF from the ventricles into the cisterna magna and from there to the other subarachnoid spaces of the brain and spinal cord. Reproduced but relabelled with permission from Strazielle et al. [20]

**Fig. 2:**
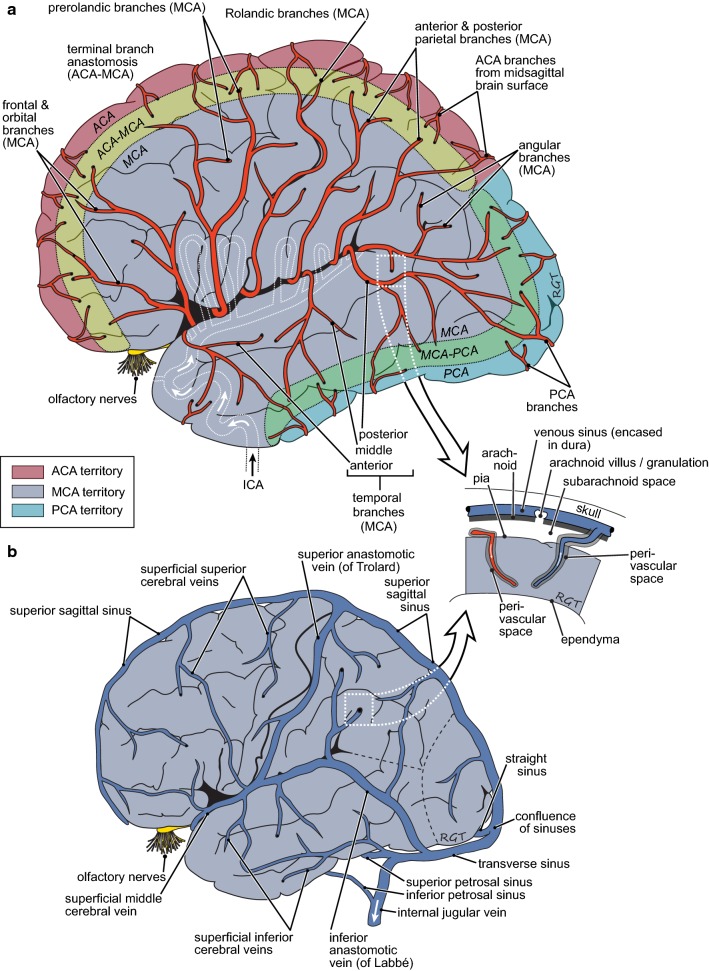
Schematic diagrams of the lateral surface of the brain showing **a** the arterial supply and **b** the venous drainage with an inset indicating the relations of the pia, the ependyma and the perivascular spaces to the brain parenchyma. The large vessels run parallel to the surfaces of the brain, with smaller branches that penetrate into the parenchyma more or less perpendicular to the surfaces (see inset). Points of penetration of the vessels down into the parenchyma are indicated by black dots at the end of vessels. Branching of arteries continues within the parenchyma yielding arterioles and eventually capillaries that then join forming venules and then veins. These merge and drain into the large veins and venous sinuses on the surface. As discussed in the text blood vessels within the parenchyma have associated perivascular spaces that provide preferential routes for materials to enter and leave the parenchyma. Figure drawn by Robert G. Thorne and used with permission. See [639] for a succinct but still thorough description of human anatomy relevant to delivery of substances to the brain and their removal from it

The brain is effectively a greatly distorted blind-ended tube. The four ventricles (see Fig. 1) form the inside of the tube and the brain parenchyma, comprised of brain cells and the interstitial spaces between them, makes up the wall. The tube is surrounded by the subarachnoid spaces, which in this discussion are taken to include the basal cisterns. Both ventricles and subarachnoid spaces are filled with cerebrospinal fluid (CSF). The inside of the tube at the IVth ventricle is connected to the outside of the tube at the cisterna magna via the foramina of Magendie and Luschka. The subarachnoid spaces are bounded on their outside by the outer meninges composed of the arachnoid and the dura (see Fig. 2 inset), which are in turn encased by the skull (see [1]). On their inside the subarachnoid spaces are separated from the brain parenchyma by a cell layer, the pia mater or inner meninges, and one or more layers of astrocyte endfeet, the glia limitans. The surfaces of the parenchyma adjacent to the ventricles are covered by a layer of cells, the ependyma (see Fig. 2 inset).

Current evidence indicates that most of the CSF is secreted into the ventricles by the choroid plexuses (see Fig. 1 and for reviews [2–4]). While there are to and fro movements of CSF driven by the cardiac and respiratory cycles [5–7] and considerable convective mixing of CSF within the ventricles [8, 9], net flow is normally from the choroid plexuses in the ventricles towards the cisterna magna and onwards via the subarachnoid spaces to the various sites of CSF outflow. Most but not all studies show that in the absence of hydrocephalus there is transfer of solutes and fluid through the cerebral aqueduct connecting the IIIrd to the IVth ventricle but only limited transfers from the IVth to the IIIrd ventricle [9–16].^1^

The cells of the ependymal layer bordering the ventricles are not bound together by tight junctions and the layer is thought to be permeable to small solutes and proteins [17–20]. However, diffusion in the parenchyma is too slow to transfer material more than several hundred microns within 1–2 h^2^ (see e.g. [17, 21–25]). Thus normally neither transfer across the ependyma nor flow of CSF provides a rapid route for substances to reach the choroid plexuses from most of the parenchyma. For this reason, other than as the primary source of CSF, the choroid plexuses do not feature prominently in this review, which is concerned primarily with elimination of substances from the parenchyma.^3^ Readers interested in transporters at the choroid plexuses and the transport they mediate are well served by other reviews [2–4, 20, 26–38].

The brain parenchyma is extensively vascularized (see Fig. 2). Blood arrives in large arteries which course over the outer surfaces of the brain before diving into the parenchyma. Similarly blood leaves the parenchyma in veins and venous sinuses also located at the outer surfaces. Within the parenchyma the arterial vessels branch out leading eventually to microvessels which then join together to form veins. There are so many microvessels that at least one is within a few tens of microns of every parenchymal cell. The endothelial cells lining the microvessels in the brain provide the blood–brain barrier, the most important route for exchange of materials between blood and parenchyma. Three important characteristics of the barrier are: the microvessels are close to each other so that diffusion distances are short; the surface area of the barrier is enormous, and the barrier is permeable to those substances required to move readily in or out of the brain.

In addition to the blood–brain barrier there are perivascular spaces that can provide conduits for substances to move into and out of the brain parenchyma. (“Perivascular” is used here to describe various possible routes available along the walls of blood vessels but separated from the blood flowing through the vascular lumen (see “Nomenclature”, p. 59 in [4] and similar usage in [16, 39, 40]). As indicated schematically in the inset of Fig. 2, these spaces are to be found around the arteries entering and the veins leaving the parenchyma (see Sect. 3.1). They provide routes for movement of substances between parenchyma and the CSF in the subarachnoid spaces or possibly directly to lymph. As discussed in Sect. 3, such movement is much faster than could be supported by diffusion alone. By contrast movement of substances between CSF and parenchyma across the pia/glial layers and ependyma is limited by diffusion in the parenchyma (in the absence of imposed osmotic gradients or infusions of fluid) and, except for regions of parenchyma very close to the surfaces (or to some extent in white matter, see Sect. 3.1), is much slower than movement via the perivascular spaces. Hence the major routes for efflux of substances from the brain parenchyma are transfer across the blood–brain barrier and movements towards the outer surfaces of the brain via the perivascular spaces.

The blood–brain barrier provides a route for efflux of solutes that are sufficiently small and lipid soluble (see Sect. 4.1) and it also contains specific transporters that can transfer many polar substances. The perivascular route is especially important for the elimination of large or polar solutes for which there are no specific transporters (see Sect. 3).

**Fig. 3:**
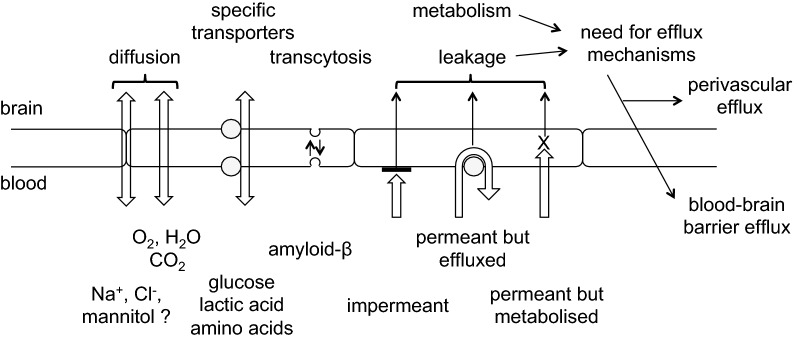
Mechanisms for transfers into and out of the brain across the blood–brain barrier and the need for efflux mechanisms. Passive, non-specific transfers can occur via paracellular and transcellular routes, though the rates for paracellular transfer are small. Specific transcellular transport can be passive or active. In addition to the transfers for well-known substances many others are able to enter at various rates, either because they are sufficiently small and lipid-soluble or because barrier mechanisms are not perfect. Substances which enter even though they shouldn’t or are produced “accidentally” by metabolism cannot be allowed to accumulate within the brain. Thus there must be mechanisms for eliminating them

The types of mechanisms present at the blood–brain barrier that allow easy passage of nutrients like glucose and amino acids and wastes like CO_2_ are shown in Fig. 3 along with indication of the need for expulsion of substances that should not be allowed to enter or accumulate in the brain. Because the gaps between the endothelial cells are occluded by tight junctions that greatly reduce the paracellular passage of solutes even as small as sugars and inorganic ions like Na^+^, K^+^ and Cl^−^,^4^ to enter or leave the brain across the blood–brain barrier almost all substances must pass through the cells, which means they must cross both the luminal and abluminal membranes.

Polar substances like sugars, amino-acids, and many foreign molecules can cross the blood–brain barrier rapidly only if there are specific mechanisms provided (see Sect. 4). Indeed the blood–brain barrier has very low permeability to those polar substances that are unable to be carried by specific transporters. By contrast lipid soluble substances that are small (MW < ~ 600) and so able to cross cell membranes unaided are more likely to be able to cross the blood–brain barrier into the brain. However even some of these are denied entry by specific efflux mechanisms that transport them back to blood from the endothelial cells, e.g. by ABC efflux transporters, notably *p*-glycoprotein (Pgp), and breast-cancer resistance protein (BCRP), or by metabolism within the cells, e.g. by monoamine oxidase (MAO).

**Fig. 4:**
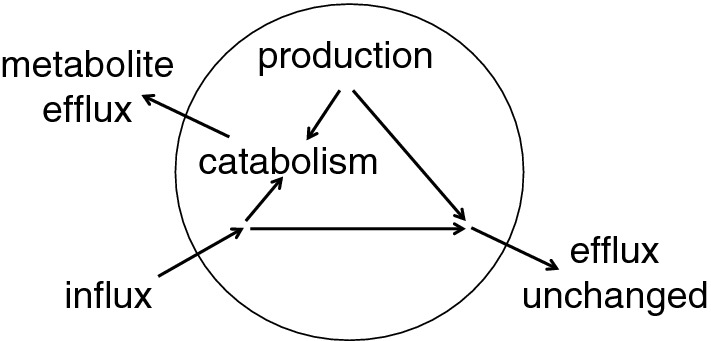
Elimination of unwanted substances can be either by efflux alone or it can be by metabolism followed by efflux of the metabolites

Much is known and has been written about how substances enter the brain, about how others are prevented from doing this, and about the importance of the blood–brain barrier for delivery of drugs to the brain. Reviews include those dealing with glucose, water, and inorganic ions [2–4, 41]; those considering amino acids [4, 42–44]; and those concerned with a wide variety of other substances [20, 30, 32, 36, 38, 45–51]. However, much less has been investigated and/or written about how substances are eliminated from the brain. As indicated in Fig. 3 though there are numerous mechanisms for reducing entry of unwanted substances, it is equally important to have some means of expelling unwanted substances including those that have gained entry and those that have been formed within the brain (see Fig. 4). The rate of elimination is important for all substances that can enter and leave the brain because it determines the concentrations that can be achieved for any rate of entry. In the case of administered drugs, the rate of elimination also determines how long concentrations will persist between or after doses.

Elimination thus plays a key role in maintenance of the status quo in the brain. The principles involved in balancing inputs and outputs and what is meant by “clearance” are both considered more fully in Sect. 6. The relationship between rates of elimination, clearances, permeability-area products, volumes of distribution and half-lives together with the units used are described in Appendix A. The routes of elimination and the mechanisms by which elimination is brought about are the main subjects of this review.

## Removal of substances from the brain parenchyma: overview

There are three possible pathways by which substances can be removed from the brain parenchyma: via transport to blood across the blood–brain barrier; via exit to CSF or possibly directly to lymph followed by subsequent transfer to blood; or via metabolism to different substances. The relative importance of each of these pathways as a mechanism of removal depends on the nature of the substance under consideration.

In the case of metabolism, though the original substance is removed, the resulting metabolites still eventually require elimination as well. Glucose for instance is largely removed by metabolism to CO_2_ and water but these species must then exit the brain. At the opposite extreme inorganic ions such as Na^+^ and K^+^ cannot be metabolized and are removed by efflux in their original forms.

Convection of fluid along perivascular spaces facilitates efflux (as well as influx) of a range of large polar substances such as serum albumin, inulin, sucrose, and various dextrans and polyethylene glycols. Efflux of these substances from parenchyma to CSF (or lymph) via the perivascular spaces is relatively slow, taking hours, but it is still much more rapid than could be supported by diffusion over the large distances involved suggesting that it is occurring by some sort of flow (see Sect. 3.2). The exact ways in which perivascular influx and efflux of solutes and water take place have been controversial as considered in some detail in Sect. 3. Tarasoff-Conway et al. [52] have addressed the issue of perivascular clearance with particular regard to one particular solute, amyloid-β. Brinker et al. [53], Hladky and Barrand [41], Simon and Iliff [39], Coles et al. [1], Abbott et al. [40], and Benveniste et al. [54] have summarized the evidence concerning perivascular transport from various perspectives.

Transport across the blood–brain barrier is the dominant mechanism for removal of water and CO_2_ from brain parenchyma (for discussion and references see [4]). Molecules less lipid soluble or somewhat larger than H_2_O need specific transporters in the endothelial cell membranes of the barrier, e.g. for glucose GLUT1, which is found in both luminal and abluminal membranes. Transporters are present for a large number of substances [20, 31, 46, 55–58] (see Sect. 4.2). Certain larger solutes, e.g. insulin [59], transferrin [60, 61] and β-amyloid [62], may be transported across the blood–brain barrier by transcytosis [36, 63, 64] (see Sect. 4.3).

Many of the transporters found at the blood–brain barrier are capable of mediating not only efflux but also influx and have been studied more thoroughly from this standpoint. Other transporters, e.g. the ABC efflux pumps that are present in the luminal membranes of the endothelial cells (see Sect. 4.2.1), transfer many exogenous substances in an outward direction from endothelial cells to blood fuelled by the energy derived from ATP hydrolysis. This outward movement serves to decrease blood-to-brain influx as substances that enter the endothelial cells (or even just the luminal membranes of the cells) are returned to blood before they enter the brain proper. ABC transporters may also promote brain-to-blood efflux if there is some means for the substances to enter the endothelial cells across the abluminal membranes (see Sects. 4.2.1 and 4.2.2).

## Perivascular pathways

### Routes of perivascular efflux

**Fig. 5:**
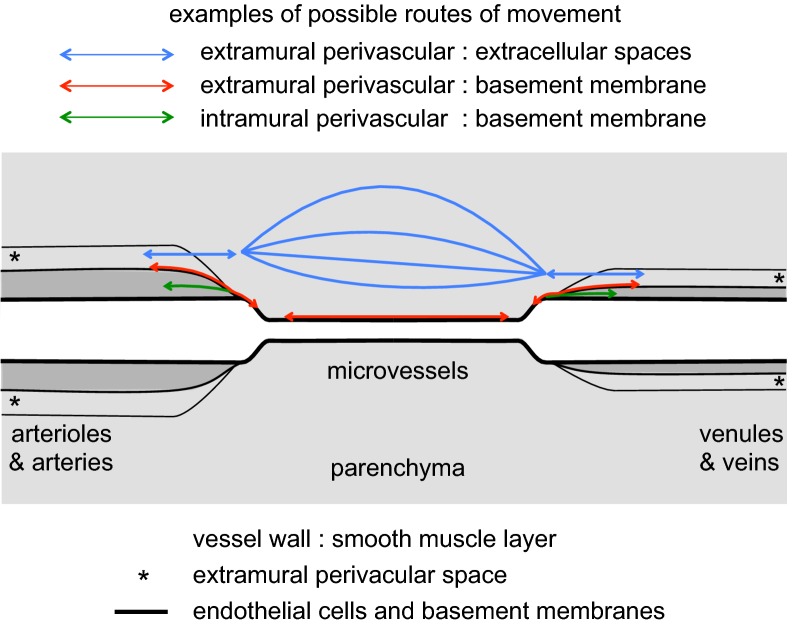
Diagram indicating putative perivascular routes for substances to move into, out of and through the brain parenchyma. The lumens of arteries, arterioles, venules and veins are surrounded by a layer of endothelial cells with a basement membrane, then a layer of vessel wall including smooth muscle, and outside that there may be a further perivascular space with fluid and connective tissue bounded by basement membranes of the smooth muscle, pial and glial cells. Close to the surfaces of the brain these further spaces are often called Virchow-Robin spaces. Movements parallel to the large vessels may be intramural, through the extracellular space of the vessel wall, or extramural either in the outermost basement membranes or, in the opinion of some workers, in a fluid filled space. In this review both intramural and extramural pathways are called perivascular routes. Parallel to microvessels movement may be preferentially within the basement membrane separating the endothelial cells from the glial endfeet or it may be more diffuse through the interstitial spaces between the parenchymal cells

Some of the possible routes for perivascular movements of solutes are indicated in Fig. 5. Whether or not actual fluid filled spaces exist around the blood vessels, it is believed that substances can move along preferential routes parallel to the blood vessels. (The description that follows is primarily for grey matter. As suggested originally by Rosenberg et al. in 1980 [65] there are likely to be preferential routes for fluid movement parallel to axons in white matter. It should also be noted that there may be regional variations, see e.g. [66, 67]). The idea that the basement membranes of microvessels can provide a preferential route stems from observations that when horseradish peroxidase is introduced into CSF with consequential influx along arteries the peroxidase is found to be localized in the basement membranes around microvessels. The idea has subsequently been supported by similar observations for other macromolecules (see e.g. [16, 68–72]). However, calculations by Asgari et al. [73] imply that unless the matrix of the microvascular basement membranes has a resistance substantially less than a sleeve of ^®^Matrigel with the same dimensions, they will not provide a preferential route for fluid flow parallel to the microvessels. A preferential route for movement along the vessels does not conflict with the movements of solutes outward by diffusion into the surrounding interstitial fluid. Regardless of whether or not the microvessel basement membranes provide a route with relatively low resistance, the distance from anywhere in the parenchyma to the nearest larger vessel is still likely to be relatively small, e.g. 100–200 µm. (Striking images of the vascular tree can be seen in [72]). For distances this short, diffusion is expected to be the dominant mechanism of extracellular movement [16, 24, 72, 74–81].

Markers for perivascular transport clearly have perivascular pathways for entry and exit from the parenchyma, but there is controversy as to whether efflux, influx or both occur along arteries and/or veins (for discussion see [16, 39, 41, 52, 72]). Efflux along arteries has been seen in many studies (e.g. [70, 82–88]) with substances even reaching the large arteries near the circle of Willis [82], and influx has also been seen in many studies [15, 16, 25, 69, 71, 79, 84, 88–92]. Evidence of influx along some vessels was obtained as early as 1960 [93]. Perivenous influx [16] and efflux [25, 69, 84, 94] have been reported. Efflux along unspecified blood vessels has also been seen [79]. The available evidence suggests that both influx and efflux occur along both arteries and veins [41, 78, 95] either via common pathways or separately along parallel pathways [88, 95] (see Proposal 2 below). In Fig. 5 movements are shown as occurring in both directions along both.

There has also been disagreement over which of the structural components of the arteries provide the principal routes for periarterial transport with some favouring an extramural, fluid filled perivascular space, possibly containing connective tissue fibres [16], between the vessel walls and the astrocyte endfeet, see e.g. [25, 71, 78, 79, 81, 83–85, 87, 92, 96]^5^ while others favour the view that “perivascular spaces” are not fluid filled, free spaces but rather perivascular pathways via basement membranes either within the smooth muscle layer or on the outside surface of the artery [52, 70, 72, 88, 97–99] (see Fig. 5).

Free spaces may be highly compressible, allowing modest changes in pressure to change their dimensions as envisaged in the proposal that variations in the blood pressure within the vessels somehow drive perivascular movements. By contrast basement membranes are likely to be much less compressible and are likely to offer much greater resistance to flow (see [73, 100, 101]), thus precluding blood pressure variations as the driving force for perivascular flow (see next section). Diem et al. [100] have proposed vasomotion as an alternative. Pizzo et al. [16] have suggested that both basement membrane routes and other, extramural routes exist with their relative importance depending on the size of vessel and the size of the solute. Another proposed variation is a hybrid with an extramural basement membrane route mediating fluxes into the brain and an intramural basement membrane route between smooth muscle cells mediating fluxes outwards [88, 95].

It is quite evident that solutes even as large as amyloid-β have access to the basement membranes between the smooth muscle cells (see e.g. [16, 70, 93, 102]), but it is not known whether the solutes reach these locations via an intramural route with movement along basement membranes as favoured by Carare, Weller, Hawkes and colleagues [70, 88, 95] or via extramural pathways with subsequent penetration from these into the basement membranes within the vessel wall (see Figure 21 in Sect. 5.7.1.2) or some mixture of the two. Arbel-Ornath et al. [87] used two-photon imaging to investigate the position of a 3 kDa fluorescent dextran during efflux following injection into the parenchyma. Shortly after injection they saw fluorescence within the parenchyma, in perivascular spaces surrounding small arteries and, at lower concentration, between the smooth muscle cells.

**Fig. 6:**
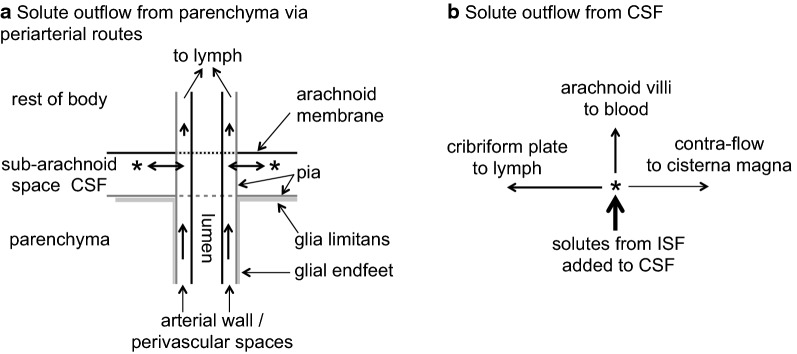
Schematic diagram indicating possible routes for efflux of large solutes from the parenchyma along perivascular routes. **a** Large solutes emerging from the parenchyma via intramural or extramural routes along arteries (and possibly veins) may either mix with CSF or continue along the walls of blood vessels. The blood vessels span the subarachnoid space (see Figs. 1 and 6) before leaving the brain to reach the rest of the body. The fluid that continues along these vessels may enter either blood or lymph, but solutes as large as serum albumin will enter only lymph. **b** Large solutes that have reached CSF will be taken to sites of CSF outflow including the arachnoid villi, where the solutes will enter venous blood, and the cribriform plate, where they will enter lymph. (Based primarily on data for radio-iodinated serum albumin RISA [82, 83, 125] and on the location of the pia surrounding arteries taken from [103]). The anatomical relations of the pathways or spaces remain controversial

There has been controversy about the nature of the connections between the perivascular spaces adjacent to larger blood vessels within the parenchyma, the CSF and the perivascular spaces of the vessels passing through the subarachnoid spaces [1, 16, 25, 54, 71, 72, 81, 103–109]. However, whatever the exact perivascular pathway used, solutes exiting from the parenchyma along perivascular routes appear to be effluxed partly to CSF in the basal cisterns or subarachnoid spaces and partly to the outer meninges [85] and/or lymphatics [94, 107, 109–115]. Movement of small solutes and water does take place between fluid in the subarachnoid space and fluid within the perivascular spaces (see Section 4.1.1.1 of [41]). However a substantial proportion of perivascular efflux of large solutes appears to pass to lymph without first appearing in CSF in the cisterna magna^6^ (see Fig. 6) [16, 39, 52, 82, 83, 94, 96, 105, 107, 111, 115–119].

Those solutes that do reach CSF from the parenchyma can be taken out of the cranium via CSF outflow. Routes for CSF outflow were reviewed comprehensively by Pollay in 2010 [119] This outflow is partly via arachnoid villi, partly via perineural routes including those across the cribriform plate to the nasal mucosa [119–121] and possibly also via extra-parenchymal perivascular routes (see Fig. 6) [16, 81, 105, 111, 119, 122–124]. Outflow via arachnoid villi leads directly to venous blood while outflow via the cribriform plate may deliver solutes directly to lymphatics or to the extracellular fluid in the nasal mucosa [118, 121, 125]. Small solutes (e.g. lactate) and solutes even as large as inulin may leave the nasal mucosa by entering blood across peripheral capillary walls but larger solutes (e.g. albumin) will leave via lymph flow to cervical lymph nodes [125]. Outflow via other routes leads at least in part to lymph (see e.g. [111]).

### Mechanisms driving perivascular solute efflux

**Fig. 7:**
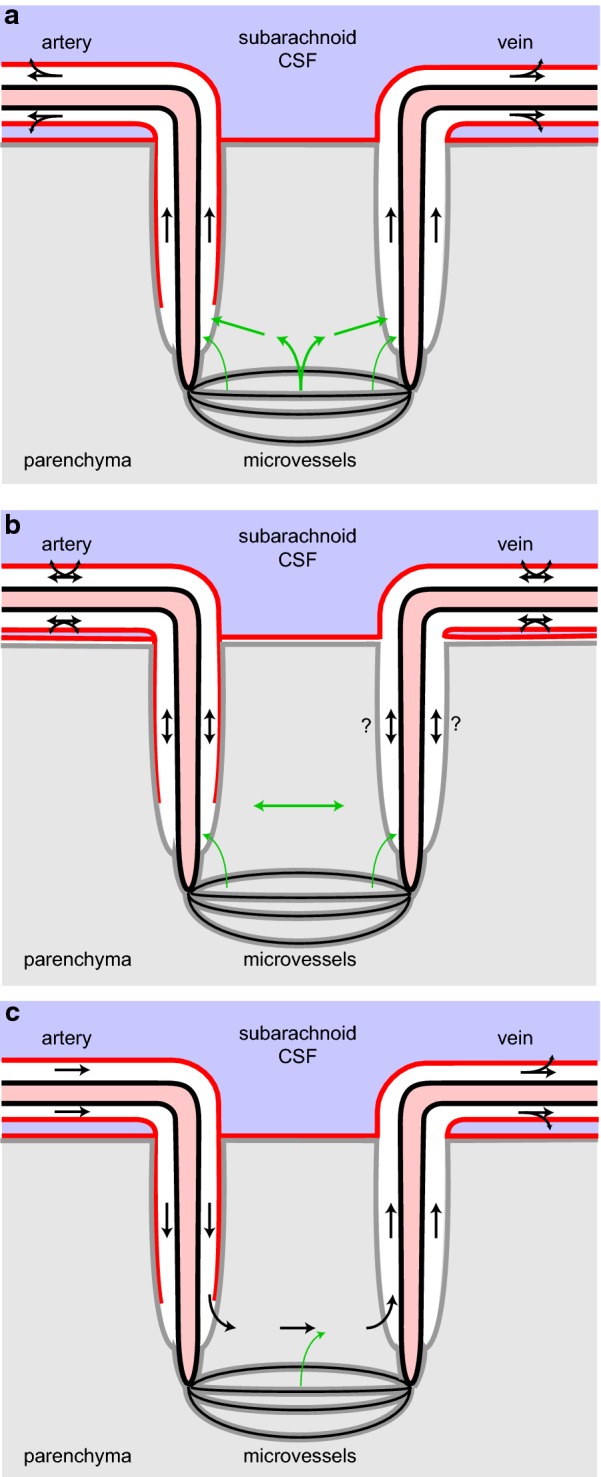
Proposals to explain rapid transfer of markers via periarterial spaces: **a** the original proposal; **b** proposed perivascular convection and interstitial diffusion **c** the glymphatic proposal. In **a** the blood–brain barrier secretes fluid which flows out of the parenchyma via preferred routes (here the perivascular routes). In **b** transport in the perivascular spaces is assisted by convective stirring or mixing. In **c** (see Figure 5 of Iliff et al. [25]) there is preferential inflow via the space between the arterial wall and the pial sheath and preferential outflow via spaces surrounding veins. Red lines represent pial membranes, grey lines the layer of glial end-feet or glia limitans, black arrows are fluxes of markers carried or assisted by convection, green arrows are primarily diffusion. The location of the pial barriers is based on Zhang et al. [23]. The anatomical basis of the perivascular spaces remains controversial (Modified from Figure 9 in [41])

Diffusion is not adequate for perivascular influx because substances added to CSF are found deep in the parenchyma much too quickly for diffusion over the distance involved, a millimeter or more [25, 68, 69, 84]. Similarly diffusion cannot account for efflux from parenchyma to CSF of substances like polyethylene glycol and dextran [126, 127], serum albumin [83], mannitol [25] or inulin [62, 128]. Thus alternative mechanisms have been proposed (see Fig. 7).

*Proposal 1* The first proposal (Fig. 7a) was that secretion of fluid by the blood–brain barrier provides a small pressure gradient for outflow of ISF along preferential routes (see [83, 126, 127, 129, 130]). These routes could be perivascular spaces or the extracellular spaces parallel to the axons in nerve fibre tracts. When this proposal was put forward more than 30 years ago (see e.g. [83]) it was believed that the half-life for clearance of marker solutes by outflow was of the order of 12 h. However, all of these early studies were performed on animals anaesthetized using barbiturates. Using either conscious animals or those anaesthetized with ketamine/zylazine or halothane, the half-lives are much shorter, 2–4 h [25, 62, 85, 131]. Perivascular efflux of solutes is considerably faster than envisaged by Cserr and coworkers. It should also be pointed out that Proposal 1 does not and was never intended to provide any explanation for the rapid influx of solutes. In Proposal 1 (and in Proposal 3, see below) the solutes are swept out of the parenchyma by the flow through the perivascular system. Estimates of the flow rate required to eliminate substances at the observed rates can be calculated from their clearances

1 $$CL_{perivascular} \,=\, {{{\text{rate}}\;{\text{of}}\;{\text{elimination}}} \mathord{\left/ {\vphantom {{{\text{rate}}\;{\text{of}}\;{\text{elimination}}} {c_{isf} }}} \right. \kern-0pt} {c_{isf} }}$$

and the assumption that the concentration of the solute is the same in ISF and the outflow. Then because elimination is by outflow

2 $${\text{rate}}\;{\text{of}}\;{\text{elimination}} = {\text{rate}}\;{\text{of}}\;{\text{outflow }} \times {\text{ concentration in outflow}}$$

and substituting that into the definition of clearance,

3 $$CL_{perivascular} = {{{\text{rate}}\;{\text{of}}\;{\text{outflow}}\; \times \;{\text{concentration in outflow}}} \mathord{\left/ {\vphantom {{{\text{rate}}\;{\text{of}}\;{\text{outflow}}\; \times \;{\text{concentration in outflow}}} {c_{isf} }}} \right. \kern-0pt} {c_{isf} }},$$

which, because the concentration in the outflow is the same as *c*_*isf*_, becomes

4 $$CL_{perivascular} = {\text{rate}}\;{\text{of}}\;{\text{outflow}} .$$

From the known volume of distribution of suitable substances such as inulin or sucrose, 200 µL g^−1^, and the range of their half lives, 2–4 h, and the relation between clearance, half-life and volume of distribution, *CL* = 0.69 *V*_*D*_/*t*_1/2_, the clearances and thus the required flow rates are in the range 0.6–1.2 µL g^−1^ min^−1^. For a human with a 1400 g brain this is 1.2–2.4 L day^−1^. Even the bottom of this range is somewhat more than twice the rate of production of CSF. There is no other reason to suspect that there is a rate of secretion of fluid across the blood–brain barrier that exceeds the rate of fluid secretion by the choroid plexuses (see Section 4.1 in [4]). The rate of fluid secretion across the blood–brain barrier is very unlikely to be this large and is almost certain to be insufficient to account for perivascular clearance of solutes.

*Proposal 2* (Fig. 7b) The second suggestion, recently revived, is that convection in the perivascular spaces, arterial and possibly venous, leads to convective mixing of the fluid in the spaces allowing relatively rapid movements of solutes both inwards and outwards [41, 78, 82, 96, 132]. Such mixing probably presupposes that perivascular spaces are compressible. Convective mixing is perhaps better called dispersion [78]. Papisov [133] and Asgari et al. [134] discuss a similar effect in the spinal cord allowing transport of solutes down their concentration gradients against the direction of net flow of CSF and at rates much greater than allowed by diffusion. In this proposal diffusion is taken to be adequate to explain movements within the interstitial spaces in the parenchyma because the distances involved are sufficiently short (see Sect. 3.2.1).

In this proposal (and in Proposal 3, see below), an important part of the mechanism is thought to be convection in spaces whose dimensions are changed by periodic compression resulting from the changes in blood pressure during the cardiac cycle [13, 25, 70, 82, 96, 132]. The length of space around a cortical vessel that is compressed at one time is as long as the vessel [78, 82]. Bradbury et al. [82] were of the opinion that periodic compression and reexpansion of this space “would cause to-and-fro movement of fluid in and out of the brain” such that “A basis would be provided for substances in solution or suspension to be moved either out of or into the brain depending on the relative concentration in subarachnoid CSF.” Another variation on this theme may be possible if there are layers of differing compressibility, both connected via relatively low resistance pathways to the brain surface.

Back-and-forth convective movements in perivascular spaces would only be apparent using techniques with both good spatial resolution and time resolution better than a fraction of a second. Such movements have been observed in perivascular spaces very close to the cortical surface using india ink [84] and in the periarterial spaces at the cortical surfaces using microspheres [108]. But with techniques now available for viewing, if perivascular spaces exist that allow convective back and forth movements, all that would be seen within the parenchyma would be accelerated movement down the concentration gradient regardless of its direction, i.e. the periarterial influxes and effluxes that have been observed.

*Proposal 3* (Fig. 7c) The third proposal, the glymphatic hypothesis [25, 109, 135–137], asserts.

- There is an inward flow of CSF along periarterial spaces;
- The flow is driven across the layer of astrocyte endfeet into the parenchyma aided by the presence of Aqp4 in the endfeet;
- The flow propels the waste products of metabolism into the perivenous space again crossing the layer of endfeet, presumably again aided by the presence of Aqp4;
- The flow exits the parenchyma by the perivenous route and reaches lymphatic vessels in the neck.

As indicated when considering Proposal 1, a flow of ~ 0.6 µL g^−1^ min^−1^ or more would be required to remove the efflux markers at the observed rate. For a 1400 g brain, that is c. 1.2 L day^−1^ roughly twice the generally accepted rate of CSF production. Thus even if the rest of this proposal is correct, either the glymphatic flow does not direct ISF out of the brain directly to lymphatic vessels or the rate of CSF production is greater than is generally accepted.

The earlier evidence for and against the glymphatic hypothesis was discussed in [41] where it was argued that while a recirculation of CSF could explain influx and efflux of substances much faster than by simple diffusion, it did not explain either the observed outward movements of solutes along arteries [70, 71, 82, 83, 87, 130] or the observed continuation of rapid inward periarterial movement of large solutes when the proposed glymphatic circulation was interrupted at the level of the astrocyte endfeet by global knockout of Aqp4 [25].

*Proposal 4* (not shown in Fig. 7) The most recent proposal [101] is that vasomotion, waxing and waning contraction of the smooth muscle fibres in the arterial wall, propels fluid towards the brain surface along the basement membranes of the vessel wall. This proposal does not seek to explain the rapid influx of markers along arterial walls, possibly by a different pathway.

#### Is movement within the parenchyma determined by diffusion or by flow from periarterial to perivenular spaces?

It is unclear how the flow required for the glymphatic hypothesis to be correct, at least 0.6 µL g^−1^ min^−1^ (see Proposal 3 above), could be driven through the parenchyma. Jin et al. [77] and Holter et al. [80] have calculated fluid flows within the parenchyma using, respectively, 2-D and 3-D models of the geometry and dimensions of the interstitial spaces. Jin et al. concluded that “little or no advective solute transport is predicted to occur with physiological paravascular pressure differences” taken to be < 5 mmHg. (Strictly advection corresponds to flow while convection includes both flow and diffusion). Furthermore they concluded that the water permeability of the endfeet membrane facing the microvessels, i.e. the membrane containing Aqp4, could have little direct effect on water flow into the parenchyma.^7^ Jin et al. assumed that the ISF between the cells behaves as a free fluid with the viscosity of water. If instead ISF in the interstitial spaces in the brain has properties similar to those of extracellular fluid in tissues in the rest of the body (see [138, 139], discussion in [41] and,^8^ the pressure required for flow would be much larger than that calculated by Jin et al. making bulk flow (advection) even less likely (compare [140]).

Holter et al. [80] have investigated what they consider to be a more realistic model of the parenchyma than that evaluated by Jin et al. One aspect is undeniably more realistic, it treats movement in three dimensions rather than two. It is also asserted that treating the obstacles to flow as being much smaller and more numerous than in Jin et al’s simulation produces a more faithful result. Jin et al. used barriers sized like cell bodies, while Holter et al. have adopted the smaller objects used in Kinney’s construction of the extracellular space [141], which allows for cell bodies and processes. (Smaller objects may be analogous to the increased resistance to flow resulting from macromolecules dissolved in peripheral extracellular fluid, see Footnote 8). Holter et al. conclude that flow makes a much smaller contribution than calculated by Jin et al. However, while Jin et al. treat the entrance and exit of fluid across the endfoot layers explicitly, this is missing from the treatment given by Holter et al. Given that the conclusion is “no flow” in both studies this difference between them may be of no consequence.

It should be noted that neither Jin et al. [77] nor Holter et al. [80] have considered flow along the basement membranes surrounding capillaries presumably because the total area available for such flow is less than for flow via the interstitial spaces (and flow along basement membranes wasn’t considered in the glymphatic hypothesis). Asgari et al. [73] assumed that the resistance to flow of the basement membranes would be the same as for slabs of ^®^Matrigel of the same dimensions, and on this basis concluded that flow via basement membranes would be less than through the interstitium (compare the discussion in [16]).

That flow through the parenchyma is not needed to explain the delivery of solutes to perivascular spaces was suggested by the results obtained using integrative optical imaging (see e.g. [24, 76, 142, 143]). That technique showed that in apparently isotropic regions of brain the spread of fluorescent indicators appears symmetrical over distances of at least 100 µm from a point source (for examples see [24]), indicating that molecules within ISF can reach perivascular spaces in any direction and in good time by diffusion with no evidence for preferential movement towards either arterioles or venules. However, that technique was applied using a water immersion microscope objective after opening the skull and dura to allow access [142]. The open skull and dura may have perturbed flow in the parenchyma. (There is good evidence that cisternal puncture changes flow in the basal cisterns and subarachnoid spaces [25, 89]). Symmetrical spread has now been convincingly confirmed in a systematic study using both direct observation through a cranial window after injection of fluorescently labelled dextrans and recovery from photobleaching [79]. However, it should be noted that the window was glazed after dye injection and hence only shortly before observations were made.

Smith et al. [79] have also found (1) that the dependence of the rate of movements within the parenchyma on the size of the solute is close to that expected if the movement occurs by diffusion; (2) that, in contrast to the report of Iliff et al. [25], the amounts of solutes entering the parenchyma are similar in Aqp4^+/+^ and Aqp4^−/−^ mice; and (3) that local movement of solutes in the parenchyma is not impaired just after cardiorespiratory arrest. They conclude that “these results do not support glymphatic, convective solute transport in brain parenchyma.” In reply to point (2) a group of researchers have posted an un-refereed summary of their experience that comparing three different Aqp4 knockout transgenic lines, including the cell line used by Smith et al. [79], Aqp4 does support “fluid and solute transport and efflux in brain in accordance with the glymphatic system model” [144]. The role of Aqp4 is discussed further in [140].

Pizzo et al. [16] have looked at the distribution of IgG and much smaller single domain antibodies after cisternal infusion. They found that the antibodies rapidly enter the perivascular spaces of blood vessels of all sizes be they arteries, veins or capillaries. The distribution within the parenchyma was as expected for diffusion including the differences between the profiles for different sizes of fluorescent marker. Further discussion supporting the importance of diffusion over bulk flow in the extracellular spaces of the parenchyma can be found in [40]. Perivascular solute movements are considered further in Sect. 5.7.1.2.

#### Is there a glymphatic circulation?

The answer depends partly on what one means by glymphatic circulation. If the meaning is “Convective glymphatic fluxes of CSF and ISF propel the waste products of neuron metabolism into the paravenous space” [136], then the answer is almost certainly no (compare [40, 140], though it should be noted that [54, 137] still argue in favour of the original glymphatic hypothesis). However, if glymphatic circulation is taken to mean only that there is a net inward periarterial flow, a net outward perivenous flow, and some connection between them, then the answer still isn’t known with any certainty. The results discussed above [24, 76, 79, 142, 143] provide powerful experimental support for the widely held view that a glymphatic circulation is not needed to explain solute movements over the short distances that are important in the parenchyma. Furthermore the calculations of Asgari et al. [73, 78], Jin et al. [77] and Holter et al. [80] (see also Footnote 8) suggest that flow through the interstitial spaces of grey matter or along the basement membranes of microvessels in the parenchyma is negligible. However, it is not yet clear that the available *experimental* results exclude the possibility that there is a net flow between the perivascular spaces of arterioles and venules that is large enough to complete a recirculation pathway inwards from CSF via periarterial routes and back to CSF via perivenous routes.^9^ If that flow exists it could be important for transport of solutes over the relatively large distances encountered along the perivascular spaces (see e.g. [76]) while still being negligible relative to diffusion for transport over the relatively short distances within the parenchyma. Interestingly this scenario was proposed recently by Coles et al. [1] (see also Iliff et al. [145]) based on detailed consideration of the evidence available even before publication of the results in [16, 79].

While there have now been hundreds of references to the glymphatic mechanism, almost all of these treat it as accepted dogma and do not test the assumptions or the evidence on which it is based. At present it would be better to refer to perivascular elimination and delivery of substances without prejudice to the mechanism(s) by which these are achieved.

### Variation between sleep and wakefulness

In the comparative studies undertaken on sleeping and awake mice by Xie et al. [128] there were differences in clearance and in interstitial fluid volume in the two physiological states. In these studies, inulin was used as the marker solute for perivascular clearance and the real-time iontophoresis method [74] was used to assess the volume. Briefly Xie et al. [128] found that, in the change from sleep to wakefulness, ISF volume decreased by 1.6-fold, the rate constant for efflux of inulin decreased 2.7-fold and from these values it could be estimated that inulin clearance decreased 4.3-fold (see Section 2.4 in [146]). Changes in the rate of access into the parenchyma of markers added to CSF and the discrepancies between the results of Xie et al. and of Gakuba et al. [147] are discussed briefly in.^10^

As discussed in [146] it is at present unclear whether any change in perivascular clearance of inulin in the transition from sleep to wakefulness is a consequence of the change in ISF volume in the parenchyma or some other effect. There are other possible effects of sleep versus wakefulness that might plausibly alter the clearance, e.g. changes in the shape or volume of either the perivascular spaces or the glial endfeet surrounding them.

## The blood–brain barrier

The blood–brain barrier is more selective than the perivascular pathway in what can and cannot permeate. This selectivity arises from the properties of the endothelial cells surrounding the microvessels. The brain is highly vascularized and cells within the parenchyma are usually within 20 µm of a microvessel [148]. Diffusion over distances this short is rapid. To reach the microvessel, substances must also cross the surrounding layer composed of glial endfeet. This is normally possible because the gaps between the endfeet are not sealed by tight junctions [149, 150]. Even the almost complete coverage of the endothelial cells by glial endfeet proposed by Mathiisen [149] leaves sufficient gaps (see Footnote 7). Thus normally it is the endothelial cells that are the site for the rate limiting steps in efflux across the blood–brain barrier. The current state of knowledge about the role of the endfeet was considered further in [4].

### Passive, non-specific transfer across the blood–brain barrier

There are two possible routes for passive, non-specific transfer across the microvascular endothelial layer, through the cells or around them. The paracellular pathway is “blocked” by the presence of tight junctions but this pathway may still be the principal route for the passive fluxes of small solutes that are barred from the transcellular route by being too polar (mannitol, sucrose and inulin are considered in Appendix B). In addition to neutral molecules like mannitol, the paracellular pathway may be measurably permeable to Na^+^ and Cl^−^ [151]. As discussed in detail in [4] and in Sect. 5.6 evidence for this includes the observation that the tracer fluxes of Na^+^ and Cl^−^ are not affected by ouabain [152] or bumetanide [153], agents that specifically inhibit ion transporters known to be involved in transcellular fluxes of these ions.

**Fig. 8:**
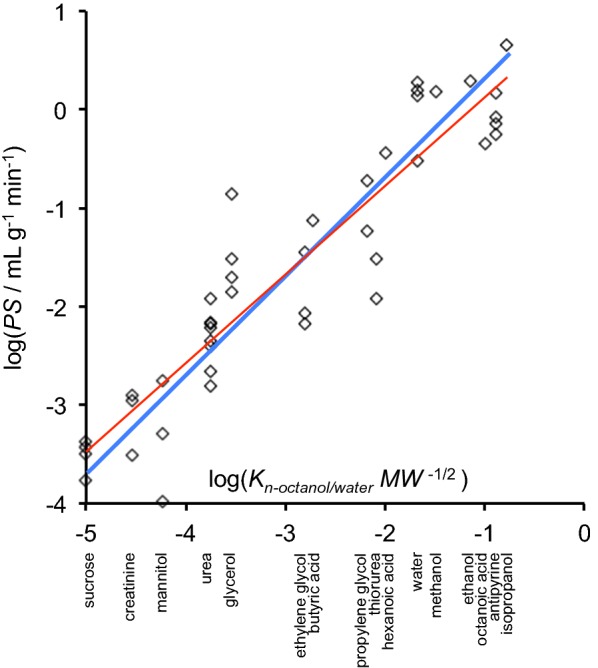
Plot of log(*PS*/mL g^−1^ min^−1^), versus log(*K*_*n*-*octanol*/water_ MW^−1/2^) for the substances indicated along the abscissa. *PS* is the product of permeability and surface area for the blood–brain barrier, *K*_*n*-*octanol*/water_ is the octanol/water partition coefficient and MW is the molecular weight of the substance. The slope of 1 for the heavy blue line indicates *PS* proportional to *K*_*n*-*octanol*/water_ MW^−1/2^. A closer fit to the data can be obtained by allowing the slope to vary, shown as the thin red line, but the improvement in fit is not statistically significant (F = 2.33, p = 0.11, n = 43, extra sum of squares F test [640]) (Data read from Figure 8 of [159])

Almost all of the passive, non-selective permeability of the blood–brain barrier to molecules more lipophilic than mannitol is the result of their ability to diffuse across both the cell membranes and the interior of the endothelial cells. Strong indications that such a physical mechanism applies are the observations: that transport does not saturate, that it is not inhibited by competition by other transported substances, and that no specific inhibitors have been found. Small neutral substances that are able to enter and leave the brain parenchyma by this mechanism include water, methanol, ethanol, isopropanol, glycerol, ethylene glycol, urea and thiourea (see Fig. 8).

Most studies of the passive permeability of the blood–brain barrier have focussed on influx, because it is easier to measure and has obvious importance for the delivery of agents and drugs to the CNS (see e.g. [57, 154]). However, passive permeability allows both influx and efflux and thus these studies are directly relevant to understanding how substances are eliminated from the parenchyma.

In the simplest view the rate limiting steps in the transcellular, passive, unmediated transfer of substances can be thought of as occurring by dissolution in a liquid hydrophobic core of the membranes and diffusion through it. For molecules not much larger than those of the solvent the diffusion constant for the various compounds is taken to be inversely proportional to the square root of their molecular weights [155–157]. The exact relationship assumed is not critical because the dominant factor determining the relative permeabilities is the free energy cost of the transfer from water into the core of the membrane, Δ*G*_*membrane*/water_. This cost determines the relative concentrations in the membrane and the aqueous phase,

5 $$\frac{{c_{\text{membrane}} }}{{c_{\text{water}} }} = K_{{{\text{membrane}}/{\text{water}}}} = e^{{{{-\Delta G_{{{\text{membrane}}/{\text{water}}}} } \mathord{\left/ {\vphantom {{\Delta G_{{{\text{membrane}}/{\text{water}}}} } {RT}}} \right. \kern-0pt} {RT}}}}$$

where *K*_*membrane*/water_ is the partition coefficient, *R* the universal gas constant, and *T* the absolute temperature. The free energy cost and the partition coefficient are usually estimated by assuming that the membrane core can be described as being like a layer of n-octanol (see [158, 159] and for more recent discussions [160, 161]), and thus

6 $$\frac{{c_{\text{membrane}} }}{{c_{\text{water}} }} \propto K_{{n{\text{-octanol}}/{\text{water}}}} = e^{{-\Delta G_{{n{\text{-octanol}}/{\text{water}}}} /RT}} .$$

It is likely that n-octanol rather than, say, n-octane is appropriate as a model for the membrane interior because the –OH group can participate in hydrogen bonds.

Fenstermacher [159] reviewed the studies up to 1984 with the result summarized in a plot of log[*PS*] versus log[*K*_*n*-*octanol*/water_ MW^−1/2^] (see Fig. 8) where *PS* is the permeability surface area product for brain capillaries. For the substances listed in the figure, which have simple structures and molecular weights less than 200, the slope of the loglog plot is not significantly different from 1, i.e. PS appears to be proportional to *K*_*n*-*octanol*/water_ MW^−1/2^.

There have been many other reports based on studies using more complicated or larger molecules. These have usually reported a linear relation between log(*PS*) and either log[*K*_*n*-*octanol*/water_] or log[*K*_*n*-*octanol*/water_ MW^−1/2^] but often with a slope substantially less than 1 (see e.g. [162, 163]). It should be emphasized that slope not equal to 1 means that the fluxes are not proportional to *K*_*n*-*octanol*/water_ MW^−1/2^ and thus, for at least some of the substances tested, simple diffusion and partition into an environment that looks like n-octanol are not the only important factors that need to be considered. The appropriate factors are considered further in Appendix C.

Correlating the passive permeabilities for substances at the blood–brain barrier with their partition coefficients for transfer from water to n-octanol has the virtue of focussing attention on the most critical aspect of the passive permeation process, the free energy cost of removing the solute from water and inserting it into a relatively hydrophobic environment. However, these correlations have been thought too imprecise to use as a criteria for selecting candidates to consider in a drug discovery setting. There have been many attempts to do better, some in terms of a set of rules analogous to the “rule of 5” for intestinal absorption [164], some using better estimates of the free energy cost for solutes to reach the rate limiting step of the transport, and some using a mixture of both.

To obtain better estimates of the free energy, Abraham and colleagues (see [165–168]) have employed linear free energy relations, LFER, to calculate correlations based on a two step process. First quantitative “descriptors” of the molecules under consideration are chosen without regard to the process of interest. Then, once the descriptors have been chosen, the relevant free energy changes for processes such as partition into a solvent or permeability across the blood–brain barrier, are calculated as linear sums of the descriptors with coefficients that depend on the process but not on the molecules (see e.g. [160, 165, 166]. Having used data for some substances to calculate the LFER coefficients, these can then be used for other substances. This approach has been applied with considerable success to partition into solvents for many more molecules than are needed to calculate the coefficients [165]. It has also allowed closer prediction of blood–brain barrier permeabilities than the simple solubility-diffusion model [166, 167] (see Appendix C).

There is, however, a danger in adopting this approach to the prediction of permeability. The use of linear free energy relations reveals correlations between the descriptors and the rate of transport, but unless used carefully it can obscure important features of the mechanism. For instance in the correlations reported for log(*PS*) [166, 167], the strongest correlation was a positive correlation between molecular volume and permeability, i.e. this approach seems to say that increases in molecular size result in increased permeability [160, 167]. However, the idea that bigger objects will be more permeable because they are bigger is completely counter-intuitive. The likely explanation for this paradox is simple. For the molecules considered in the correlations, increases in molecular volume were associated with large increases in lipophilicity as measured by *K*_*n*-*octanol*/water_ and it is plausible that it was the increase in lipophilicity that increased the permeability. Indeed as shown in Appendix C Abraham’s descriptor approach predicts for the compounds tested [166] that log[*PS/K*_*n*-*octanol*/water_] varies much less than log[*PS*] and furthermore that it decreases when molecular volume is increased. In terms of Fig. 8, because large values of *K*_*n*-*octanol*/water_ are associated with large molecules, slopes less than 1 are expected if increasing molecular size has some effect that decreases permeability in addition to its effect that increases permeability by virtue of increasing *K*_*n*-*octanol*/water_ (see Appendix C).

Liu [169] investigated the utility of many different descriptors for predicting log(*PS*) for neutral molecules and settled on three, log(*D*), TPSA and vas_base where *D* is *K*_n-octanol/water_ measured specifically at pH 7.4, TPSA is the polar surface area of a molecule, which correlates with the ability to form hydrogen bonds (compare [170]), and vas_base is the surface area of basic groups.

Abraham [168, 171] has presented the extension of the LFER approach to ions.^11^

Fong [161] has reviewed many of the attempts to predict permeabilities of the blood–brain barrier to solutes. He concludes that the most important factors for neutral solutes are: the free energy required to remove the solute from water; the free energy gained from the interactions of the solute with the membrane core, usually modelled by its interaction with n-octanol; the dipole moment of the solute; and lastly its molecular volume. Increases in molecular volume per se decrease permeability. Geldenhuys et al. [172] has provided many useful references in a review prepared from the perspective of the utility of predictions in high-throughput screening.

### Transporters at the blood–brain barrier

The membranes of the endothelial cells that constitute the blood–brain barrier possess transporters for many different types of solutes. These transporters may be present on luminal, abluminal or both surfaces of the endothelial cells. Prominent among them are transporters for common nutrients and waste products of metabolism: GLUT1 for glucose, MCT1 for lactic acid and other small monocarboxylic acids, a range of transporters for amino acids, and several for nucleosides. There are also ion transporters involved in maintenance of the ionic composition of the brain fluids. Many of the transporters are specific and are involved in moving the normal constituents of brain extracellular fluid. Some of these are considered in Sect. 5. In addition there are also less specific transporters. Many of these can mediate efflux of a variety of other substrates including many exogenous substances and toxic occasional products of metabolism.

Evidence concerning the presence and identity of many of these transporters has been reviewed elsewhere with studies being conducted primarily at the level of transcript [173–178], protein [31, 44, 58, 176, 179–188] and/or function [4, 20, 31, 46, 55–57, 179, 189–200]. The reports by Roberts et al. [180] and Kubo et al. [58] and reviews by Hawkins et al. [44], Redzic [31], Campos-Bedolla [57], Worzfeld and Schwaninger [187] and Nalecz [200] have been useful as sources of information about the localization of transporters to the luminal or abluminal membranes.

This review will not seek to provide yet another comprehensive survey. Extensive lists of transporters and substrates are available in many of the cited references and for SLC transporters at the BioParadigms website [201, 202].

#### ABC efflux transporters

It has long been appreciated that the brain represents a pharmacological sanctuary and is selectively “protected” from the toxic effects of many chemotherapeutic agents. These include vincristine and doxorubicin (aka adriamycin), which fail to penetrate the blood–brain barrier as well as their lipid solubilities would suggest [162]. A major part of this failure to penetrate has since been attributed to the presence of the multidrug transporter, *P*-glycoprotein. Absence of this transporter in knock-out mice was shown to allow entry of toxic agents including ivermectin [203]. *P*-glycoprotein was found to be located in the luminal membrane (see e.g. [204–209]) of the endothelial cells and is believed to act there to transport substrates out of the cells so rapidly that little remains to penetrate the abluminal membrane and enter the brain.

It is believed by many that *P*-glycoprotein, a transmembrane protein, acts by removing its lipophilic substrates from the lipid layer of the cell membrane, depositing them back into the blood [210–213]. Its structure has been investigated in both substrate-free and inhibitor bound conformations [213] and binding sites for various of its many substrates identified within the large cavity seen in the substrate-free conformation. It is the binding and hydrolysis of ATP that provides the motive force leading to a large conformational change in the *P*-glycoprotein and the transfer and expulsion of its substrates. There are two ATP binding sites located on the cytoplasmic side of the protein.

*P*-glycoprotein, otherwise called ABCB1, is a member of the ABC (ATP-Binding Cassette) family of proteins many of which are primary active transporters that utilize the hydrolysis of ATP to fuel substrate transport. Since its discovery, other ABC active transporters with broad substrate profiles have been found in the luminal membrane of the endothelial cells. These include Breast Cancer Resistance Protein, BCRP (ABCG2) [180, 197, 214–218] and Multidrug Resistance Proteins, MRPs 4 and 5 (ABCC4 and 5) [180, 197, 209, 218–221]. MRP1 (ABCC1) has also been implicated but levels of this transporter are thought to be low in brain endothelial cells in situ and only increase in cultured brain endothelial cells once they are removed from the brain microenvironment [180, 184, 218, 222–226]. MRP1 and MRP2 are apparently upregulated and clearly expressed in epilepsy [227–229].

The role of efflux from endothelial cell to blood by ABC transporters in preventing influx of many substances from blood into the brain has been extensively reviewed (see e.g. [57, 196, 197, 199, 221, 230–234]. The regulation of *P*-glycoprotein, BCRP and MRP2 at the blood–brain barrier has been reviewed by Miller [221].

**Fig. 9:**
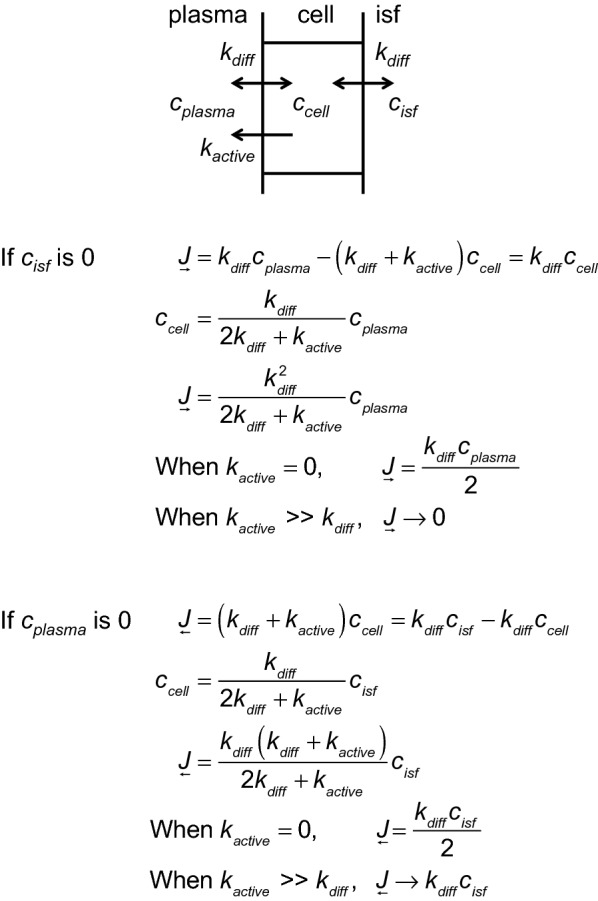
The influence of ABC transporters on the movements of lipophilic substances. The substance is presumed to be able to enter and leave the endothelial cells by diffusion with rate constant *k*_*diff*._, which for simplicity in this example is assumed to be same on both sides. The substance is expelled from the cell by ABC transporters on the luminal side at a rate, *k*_*active*_*c*_*cell*_. With these assumptions the effect of the ABC transporters on influx can be calculated by setting *c*_*isf*_ = 0 and the effect on efflux by setting *c*_*plasma*_ = 0. In both sets of equations, the first line states that at steady-state the net flux into the cell on one side must equal the net flux out of the cell on the other. From the next to the last lines of both sets of equations, if the rate of ABC mediated expulsion from the cell is small or zero, the rate constants for both influx and efflux are (*k*_*diff*._/2). By contrast from the last lines if the rate of ABC mediated expulsion is large, influx to the brain, $$\underset{\raise0.3em\hbox{$\smash{\scriptscriptstyle\rightarrow}$}}{J}$$, becomes very small, while efflux from the brain, $$\underset{\raise0.3em\hbox{$\smash{\scriptscriptstyle\leftarrow}$}}{J}$$, is doubled compared to the efflux with no ABC transporter

The role of ABC transporters in efflux from the brain parenchyma differs depending on the nature of the substrate. As described in Fig. 9, for substances that are sufficiently lipid soluble to cross the endothelial cell membranes rapidly by passive transport, the presence of ABC efflux transporters can greatly reduce blood-to-brain influx, as observed experimentally. However, as also explained in Fig. 9 the ABC transporters in the luminal membrane will have only a modest effect, e.g. a doubling, on the rate of brain-to-blood efflux. This may be of little consequence as the rate of efflux for lipid soluble substances is already high.

The role of ABC transporters for solutes with low passive permeability across the membranes is considered in the next section.

#### Efflux mediated in part by SLC solute transporters

Many of the SLC (solute carrier) transporters (see [202] for a list) are present in the membranes of the endothelial cells of the blood–brain barrier. Some are considered in connection with the transport of specific solutes in Sect. 5. Others, primarily from the SLC21 (OATPs, organic anion transporting polypeptides) and SLC22 (OATs and OCTs, organic anion transporters and organic cation transporters) families are associated with transport of a variety of organic anions and cations. These have been reviewed frequently and extensively [57, 176, 200, 218, 235–246]. (Uppercase labels, e.g. SLC or OAT, strictly refer to human sequences and proteins, while mixed-case labels, e.g. Slc or Oat, refer to any other species. In this review uppercase is also used when there is no intention to specify species).

There is little quantitative data on the efflux of organic anions and cations from the parenchyma in humans though many are known to be transported. In rodents more information is available for transfer of organic anions than cations. Table 1 lists some examples of organic anions/neutral molecules for which brain-to-blood transport rate constants have been determined. These are all believed to be substrates for Oat3 (Slc22a8) and/or one or more of the Oatp transporters present at the blood–brain barrier. In broad terms [238], small hydrophobic anions are substrates for Oats (Slc22 family) while larger amphipathic anions are substrates for Oatps (Slc21 family, whose member names start with Slco, see [247]). For comparison Table 1 also lists rate constants and clearances for examples of markers for perivascular efflux. It is clear that the rates of elimination of the Slc substrates are considerably greater than could be supported by perivascular efflux alone.

**Table 1:** Comparison of rate constants for efflux and clearances for Slc22 and Slco substrates and the perivascular markers inulin, mannitol and sucrose

	*k*_*eff*_/min^−1^	*t*_1/2_/min	*V*_*d*_/mL/g	*CL/*µL g^−1^ min^−1^	Ref #	Notes
Slc substrates						Rat unless stated otherwise
*p*-Aminohippuric acid (PAH)	0.059	12	0.80	47	[558]	Influx much slower than efflux
*p*-Aminohippuric acid (PAH)	0.039	18			[559]	Abl. Oat3 based on inhibitors, lum. Mrp4 possible based on kidney [560]
*p*-Aminohippuric acid (PAH)	0.0175	40			[131]	
Penicillin G, benzylpenicillin	0.043	16			[559]	Oat3 based on inhibitors
Taurocholate	0.023	30			[561]	
BQ-123	0.0078	100			[561]	
Estrone sulfate	0.066	9.9	1.1	75	[562]	
Estrone	0.061	11	3.3	227	[562]	
Dehydroepiandrosterone sulfate (DHEAS)	0.027	26		118	[563]	Influx much slower than efflux, Oatp2
Estradiol-17beta-glucuronide (E217betaG)	0.037	19			[564]	Oatp2, 40% and Oat3 20%
Pravastatin	0.060	12	0.99	59	[565]	Oat3 (Slc22a8) and Oatp2 (Slco1a4) + others
Pitavastatin	0.026	27	14	364	[565]	Oat3, Oatp2 (Slco1a4) +others +diffusion
Homovanillic acid	0.017	40.8			[566]	Oat3 from inhibitors
Indoxyl sulfate	0.011	64			[567]	Oat3 and others
Pemetrexed	0.018	39	0.62	11	[568]	Mouse. Oat3 and unknown (not Mrp2 not Bcrp)
Methotrexate	0.024	29	0.85	20	[568]	Oat3 and Bcrp suggested
Buprenorphine	0.025	27.5	6.1	154	[569]	Pgp and unknown, possibly diffusion
AZT, (3′-azido-3′-deoxythymidine)	0.032	22			[570]	Oat3 from benzylpenicillin inhibition
DDI, (2′,3′-dideoxyinosine)	0.253	2.8			[570]	Oat3 from benzylpenicillin inhibition + diffusion
Markers for perivascular elimination						
Inulin	0.006 awake 0.016 asleep	115 43	0.2*	1.2 3.2	[128]	Mouse
Inulin	0.003	230	0.2*	0.6	[62]	Mouse
Inulin	0.005	135	0.2*	1	[131]	Rat
Mannitol	0.004	170	0.2*	0.8	[25]	Mouse
Sucrose	0.0028 awake 0.0043 anesth.	245 160	0.2*	0.56 0.86	[131]	Rat
Albumin	0.006	115	0.2*	1.2	[131]	Rat
Dextran-10K	0.0035	197	0.2*	0.7	[131]	Rat
Dextran-70K	0.004	170	0.2*	0.8	[131]	Rat

*k*_*eff*_ rate constant for efflux determined from the time course of the decrease in concentration after injection of solute into the parenchyma (brain efflux index for the Slc substrates relative to inulin; these underestimate *k*_*eff*_ for values less than ~ 0.01 min^−1^ see [131]); *t*_1/2_ = 0.69/(rate constant) is the half-life; *V*_*d*_, volume of distribution in the parenchyma determined using brain slices (ISF volume for inulin and mannitol); *CL* = *V*_*d*_ × *k*_*eff*_, the clearance. For the Slc substrates more than one transporter in each membrane is likely to be involved in the transport

* Assumed equal to ISF volume

**Fig. 10:**
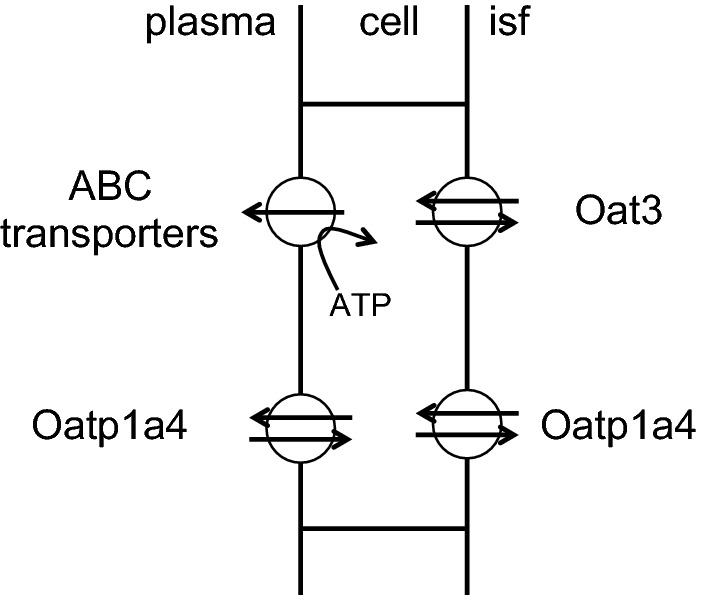
Transport of organic anions across the blood–brain barrier. Organic anion transporters at the blood–brain barrier. The principal known transporters in the rat are shown. In human OAT3 is abluminal, while both OATP1A4 and OATP2B1 are present on both membranes. The ABC efflux pumps, *P*-gp, BCRP, MRP4 and MRP5, are all localized to the luminal, plasma facing, membrane. The Oat and Oatp transporters are exchangers (see Footnote 12). Localizations from [180] and the references in Table 1

As indicated in Fig. 10 transport from the parenchyma into the endothelial cells occurs via one or more of the SLC transporters, while exit from the endothelial cells to plasma occurs via either SLC or ABC transporters. For many of the anions efflux from brain to blood is clearly an active uphill process suggesting that the ABC route is dominant (for a caveat see.^12^) Transport across either membrane can be rate limiting and in many cases transport across each can occur by more than one route. As a consequence demonstration that a specific inhibitor of a transporter reduces the rate of efflux is evidence for involvement of that transporter, but failure to inhibit is relatively uninformative.

For the SLC substrates in Table 1 the half-lives are shorter than the 1–2 h characteristic of markers eliminated from the parenchyma by perivascular efflux (see Sect. 3). As noted earlier, shorter half-lives imply that there are mechanisms for elimination other than perivascular. This is reinforced by noting that the clearances for those solutes for which volumes of distribution are available are much greater than the clearance associated with the perivascular route (see Sect. 3.2). There is ample further evidence (see the references for the entries in Table 1) for the importance of the Oat and Oatp transporters in the elimination of these solutes from the parenchyma including saturation, competition, the availability of transport inhibitors, and the rapid appearance of effluxed material in venous blood draining the head.

### Efflux by transcytosis

Transcytosis is much less prevalent across the endothelial cells of the blood–brain barrier than across those of peripheral capillaries [248–251]. Nevertheless both adsorptive mediated transcytosis (AMT) and receptor mediated transcytosis (RMT) are still likely to be important mechanisms for the transfer of some large substrates across the blood–brain barrier. The initial event in AMT is the adsorption of usually positive substrates onto the surfaces of caveolae, while that for RMT is binding of the substrate to specific receptors that are in or become incorporated into clathrin coated pits. In both cases at the blood–brain barrier this leads to endocytosis followed by delivery of a substantial fraction of the contents of the resulting vesicles to the opposite membrane for exit, possibly by exocytosis [49, 63, 252]. AMT is thought to account for much of the influx into the brain of histones [253], “cell penetrating peptides” [49, 251, 254], HIV [255, 256], and cargos conjugated to the lectin wheat germ agglutinin [257] and to underlie the increase in “generalized permeability” caused by protamine [258]. The downsides of AMT are that it is relatively non-selective for substrates [256] and that it occurs in many cells throughout the body. In addition there is little if any evidence that it occurs in the direction from brain to blood [257, 259]. While RMT also occurs throughout the body, transport by this mechanism depends on interaction of the substrates with specific receptors that may be found primarily in specific locations such as the blood–brain barrier. In addition there is evidence that RMT can occur in either direction, i.e. from brain to blood as well as from blood to brain.

AMT and RMT in the direction from blood to brain have been studied extensively as routes of entry to the brain for endogenous substrates, but even more in the context of mechanisms for drug delivery. These studies have been reviewed frequently [57, 64, 154, 249, 252, 260–266]. However, even so, the steps occurring after the initial endocytosis remain only partially understood [63, 249, 250, 262, 267, 268] including even the answer to the important question of whether the cargo is released within the cell or delivered to the far side by exocytosis. By contrast evidence for transport via transcytosis in the direction brain to blood has been reported for only a few systems including transport of amyloid-β peptides via interaction with LRP1 (low density lipoprotein receptor related protein 1) and LRP2 (low density lipoprotein receptor related protein 2) (see Sect. 5.7), of IgG antibodies via interaction with an unidentified receptor [269–275] and of transferrin [60] via interaction with the transferrin receptor (TfR) [61] (see below).

Transport of transferrin is closely related to transfer of iron. Iron in plasma and in brain extracellular fluid is present almost entirely complexed to transferrin i.e. as holo-transferrin. It has long been known that iron and transferrin enter the brain across the blood–brain barrier and it was originally hypothesised that they are transferred together by endocytosis followed by exocytosis, i.e. direct transcytosis, of holo-transferrin (see e.g. [61, 276]). Yet there have been arguments against this idea arising from dual labelling experiments showing that far more labelled iron than labelled transferrin accumulates in the brain, see e.g. [60, 277, 278]. In addition it has been argued that release of holo-transferrin from TfR is unlikely to occur as there needs to be prior dissociation of iron for release of transferrin from its receptor [279]. So though there is general agreement that holo-transferrin interacts with TfR, which then mediates endocytosis of the iron/transferrin/receptor complex into the endothelial cells, there has been controversy over the subsequent steps in the transfers of transferrin and iron into the brain. Assuming that holo-transferrin is indeed directly transcytosed across the blood–brain barrier, then the limited net entry observed of transferrin to the brain implies that there must be transcytosis of transferrin without iron, apo-transferrin, back out of the brain. Alternatively if the iron is dissociated from the transferrin within the endothelial cells, it is likely that there is exocytosis of apo-transferrin on both sides of the cells (see [280–282] and the footnote^13^ for further discussion).

Little is known about transport of transferrin out of the brain. There have been reports that labelled apo-transferrin injected into the brain can be transported from brain to blood, but it is not clear how important this is under normal conditions. Banks et al. [60] found that the apo-transferrin was removed from the brain faster than albumin, implying the existence of a route other than washout via CSF. However, subsequently Moos and Morgan [278] did not confirm this result. By contrast Zhang and Pardridge [61] found an early component of loss of injected apo-transferrin, half-life 39 min, which was much faster than that for loss of injected 70 kDa-dextran. Furthermore this rapid component was inhibited by cold apo-transferrin, i.e. there was competition, with an apparent dissociation constant of less than 30 nM implying interaction with a specific receptor which was presumed to be the receptor protein detected by OX26, i.e. TfR. As these studies on transferrin efflux are substantially older than the studies on iron uptake linked to transferrin, further investigation of transferrin transport from brain to blood might be informative.

## Clearance of specific substances

There are certain species that are critical for normal brain function and that must be transported into or out of the brain rapidly and in large quantities. The most prominent of these are O_2_, CO_2_, water and glucose. Influx and efflux of these species are so rapid that they entail movements of a large fraction of the amounts flowing through the brain vasculature, much more than could be delivered by the blood flow to just the choroid plexuses.

### Water

Water permeability of the blood–brain barrier can be calculated in two very different ways. In the first tritiated water is introduced into the blood and the permeability, *P*_*w*,*tracer*_ calculated from the ratio of the undirectional influx of tracer, *J*_*inf*_, to the concentration of the tracer, *c*_*THO*_,

7 $$P_{w,tracer} = {{J_{\inf } } \mathord{\left/ {\vphantom {{J_{\inf } } {c_{THO} }}} \right. \kern-0pt} {c_{THO} }}.$$

It is assumed that this permeability also applies to efflux and to unlabelled water. This permeability is often called the diffusional water permeability, *P*_*d*_. The major difficulty with this method is that the influx is so great that 70–90% of the tracer arriving in the blood enters the parenchyma in a single pass (see chapter 4 in Bradbury [55]  and [283–289]). Thus along much of the length of the microvessels the concentration gradient of the tracer across the microvessel walls driving its influx is much less than the concentration that was added to the blood. The permeability calculated from Eq. 7 using the arterial concentration of the tracer thus seriously underestimates the true water permeability of the blood–brain barrier. Mathematical expressions to correct for this effect have been derived relating the fraction of the tracer extracted from the flow through the blood vessel to the *PS* product (reviewed in [159]). However, even after correction the calculated values are inaccurate when the extraction fraction is large. Paulson et al. [290] found values about 1/5th of the PS values calculated from osmotic flow as described below and similar values have been determined by others (see [159]).

The second method for measuring water permeability uses an osmotic gradient to generate a net flux, *J*_*net*_, of water across the barrier. In effect a water concentration gradient is produced by “diluting” or “concentrating” the water on one side by adding or removing solutes and the permeability is then calculated as

8 $$P_{{w,{\text{osmotic}}}} = {{J_{net} } \mathord{\left/ {\vphantom {{J_{net} } {\Delta c}}} \right. \kern-0pt} {\Delta c}}$$

with results close to 1.1 × 10^−3^ cm s^−1^ for both rats [291, 292] and humans [290]. (The original references and a recent review [4] can be consulted for the actual equations used which are based on arguments that avoid the rather woolly concepts of “diluting” and “concentrating” the water). Using *S* = 100 cm^2^ g^−1^, the value of the surface area of the microvessels employed in [290, 292], the permeability-area product, *PS*, i.e. the clearance, is ~ 0.11 mL g^−1^ s^−1^ = 6.7 mL g^−1^ min^−1^. Patlak and Paulson [293] have argued that for the blood–brain barrier the tracer value is likely to be a better estimate of the true water permeability because the measurement of osmotic permeability using a brief exposure to raised osmolality reflects partly water extraction from the endothelium rather than from the parenchyma. It is adequate for the present purpose to use the two estimates as brackets of the correct value.

Water influx and efflux across the human blood–brain barrier each amount to roughly 40,000 mol day^−1^. The difference between the influx and efflux is *very* much less. Not even the normal direction of the net flux of water across the blood–brain barrier is known with any certainty, partly because it is so small. The available evidence suggests that scaled for a human there is a net movement from blood to brain amounting perhaps to ~ 10 mol day^−1^ (see [4]). For comparison metabolic production of water within the brain is ~ 3.3 mol day^−1^ and the amount of water in the CSF produced by the choroid plexuses is ~ 28 mol day^−1^.

### Carbon dioxide

It has long been known that CO_2_ crosses the blood–brain barrier sufficiently rapidly that its removal from the parenchyma is largely blood-flow limited (see Sect. 6.1), i.e. pCO_2_ in the venous effluent is closer to that within the parenchyma than to that in arterial blood. Rapid transfer between blood and brain has been confirmed directly by the observation that when CO_2_ labelled with the short-lived isotope ^11^C is added to arterial blood more than 70% is extracted from the cerebral blood flow in a single pass [294] (see Section 6.4.2 in [4] for further discussion).

A crude underestimate of the clearance for CO_2_ in humans can be calculated from the rate of CO_2_ production (in turn calculated from glucose and oxygen consumption) [295, 296], ~ 3.3 mol day^−1^, and the average difference in pCO_2_ between ISF and plasma along the length of the microvessels which must be less than the difference between the values in the parenchyma and arterial blood, ~ 8 mmHg [297]. 8 mmHg corresponds to a difference in free concentration of 0.24 mM [298] and thus the underestimate of the clearance for a 1400 g brain becomes

9 $$CL > {{\left( {2200\;{{\upmu{\text{mol}}\;{ \text{min} }^{ - 1} } \mathord{\left/ {\vphantom {{\upmu{\text{mol}}\;{ \text{min} }^{ - 1} } {1400\;{\text{g}}}}} \right. \kern-0pt} {1400\;{\text{g}}}}} \right)} \mathord{\left/ {\vphantom {{\left( {2200\;{{\upmu{\text{mol}}\;{ \text{min} }^{ - 1} } \mathord{\left/ {\vphantom {{\upmu{\text{mol}}\;{ \text{min} }^{ - 1} } {1400\;{\text{g}}}}} \right. \kern-0pt} {1400\;{\text{g}}}}} \right)} {0.24}}} \right. \kern-0pt} {0.24}}\;\upmu{\text{mol}}\;{\text{mL}}^{ - 1} = 6.5\;{\text{mL}}\;{\text{g}}^{ - 1} \;{ \text{min} }^{ - 1} .$$

This is more than 5000 times larger than would be possible by perivascular clearance, which simply restates that the clearance of CO_2_ must be across the blood–brain barrier.

### Glucose

**Fig. 11:**
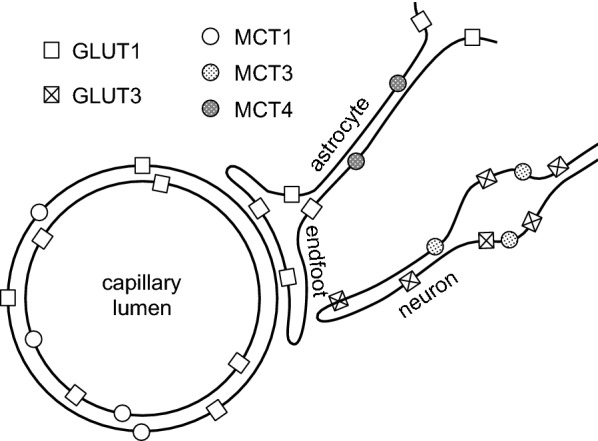
GLUT and MCT transporters at or near the blood–brain barrier. GLUT1 and MCT1 are present on endothelial cells; GLUT1 and MCT4 on astrocytes (Figure simplified and redrawn from Simpson et al. [315])

Glucose and O_2_ are the most important substrates for brain energy metabolism. Glucose enters ISF across the blood–brain barrier via the more glycosylated form of a passive, selective carrier, GLUT1 (SLC2A1), that is present in membranes located on both surfaces of the endothelial cells. From ISF it rapidly enters both astrocytes by the less glycosylated form of GLUT1 and neurons via GLUT3 (see Fig. 11). The rate-limiting step in glucose metabolism is the effectively irreversible phosphorylation by hexokinase. Normally glucose influx into the parenchyma is higher than the rate of phosphorylation, and thus there must be some efflux corresponding to the difference. This efflux is also primarily across the blood–brain barrier via GLUT1. Because both influx and efflux of glucose take place by passive transport there is no additional metabolic cost caused by having influx greater than the metabolic rate.

It has long been known that glucose is able to cross the blood–brain barrier rapidly [189, 299–302]. Crone [299] found that at low concentrations as much as 50% of the glucose arriving in the arterial blood could be extracted in a single pass, but that this percentage decreased with concentration, falling to 28% at 5 mM and ~ 14% at 14 mM. This extensive but saturable transport implies the presence of a specific transporter, which as stated above is GLUT1 (SLC2A1) [303–305].

The expression of GLUT1 in the endothelial cell membranes has been measured in several different ways: by cytochalasin-B binding, by specific antibody binding, and by proteomic methods (see Table 2 for references). In the proteomic studies from the group of Terasaki, Uchida, Ohtsuki and colleagues, GLUT1 was found to be the most highly expressed of all the transporters that are present in the membranes of the endothelial cells [306].

**Table 2:** Expression of GLUT1 at the blood–brain barrier

Source	Method^a^	Species	Expression/pmol mg^−1^		
			Luminal	Total^b^	Abluminal
			Relative to microvessel protein		
Dick et al. [303]	Cytochalasin B binding	Rat, pig		69–80	
Kalaria et al. [571]	Cytochalasin B binding	Human		42	
Farrell and Pardridge [572]	Immunogold e.m	Rat	12%^c^	100%^c^	48%^c^
Cornford et al. [573]	Immunogold e.m	Human	48%	100%	18%
Vannucci et al. [574]	Cytochalasin B binding	Rat		40–125	
Kamiie et al. [182]	Proteomics	Mouse		90	
Uchida et al. [184]	Proteomics	Human		139	
Shawahna et al. [176]	Proteomics	Human		78.5	
Hoshi et al. [185]	Lysate digestion proteomics	Rat		84–98	
			Relative to membrane protein		
Simpson et al. [575]	Fractionation, cytochalasin B binding	Bovine	620		280
Kubo et al. [58]	Fractionation, proteomics	Porcine	79%		21%
Zhang et al. [188]	Proteomics	Porcine		300	

^a^Fractionation = fractionation of isolated plasma membranes

^b^For immunogold detection, values are percentages of the immunogold particles where the total includes cytoplasmic

^c^Antigen for the antibody used by Farrell and Pardridge appears to be partially masked for GLUT1 most markedly in the luminal membrane in bovine endothelial cells [575]

A rough estimate of the glucose clearance in man can be calculated from the rate of consumption, about 0.55 mol day^−1^ = 380 µmol min^−1^ [295, 296] or, for a 1400 g brain, 270 nmol g^−1^ min^−1^. For a difference between the concentrations in plasma and ISF of 5 mM this corresponds to *CL* ~ 54 µL g^−1^ min^−1^. In isolated perfused dog brains Betz et al. [302] measured the loss of glucose from the blood flow through the brain and found about 0.6 µmol g^−1^ min^−1^ at 6 mM from which at this concentration *CL* = 100 µL g^−1^ min^−1^. Hawkins [307] lists values ranging from 158 to 352 µL g^−1^ min^−1^ (at 6 mM glucose) depending on brain region (inferior colliculus the highest). Note that the first two of the estimates above are based on the net flux of glucose while the values listed by Hawkins are based on the unidirectional influx. Because all of these estimates far exceed the clearance expected for perivascular efflux, ~ 1 µL g^−1^ min^−1^ (see Sect. 3 and Table 1), the perivascular route is likely to be of minor importance.

Cutler and Sipe [301] using anaesthetized cats, Bachelard et al. [308, 309] using anaesthetized rats and Betz et al. [302] using isolated perfused dog brains all found that the influx of glucose measured using tracers could exceed the net flux by two to threefold. This is a direct, experimental demonstration that there is efflux across the blood–brain barrier that can be as large as two-thirds of the influx. This would of course be less under conditions of increased metabolic demand.

Glucose distributes rapidly between intracellular and extracellular water within the parenchyma and thus its volume of distribution is close to the total aqueous volume, which is *V*_*D*_ = 0. 77 mL g^−1^ [310–315].^14^ Pfeuffer et al. [316] used diffusion weighted NMR to distinguish between intracellular and extracellular glucose and found that only 19% of the glucose in the parenchyma was extracellular which is in agreement with the fraction of water that is extracellular. These observations imply that glucose transport across the membranes of astrocytes and neurons is rapid compared to the rate of metabolism.

When glucose concentrations in plasma are near 6 mM, the average concentration of glucose in brain water is roughly 1.3 mM (see Sect. 5.3.2). Even two fold changes in the concentration in brain water have little effect on the cerebral metabolic rate of glucose, *CMR*_*glc*_, because these concentrations are substantially greater than the *K*_*m*_ for phosphorylation of glucose by hexokinase (0.04–0.05 mM [317–319]) and hence hexokinase, the first step in glucose metabolism, remains nearly saturated (compare e.g. [313]).

It is unclear why the passive glucose transport at the blood–brain barrier is mediated by a carrier rather than by a pore. Pores have the advantage that they do not undergo any large conformation changes during transport of each substrate. Hence they are capable of high turnover numbers, which would seem to be an advantage. On the other hand carriers allow more complicated coupling of transport between different solutes and it is possible that during transport of a relative large solute like glucose, it is easier for a carrier than the “open hole” of a pore to prevent unwanted transfer of other solutes. (Water can probably get through both carriers and pores. The possibility that water permeability of GLUT1 may or may not be important at the blood–brain barrier [320] was considered in Section 4.3.6, footnote 17 of [4]). While arguments for “why a carrier” are speculative, the structural and kinetic evidence, reviewed in the following subsections, leave little doubt but that glucose transport across the membranes of the endothelial cells of the blood–brain barrier is mediated by a carrier.

#### Structure of GLUT1 (SLC2A1) and the kinetics of the glucose transport it mediates in red blood cells

**Fig. 12:**
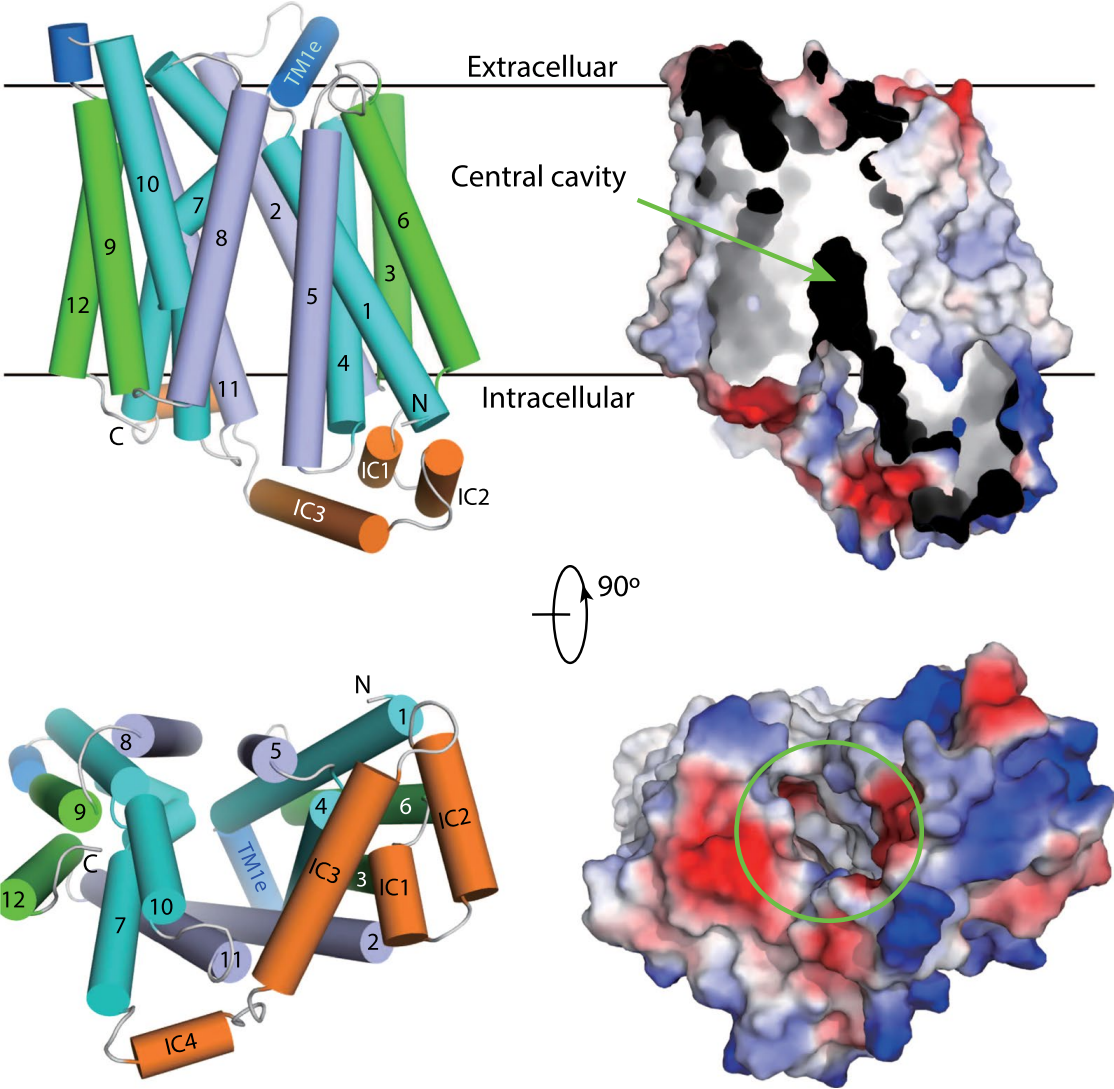
Structure of the human glucose transporter GLUT1. The structure of full-length human GLUT1 containing two point mutations (N45T, E329Q) was determined in an inward-open conformation. The side and cytoplasmic views are shown. The corresponding transmembrane segments in the four 3-helix repeats are coloured the same. The extracellular and intracellular helices are coloured blue and orange, respectively. A slab of cut- open view of the surface electrostatic potential, which was calculated with PyMol50, is shown on the right to facilitate visualization of the inward-facing cavity. IC indicates intracellular helix. Reprinted by permission from Springer Nature from Nature 510, 121–126, Crystal structure of the human glucose transporter GLUT1 by Deng et al. [321]

A crystal structure for GLUT1 has been obtained using a GLUT1 construct purified from an expression system (see Fig. 12) [321]. In this structure a bundle of α-helices spans the membrane surrounding an inner cavity open at the cytoplasmic end. This structure and those for related transporters (for references see [322]) strongly support the widely held view that the transport kinetics should be described using a carrier model (see Appendix D). A binding site in the central cavity of the carrier can be exposed to either side of the membrane, but only one side at a time. While the site is exposed a substrate molecule can associate with or dissociate from the site. The side of exposure can be altered by a conformation change in the carrier and the substrate can then associate or dissociate on the other side of the membrane.

**Fig. 13:**
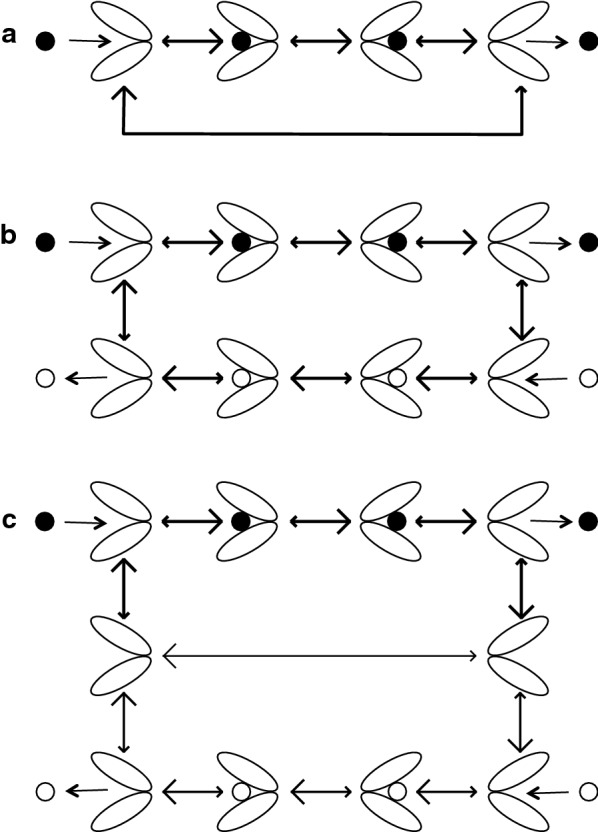
Interpretation of net flux of a single solute, obligatory exchange, and trans-stimulation in terms of a simple carrier model. In each case the concentration of the first solute (filled black circle) is higher on the cis side (left) than on the trans side (right). **a** Net flux of solute from cis to trans is supported by return of the free carrier. **b** If return of the carrier is only possible with a solute bound, there is obligatory exchange, either self-exchange or counter-transport of another solute (circle). **c** Trans-stimulation is a combination of these two effects. Flux of the first solute from cis to trans can be increased if there is more solute (either sort) on the trans side (here the right) provided that increases the rate of return of the carrier—i.e. it increases the rate of conformation changes of the carrier from trans-facing to cis-facing

Since GLUT1 is highly expressed in red blood cells, they have been used as the most convenient system in which to study the kinetics of its transport. There are two prominent features revealed by these studies that must be accommodated in any model. On the one hand the normal net transport of glucose occurs without input of energy from any source other than the concentration gradient, on the other hand downhill movement of one type of sugar can be coupled to uphill movement of another (see Fig. 13), a phenomenon called counter-flow or counter-transport [322–325]. A closely related phenomenon is trans-stimulation, an increase in influx when internal concentration is increased or an increase in efflux when external concentration is increased (see Fig. 13 and, for a quantitative example, Appendix D). In terms of a simple carrier model, the observation of net glucose transport when it is the only substrate implies that both the loaded and unloaded forms of the carrier can change conformation thus altering exposure of the binding site. This allows transport of solute in one direction to occur without transport in the opposite direction, i.e. the transport is not an obligatory exchange. Similarly counter-transport or trans-stimulation imply that the rate constants for the conformation changes when the carrier is loaded are at least comparable to those for the unloaded carrier so that solute on the trans side can assist transport from the cis side by increasing the rate of return of the carrier.

Trans-stimulation can markedly increase influx and efflux of glucose at high glucose concentrations (see Appendix D) and it is therefore very important in studies of the mechanism of transport. However, it has little if any effect on the net flux and it is the net flux that is important for the delivery of glucose for metabolism. The exchanges underlying trans-stimulation are likely to be much more important for large neutral amino acids where several compete for transport by the same carrier (see Sect. 5.5).

The kinetics of the simple carrier model are complex even in the steady-state [325–329]. GLUT1 (SLC2A1) kinetics are complicated further by the added twist that the GLUT1 protein may exist in the membranes as part of a homo-tetramer, each capable of transport, but in a coupled manner such that transport through one affects the transport through the others [322, 330]. Given these and further complexities considered in the next section, it should not be surprising that definitive characterization of glucose transport at the blood–brain barrier remains elusive (see Appendix D).

#### Glucose transport kinetics at the blood–brain barrier

Transport of glucose into and out of the brain is clearly more complex than that into and out of red blood cells. Firstly GLUT1 is needed in both membranes of the endothelial cells of the blood–brain barrier to allow the glucose to enter on one side and leave on the other. However, because the endothelial cells are very thin and correspondingly contain very little glucose, provided that the properties of the transport in the two membranes are similar, it is thought that the transport can still be described, at least qualitatively, as transport across a single barrier [331–333]. Secondly once across the blood–brain barrier, glucose is metabolized at a rate comparable to the rates of influx and efflux across the barrier while in red blood cells transport is much faster than metabolism. Thirdly there is also the technical difficulty that, with the important exception of the study in 1975 by Betz et al. [327], it has not proved possible to manipulate interstitial fluid glucose concentrations during the experiments. In most studies all that has been done is either to measure the extraction of glucose (total or labelled) from blood as described above or to measure the variation in the total amount of glucose present in the parenchyma with time as a function of glucose concentration in plasma. Mason et al. [334] compare the results obtained in many studies performed prior to 1992 but with the surprising omission of reference to studies from Betz’s group. Also in 1992, Gjedde [335] reviewed results obtained for glucose transport in rat and man. Glucose transport into and within the brain has been analyzed and reviewed by Simpson et al. [315], Barros et al. [314] and, more recently, by Patching [336].

In one of the first attempts to establish the mechanism of glucose transport at the blood–brain barrier, Buschiazzo et al. [319] found that 3-*O*-methyl-d-glucose, a non-metabolizable derivative of glucose, competes with glucose for transport, and furthermore that an inward gradient of glucose could drive 3-*O*-methyl-d-glucose uphill out of the brain, i.e. there is counter-transport for GLUT1 at the blood–brain barrier just as in red blood cells. Further evidence that GLUT1 behaves in a similar manner in the two environments was obtained by Betz et al. [327] who found that the rate of glucose influx was increased by increasing the concentration of glucose within the brain, i.e. there is trans-stimulation (see Appendix D).

**Fig. 14:**
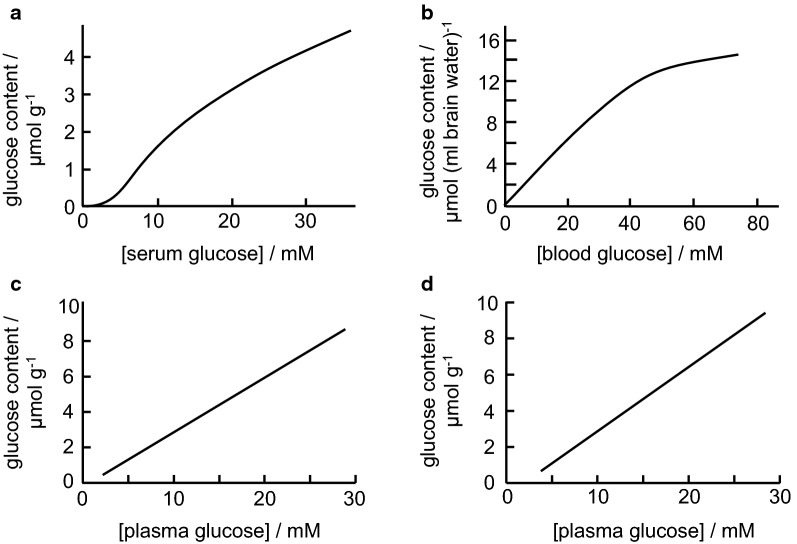
Four studies of brain glucose content versus glucose concentration in blood. In two studies glucose content was measured by chemical assay, **a** in anaesthetized rats by Buschiazzo et al. [319] and **b** in isolated perfused brains from dogs by Betz et al. [327]. In the latter it was assumed that brain water was 0.75 mL g^−1^. In the other two studies glucose content was determined by magnetic resonance spectroscopy, **c** in conscious humans by Gruetter et al. [337] and **d** in lightly anaesthetized rats by Choi et al. [338]. In all studies the glucose content continues to increase with plasma concentration even though it is known that the influx of glucose shows saturation. The explanation is that efflux also saturates and the increase in content must parallel the increase in plasma concentration in order for efflux to increase so that it is equal to influx minus the constant rate of glucose metabolism (see Appendix D)

Buschiazzo et al. [319] and Betz et al. [327] determined the total glucose in the parenchyma for different glucose concentrations in plasma (see Fig. 14). Subsequently NMR has been used to measure glucose content in conscious humans and lightly anaesthetized rats [334, 337–341]. The NMR results for humans and rats confirm under nearly physiological conditions (see Fig. 14) that brain glucose content continues to increase with plasma concentration for plasma concentrations up to at least 30 mM well above a typical resting value, 6 mM. They also confirm that the rates of glucose influx and efflux are respectively larger than and not much smaller than the rate of metabolism. Because influx and efflux substantially exceed the expected efflux via the perivascular route, the net flux across the blood–brain barrier is normally taken to be equal to *CMR*_*glc*_ at steady-state.

In the results reported by Duarte et al. (see Figure 3 in [341]) using rats, following a step change in *c*_*plasma*_ from 4 to 20 mM the brain content of glucose increased from about 0.5 to 4.5 µmol g^−1^ with a half life of about 16 min which indicates a net rate of accumulation of 0.122 µmol g^−1^ min^−1^, i.e. using their value of *CMR*_*glc*_, 0.52 µmol g^−1^ min^−1^, there is an influx of 0.64 µmol g^−1^ min^−1^ which is similar to that reported by Betz et al. in 1974 [302] for the dog.

It has so far not proved possible to analyse glucose efflux directly after injection of glucose into the brain. Any such measurements face major challenges including separating efflux from metabolism and avoiding disturbance of the efflux processes by the injection or infusion. The study by Ball et al. [85] established that during a 5 min, 0.1 µL min^−1^ infusion into the inferior colliculus glucose can move, presumably by a perivascular route, to the adjacent meninges strongly suggesting that as expected there is perivascular efflux of glucose. However, estimating the normal rate of this process to see if the perivascular clearance notably exceeds the 1 µL g^−1^ min^−1^ found in other regions would require measurement of the time course of the appearance of glucose in the meninges after the end of the infusion.^15^

The glucose efflux across the blood–brain barrier can be calculated if the influx and net flux are both known as indicated earlier in this discussion of glucose. Furthermore if it can be assumed that the fluxes are described by the expressions of the form derived from the carrier model, the rate of efflux can be calculated from the measured rates of influx versus the concentrations in plasma and ISF. An example of this using the data from Betz et al. [327] for the isolated perfused dog brain is given in Appendix D and Additional file 1. These data remain the only measurements of glucose influx versus plasma concentration for a range of known concentrations within the brain. Hence the calculated results in Appendix D are the only available results for efflux as a function of both plasma and ISF concentrations.

The fits to the data of Betz et al. [327] (see Additional file 1) indicate that the net flux = *CMR*_*glc*_ for *c*_*plasma*_ = 6 mM is 0.65 µmol g^−1^ min^−1^ with *c*_*isf*_ = 1.2 mM. This value of *CMR*_*glc*_ is close to those expected for rats but about twice that for humans. The fits also predict that glucose consumption, *CMR*_*glc*_, could increase to about 0.9 µmol g^−1^ min^−1^ with *c*_*isf*_ approaching 0 without any change in transport capacity. However, larger increases in glucose consumption are required in order to support nervous activity. Changes in transport capacity are considered in Sect. 6.2.

Both neurons and astrocytes have transporters that will allow uptake of glucose and both can use it as a substrate for energy production. The proportions of glucose metabolism that occur in astrocytes and neurons remain controversial [315, 342–346] (see next section).

### Lactate

When at rest and even more during nervous activity, there is net production of lactate within the brain parenchyma and thus there must be means for its efflux. Clearance of lactate from the brain has recently been reviewed in some detail [146] (see also footnote 26 in [4]). In brief lactic acid is transported across the blood–brain barrier by passive transport mediated by MCT1 (SLC16A1) present in both luminal and abluminal membranes. Lactate both enters and leaves the brain by this route. Lactate is generated within the brain by partial metabolism of glucose and by metabolism of glutamate [347, 348]). Under resting conditions when lactate concentrations are low, the clearance, *CL* = *PS* ~ 60–100 µL g^−1^ min^−1^ [349–352], far exceeds the expected clearance, ~ 1 µL g^−1^ min^−1^, by a strictly perivascular route.

It is often said that transport of lactate across the blood–brain barrier is slow (see e.g. Pardridge’s account [189]). But these statements refer to the amounts transported not the permeability. The lactate clearance (= *PS* product) calculated for low concentrations from the kinetic constants that Pardridge presents, *K*_*t*_ = 1.8 mM and *T*_*max*_ = 91 nmol g^−1^ min^−1^, is 50 µL g^−1^ min^−1^, close to that stated above. Quistorff et al. [353] and Boumezbeur et al. [352] have emphasized that lactate from the periphery can be an important source of energy in the brain during heavy exercise.

**Fig. 15:**
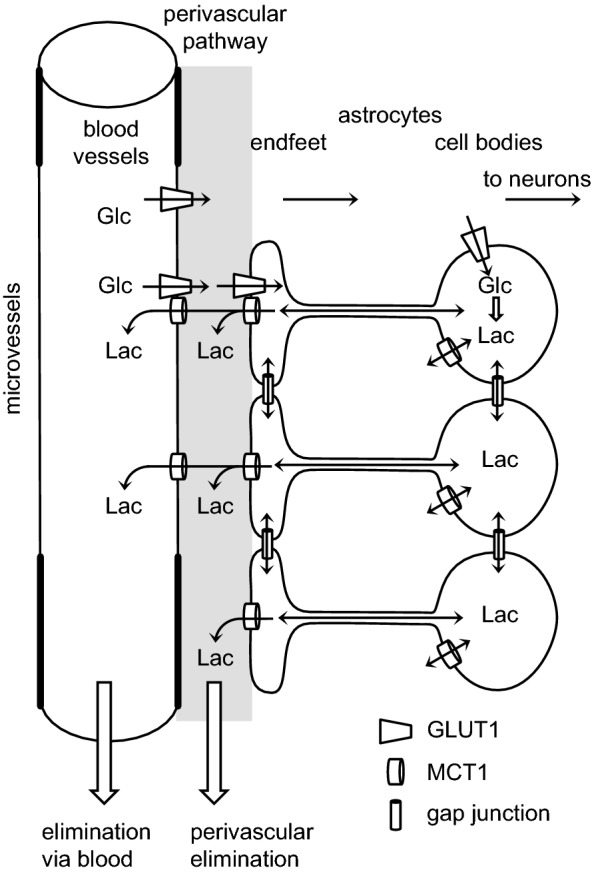
Lactate removal from the brain. Lactate produced within the brain can be effluxed via the blood–brain barrier or via perivascular routes. It may reach the latter locally near the site of its production or at more distant sites having been transferred between astrocytes via gap junctions (Diagram modified from Figure 7c in Gandhi et al. [358])

There is clear evidence that during periods of increased neural activity the blood–brain barrier is not the only route of lactate removal from the sites of activity [354–357]. This may be particularly important in circumstances where the lactate concentration is also increased in the rest of the body, e.g. as a result of physical exercise. Under these circumstances the net transport across the blood–brain barrier is likely to be inwards [352, 353]. Other routes for efflux cannot be just perivascular transport as seen with inulin because that isn’t fast enough. One suggested explanation is perivascular transport augmented by transfer between astrocyte endfeet via gap junctions. This can lead to movement of lactate from sites of activity either to inactive regions or to perivascular spaces of larger blood vessels [356–358] (see Fig. 15). Much of the lactate removed from the parenchyma via perivascular transport is likely to be removed from the brain along with CSF, though a proportion reaches lymph, possibly via the meninges, without first mixing with CSF. Lactate in CSF that leaves via the cribriform plate is delivered to the nasal mucosa from which it may return to blood either indirectly via lymph or directly by crossing peripheral capillary walls [85, 120, 125].^16^

It remains puzzling why so much of the lactate produced within the brain during nerve activity appears to be removed rather than serving as fuel for oxidation in neurons as proposed in the astrocyte neuron lactate shuttle (ANLS) hypothesis (G. A. Dienel, personal communication). However, at least according to Dienel [345] the available evidence is that the oxygen consumption does not increase sufficiently during nerve activity for shuttling of lactate from astrocytes to neurons and further oxidative metabolism of lactate in neurons to be an important mechanism. Furthermore using expression of a genetically encoded NAD sensor that can be monitored in real time with cellular resolution, Diaz-Garcia et al. [346] have found in mice that nervous activity induces neural production rather than consumption of lactate. For an alternative view see e.g. [344].

### Amino acids

In order to put the importance of efflux of amino acids from brain parenchyma into context, it is necessary to consider not just the fluxes and transporters but also the need for fluxes.

**Fig. 16:**
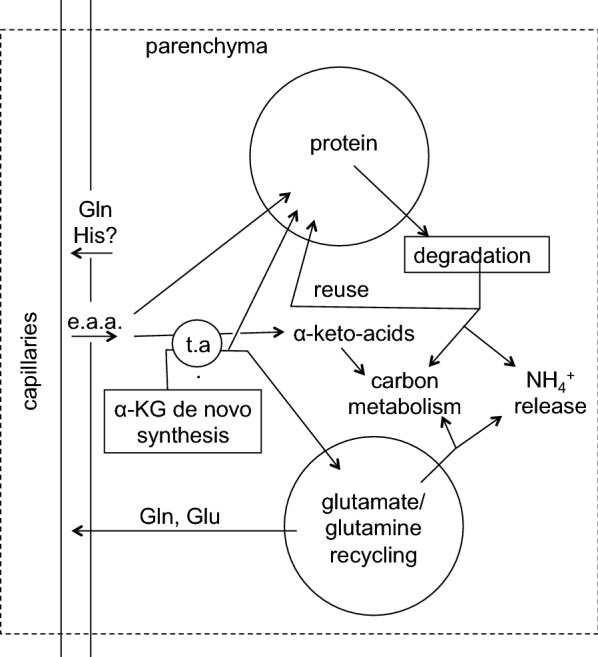
Simplified overview of fates of amino acids in the brain parenchyma. Essential amino acids enter and leave the parenchyma across the blood–brain barrier. Non-essential amino-acids, e.g. glutamate (Glu), glutamine (Gln), and GABA can be synthesized within the brain. The amino groups for the synthesis are supplied either by transamination as shown for glutamate or to some extent [359] by incorporation of NH_4_^+^ by glutamate dehydrogenase. The latter route is believed to be minor [359, 641]. NH_4_^+^ is added to form the amide group of glutamine by glutamine synthetase (see Fig. 17). Within the parenchyma amino acids are used for synthesis of proteins and (not shown) formation of other nitrogen containing compounds, e.g. nucleotides. New amino acids must be supplied to replace those lost by metabolism. In the brain, input of amino acids is also required to provide amino groups to replace glutamate lost from the pool of amino acids involved in glutaminergic (and GABAergic, not shown) neurotransmission (see Fig. 17). *α-KG* α-ketoglutarate, *e.a.a* essential amino acids, *t.a* transaminase

**Fig. 17:**
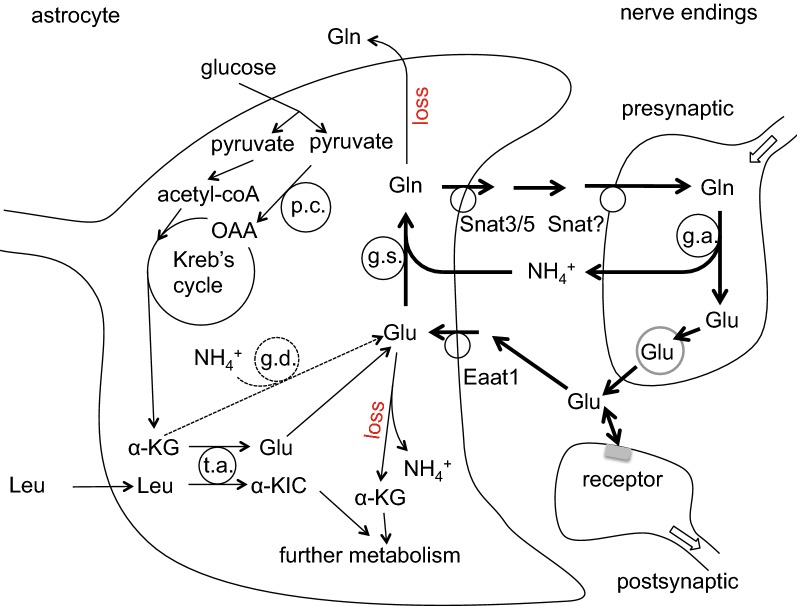
The glutamate/glutamine cycle shown in bold with indication of some of the losses and of replenishment of glutamate by denovo synthesis. Glutamate (Glu) in the presynaptic neuron is packaged into vesicles and released into the ISF during neurotransmission. Most of the glutamate is taken up into the astrocytes by the transporter Eaat1 (glast, Slc1a3) where it is converted to glutamine (Gln) by addition of an NH_4_^+^ by the enzyme glutamine synthase (g.s) [642, 643]. The glutamine is transported into the ISF by Snat3 and/or Snat5 (Slc38a3 and Slc38a5) from which it is taken up into the presynaptic terminals again by a transporter that may be a Snat. The glutamate is then regenerated by glutaminase (g.a). This cycle represents a large turnover of the amide group at the end of the side chain in glutamine, estimated to be 55% of the *CMR*_*glc*_ (cerebral metabolic rate of glucose, see Sect. 5.3) for the entire brain in rats amounting to 490 nmol g^−1^ min^−1^ (estimated value in humans 280 nmol g^−1^ min^−1^) [644] (G. A. Dienel, personal communication). However the requirement for NH_4_^+^ consumed in the conversion of glutamate to glutamine within the astrocytes is balanced by an equal release of NH_4_^+^ in the reverse conversion in neurons. Whether diffusion of NH_4_^+^ itself is adequate to transfer the nitrogen from neurons to astrocytes as shown or some other form of N carrier is required remains controversial [385, 641, 645]. Regardless, if there were no losses of glutamine or glutamate from the cycle, there would be no need for any fluxes of amino acids into or out of the parenchyma to support glutaminergic neurotransmission. However, there are losses of glutamate and glutamine from the cycle [347, 646, 647]. At least in rodents, such losses are made good by de novo synthesis of glutamate in the astrocytes. Estimates of the total rate of loss and of de novo synthesis are around 11% of *CMR*_*glc*_ ([648, 649] (G. A. Dienel, personal communication), i.e. about 0.11 × 0.9 µmol g^−1^ min^−1^ ≅ 100 nmol g^−1^ min^−1^. The carbon skeletons for the de novo synthesis are derived ultimately from glucose. Glucose is metabolized to two molecules of pyruvate one of which is carboxylated by pyruvate carboxylase (p.c) (thought to be present within the brain only in astrocytes) to form oxaloacetic acid (OAA) a component of the citric acid cycle. Addition of acetyl-CoA from the second pyruvate then forms citrate, which is decarboxylated to form α-ketoglutarate (α-KG). Glutamate is then formed either **a** by transamination (t.a) of α-ketoglutarate using leucine or other amino acids as source (see e.g. [383, 641, 645, 650], or **b** by addition of NH_4_^+^ [366] catalyzed by glutamate dehydrogenase (g.d). The latter is believed to be a minor pathway [359, 366, 641]. The source of the amino groups for transamination is considered further in Sect. 5.5.3 and Fig. 18. Data for the pathways involved in glutamate synthesis are much less extensive for human than for rat. Rothman and colleagues [651, 652] have argued that the α-ketoglutarate is synthesized in astrocytes based on measurements of incorporation of ^13^C (see [653, 654]). However, the failure to find a key transaminase in human astrocytes by immunohistochemistry [655, 656] has cast some doubt on astrocytes being the major site for the conversion from α-ketoglutarate to glutamate. For recent reviews of glutamate synthesis see [386, 641, 645]

Amino acids are required within the brain for protein synthesis (see Fig. 16) and for maintenance of pools of neurotransmitters, in particular glutamate and GABA (see Fig. 17). Amino acids are also needed for synthesis of many other substances, e.g. nucleosides, but when considering overall balance this demand has usually been ignored as being relatively minor and it will not be considered further here (compare [359]). The required amino acids must either be synthesized inside the brain or enter from outside primarily across the blood–brain barrier.

The need for amino acid input is different from the need for glucose input. Glucose is the basic fuel consumed in metabolism and must be supplied continually in large quantities. Amino acids are needed to allow the maintenance of cell structure and composition. But, the N containing constituents of the cells either are not consumed during metabolism or if they are they are partly replaced internally. The balance between influx and efflux across the blood–brain barrier need only provide sufficient amounts of amino acids to top up losses. Any metabolic losses that do occur will either be by efflux from the brain or by generation of NH_4_^+^ and carbon compounds. The latter become part of the carbon metabolism of cells. Possible fates of the NH_4_^+^ include: diffusion across the blood–brain barrier; reaction with glutamate to form glutamine, which is then exported from the parenchyma; and use in amino acid synthesis [359, 360]. Glutamate synthesis is considered further in Sect. 5.5.5.

For each amino acid at steady-state, its net fluxes across the blood–brain barrier and via perivascular routes and its net rate of synthesis must add to zero so that the concentrations in the brain parenchyma can remain constant. However, there are major complications in applying this principle to the interpretation of data: there are more than 20 different amino-acids, inter-conversions between them by transamination are common, and they compete with each other for the many amino acid transporters. Indeed the major application of this principle comes when considering overall N balance.

Allowing the fluxes that are required (see Sect. 5.5.1) while maintaining ISF concentrations of all amino acids except glutamine well below those in plasma (see Sect. 5.5.2) is a major challenge and it is not yet certain how the available transporters (see Sect. 5.5.4) achieve these objectives.

#### Requirements for amino acid fluxes (and NH_4_^+^)

While it is clear that there are losses of essential amino acids from brain parenchyma and thus that some influx of amino acids must occur, it is difficult to obtain a quantitative estimate of the influx required. Using radiolabelled amino acids in rats, Dunlop et al. [361–363] found a turnover rate for the protein content of rat brains to be about 0.6% h^−1^. Using a protein content of about 100 mg for each gram of brain and the molecular weight of an average amino acid, perhaps 125 Da, that corresponds to a rate of incorporation of amino acids of about 80 nmol g^−1^ min^−1^. Similarly amino groups required for de novo synthesis of glutamate amount to about 100 nmol g^−1^ min^−1^ (see legend to Fig. 17).

Many of the amino acids needed for protein synthesis are supplied either by de novo synthesis (which, however, still requires some source of amino groups, see Fig. 16) or by recycling those released during protein breakdown, which averaged over enough time must be occurring at the same rate as synthesis. Furthermore it may be possible to reuse some of the NH_4_^+^ lost from the glutamate/glutamine cycle in the de novo synthesis of glutamate. Thus the sum of the estimates above, 180 nmol g^−1^ min^−1^, is likely to exceed the actual requirement for amino-acid input.

Because the brain parenchyma must be in N balance and there must be net inputs of essential amino acids, there must also be a route or routes for N removal. As the brain normally doesn’t produce urea as a means of disposing of NH_4_^+^ [364–366], the two main routes for exit to be considered are efflux of NH_4_^+^ and efflux of glutamine. Fluxes of NH_4_^+^ are easily demonstrated to occur in both directions across the blood–brain barrier and are almost certainly by diffusion across the membranes of NH_3_ combined with transport either of H^+^ in the same direction or, more likely, of HCO_3_^−^ in the opposite direction [4, 359, 367]. Because concentrations of NH_4_^+^ in brain, 150–300 µM, and CSF, 100–300 µM, normally exceed those in arterial plasma, 50–250 µM [359], it is likely that there is some net NH_4_^+^ efflux. However, an arterio-venous difference in NH_4_^+^ concentration and thus its net transport have only been demonstrated in the brain when plasma NH_4_^+^ concentration is raised as in hepatic insufficiency [359, 368]. There is then net NH_4_^+^ entry, rapid incorporation of the NH_4_^+^ into glutamine by reaction with glutamate [359], and efflux of the resultant glutamine. Glutamine efflux is considered further in Sect. 5.5.4.

Lee et al. [360] made the interesting suggestion that much of the NH_4_^+^ that moves from brain microvascular endothelial cells to plasma is produced within the endothelial cells by glutaminase acting on glutamine. However, that taken alone would suggest that there should also be a substantial efflux of glutamate, which has not been observed. Alternatively the NH_4_^+^ effluxed may derive from metabolism of both glutamine and glutamate. This is considered further in Sect. 5.5.4.

#### Concentrations of amino acids in CSF and ISF

Values of amino acid concentrations measured in blood plasma, CSF and ISF are summarized in Table 3. There is agreement in all studies that, with the exception of glutamine, the concentrations of all other amino acids in CSF and ISF are substantially less than those in plasma. This could arise if the rates of consumption were to reduce the concentrations greatly or if there were active transport of amino acids from brain fluids to blood. Whether or not there is a substantial difference in amino acid concentrations between CSF and ISF is less clear.^17^

**Table 3:** Amino acid concentrations in plasma, CSF and ISF

	Plasma concentration/µM						CSF concentration/µM								ISF concentration/µM				
	Human^1^	Rat^2^	Human^3^	Rabbit^4^	Rat^5^	Mice^6^	Human^1^	Human^7^	Rat^2, c^	Rat^1, 2^	Human^3^	Rabbit^4^	Rat^5^	Mice^6^	Rabbit^4^	Rabbit^8^	Rabbit^9^	Rat^5^	Mice^6^
Gln		619	641	868	834	598			863	780	552	583	524	517	250	547	159	193	80
Asn			112	55			Trace				14	16			3	7.8	4.3		1.4
Ala	330		489a	382	430	408	27	18.6			34a	39	85.4	57	15	19	15	7.7	9.3
Ser	149	196	140	263	247	109	28.9	30.4	79	80	30	116	62.3	37	34			24.9	9.8
Gly	249	221	283			246	1.3	6.4	20	15	6			34					7.8
Pro	212					60	Trace	0.6						4.2					1.8
Thr	142		166			113	28.5	27.7			36			22					3.5
Val	222	430	309	191		173	14	16.1	78	47	20	14		9.4	13	12.5	7.4		2.9
Leu	109	432	155	81		127	13.1	11.9	66	42	15	7		6.6	9				1.5
Ile	61		77	101			5.2	4.7			6	7			9				
Tyr	70		73	43		54	7.3	8.1			10	7		7.2	5				0.7
His	85		80			59	10.8	12.0			12			8.9					1.2
Lys	158	290	171	137		321	19.7	22.0	120	87	21	8		46	19				10
Arg	80	94	81		227	103	17	18.6	55	46	22		32.5	18				6.9	3.2
Glu	83		61	56	159.6	37	7.2	8.7			26	10	11.4	21	4	4.3	3.4	2.9	4.1
Asp	7				33.8		2.4	1.5					5.8			0.6	0.5	1.7	
Phe	71		64	45			8.3	8.2			10	7			6				
Met	41		28	30			2.8	2.6			3				3				
Trp	62																		

Concentrations measured in CSF are, with the exception of glutamine always substantially less, than the concentrations in plasma. Concentrations in ISF are measured by microdialysis with extrapolation to zero flow (see text). If these are correct, ISF concentrations are substantially lower than those in CSF. *c* cisternal, *l* lumbar

^1^Plum et al. [576]

^2^Franklin et al. [577]

^3^McGale et al. [578]

^4^Hamberger et al. [579]

^5^Lerma et al. [580]

^6^Dolgodilina et al. [581]

^7^Table 8.15 in Davson and Segal [56]

^8^Jacobson et al. [582] microdialysis by concentration profile

^9^Jacobson et al. [582] microdialysis by recovery of samples

#### The relative importance of perivascular supply and removal for amino acid turnover in ISF

Excluding glutamine, concentrations of each of the amino acids in CSF and ISF are usually < 1/5th of those in plasma (see below) and in total < 1 mM. With a perivascular clearance of 1 µL g^−1^ min^−1^, and an amino acid concentration at the high end of the observed range, 100 µM, the rate of loss or gain of any particular amino acid by the perivascular route is expected to be of the order of 0.1 nmol g^−1^ min^−1^ or less, which is likely to be negligible. Amino acid loss from the brain by outflow of CSF at 0.25 µL g^−1^ min^−1^ (500 mL day^−1^ for a 1400 g brain) at 100 µM would be 0.025 nmol g^−1^ min^−1^ which again is likely to be negligible.

#### Observed fluxes of amino acids

Quantitative measurements of fluxes of amino acids have been either of influx or net flux. Influx is measured by adding a tracer to the blood perfusing the brain and measuring the amount that enters the brain over a short period. Net flux of an amino acid is calculated as

10 $${\text{net flux }} = \left( {{\text{A}} - {\text{V difference}}} \right) \times {\text{blood flow}}$$

by using measurements of the blood flow and the A − V difference equal to the difference between the concentrations in arterial blood entering and venous blood leaving the brain. Direct measurements of efflux have proved difficult. In practice efflux into the blood has been calculated as the difference between influx and net flux from the blood.

*Influx of amino acids* into brain parenchyma across the blood–brain barrier has been studied in rats. In a highly influential early study, rates were compared to that for water using ^14^C-labeled amino acids and ^3^HOH added together as a single bolus arterial injection. The results were reported as the brain uptake index (BUI), defined as a ratio of ratios ((uptake of 14C-aa)/[14C-aa])/((uptake of ^3^HOH)/[^3^HOH]) [300]. When added one at a time, the influxes of the amino acids varied greatly, with BUI for phenylalanine or leucine found to be more than 50% (i.e. each enters about half as easily as water) while at the other extreme influxes of proline, glutamate, asparagine and glycine were below the background limit of detection by the technique, BUI < ~ 3%. Influx of each of the essential amino acids (those not able to be formed within the brain) was easily measurable.

All of the influxes that were clearly above baseline were inhibited when the radiolabeled amino acids were added using serum rather than a simple buffer suggesting competition for transport with the amino acids present in serum. Competition was investigated further and confirmed by measuring uptake of tracer in the presence of an excess of individual unlabelled amino acids [300].^18^ Quantitative estimates of the influxes of various amino acids in rats when plasma concentrations of tracers were held constant by controlled infusions [369] or during perfusion of isolated brains [43, 370, 371] have confirmed the pattern seen using BUI measurements [300, 372] (see Table 4).

**Table 4:** Influx and net flux of amino acids in the indicated species

	Rat^1^	Rat^2^	Rat^3^	Rat^4^	Rat^5^	Rat^6^	Dog^7^	Dog^7^	Cat^8^	Sheep^9^	Human^10^	Human^11^	Human^12^	Human^13^	Human^14^
	Influx*	Influx	Influx	Influx	Influx	Net	Net*	Net*	Net	Net	Net	Net	Net	Net	Net
Phenylalanine	6.5	7	8		13.2	−* 0.6*	0.12	0.01			2.2		0.06	0.3	− 0.5
Leucine	6.2		15		14.5	*9.7*	1.56	1.28		1.9	6	5.2	3.2	2.2	0.6
Isoleucine	1.8				4	*5.7*	0.78	0.46		1.5	3.1	1.2	1.4	0.7	0.3
Valine	1.3				1.8	*2.9*	1.26	0.37		2.7	11.0		1.7	0.8	− 1.7
Tyrosine	5.3	7			4.1	− *1.1*	0	0.06			1.6		0.1	− 0.1	− 0.1
Methionine	1.6				1.7	− *1.1*	0	− 0.12			1.0		0.6	0.2	0.2
Tryptophan						− *1.1*									
Histidine	2.5				2.5	− *1.7*	− 0.23	− 0.7					0.7	− 0.3	− 1.3
threonine	1.2				0.8	− *3.4*	0.62	− 0.1			5.0		2.6	0.1	
Arginine	1					− *7.4*	− 0.2	− 0.05				2	0.7	− 0.5	− 0.1
Lysine	6.2		9			− *1.1*	0.58	− 1.6					2.5	− 0.3	− 0.9
Glutamate						*1.7*	− 0.24	− 0.18	− 0.05				1.4	− 0.5	− 6
Glutamine^a^				11.6	1	− *15.4*	− 6.6	− 4.3	− 1.94	− 7.4			11.0	− 20	− 20.4
nerv. stim.^b^ glutamate									− 1.26						
nerv. stim.^b^ glutamine									− 6.69						

Values are stated in nmol g^−1^ min^−1^. Influxes have been measured only in rats. The only available measurements of net fluxes in rats, shown in the italicized column, failed to reach statistical significance

* Data used by Pardridge [379] in his comparison of influxes and net fluxes

^a^In some columns the values are for glutamine + asparagine as the assay used detected both

^b^Release measured during nervous stimulation

^1^Banos et al. [369]

^2^Hawkins et al. [583]

^3^Mans et al. [584]

^4^Ennis et al. [415]

^5^Smith and Stoll [43]

^6^Brosnan et al. [378]

^7^Betz and Gilboe [365]

^8^Abdul-Ghani et al. [394]

^9^Pell and Bergman [585]

^10^Felig et al. [586]

^11^Lying-Tunell et al. [587]

^12^Eriksson et al. [588]

^13^Grill et al. [589]

^14^Strauss et al. [590]

From the patterns of competition between amino acids for influx across the blood–brain barrier it appeared that there were four separate systems of transport (see e.g. [43, 44]).

- System L primarily for neutral amino acids, which can be inhibited by 2-aminobicyclo-(2,2,1)-heptane-2-carboxylic acid (BCH);
- System ASC primarily for neutral amino acids, which is not inhibited by BCH;
- System y^+^ (sometimes called system Lys^+^) primarily for basic amino acids;
- System N primarily for the nitrogen-rich amino acids glutamine, histidine and asparagine.

A number of amino acids fit into more than one of these groups. Most of the amino acids with large BUI values are substrates for system L.

Studies with isolated brain microvessels, which provide access to the abluminal membranes of the endothelial cells, identified two more systems.

- A Na^+^-linked transport system for small neutral amino acids (system A, with identifying substrate *N*-methyl-a-aminoisobutyric acid, MeAIB) [373].
- Another system for glutamate [374].

The ability to prepare vesicles enriched in membranes from either the luminal or abluminal membranes of the endothelial cells [375] allowed localization of transport activities to the separate membranes with the generalization (since revised, see Sect. 5.5.6) that transporters in the luminal membrane are not Na^+^-linked and hence bidirectional while those in the abluminal membrane are Na^+^-linked favouring transport from ISF into the endothelial cells [44]. There are now known to be many more types of transporter present at the blood–brain barrier than initially suggested by identification of these systems (see Sect. 5.5.6).

*Large rates of efflux* of amino acids from CSF to blood were detected in cats [376] and rabbits [377] undergoing ventriculo-cisternal or ventriculo-cortical subarachnoid space perfusions. However, it was not possible in these studies to determine how much of the efflux was going via the choroid plexuses and how much via the parenchyma and the blood–brain barrier. Evidence that the latter route is important derives from the observation that transfer was much more rapid in ventriculo-subarachnoid infusion than in ventriculo-cisternal infusion. Both types of perfusion expose the infused fluid to the choroid plexuses, but in the former a much larger surface area of parenchyma is exposed to the fluid [376].

*The net flux* into a region can be calculated if the blood flow to that region and the concentrations of the solute in arterial blood and the venous outflow can be measured (see Sect. 5.5.6). (Equating net flux out of blood with net flux into the brain ignores possible metabolism within the endothelial cells, see the end of Sect. 5.5.6). Net flux measurements have been attempted using rats [378], but all except one of the A − V differences were not statistically significant. The rest of the net flux data in Table 4 are for larger species.

Pardridge [379] compared the influx data for rats obtained by Banos et al. [369] with the net flux data for dogs obtained by Betz et al. [365] (see Table 4) and noted that the net fluxes are much smaller than the unidirectional influxes. With the assumption that the fluxes are similar in various species, this comparison implies that there must be large effluxes, comparable in size to the influxes. Measurements of net fluxes in dogs, sheep, and humans have produced data broadly comparable with each other (see Table 4) favouring the assumption that when expressed per gram of tissue the fluxes are the same in all species.^19^

At present there are strong indications that the net flux of glutamine is outwards. This was seen in five out of six studies. There is also indication that the combined net flux of the branched chain amino acids, leucine + isoleucine + valine, is inwards. This was seen in six out of seven studies. But as described in the next section there is no evidence for a sufficiently large inwards net flux of neutral amino acids to provide for all of the transamination invoked in the explanations of glutamate turnover, at least in rats.

#### Observed fluxes of neutral amino acids compared with their requirement in glutamate synthesis

A major difficulty is revealed by comparison of the small net fluxes for the large, essential neutral amino acids and the large provision of these amino acids required for transamination to convert α-ketoglutarate into glutamate (see Figs. 16 and 17). For this requirement to be satisfied by influx across the blood–brain barrier of leucine, isoleucine and valine, their combined net influx would need to be > 100 nmol min^−1^ g^−1^ (see Sect. 5.5.1). For a cerebral blood flow of 0.57 mL min^−1^ g^−1^ (see e.g. Sect. 5.3) that would correspond to an A − V difference > 175 µM. Given that the total of the arterial plasma concentrations for these amino acids is only 392 µM (see Table 3), this A − V difference and hence net rate of transport should have been well above the “noise” in all of the studies, even that in rats (see Table 4).

If, as indicated by all available studies, sufficient net inward flux of amino acids does not in fact exist, the amino groups for synthesis of glutamate in the astrocytes must be obtained from sources within the brain. Independent evidence that such a source is available comes from studies comparing isotope dilution in the brain when plasma leucine was labeled with ^13^C or ^15^N. 62% of the N in brain leucine was derived from reverse transamination [380–383].

**Fig. 18:**
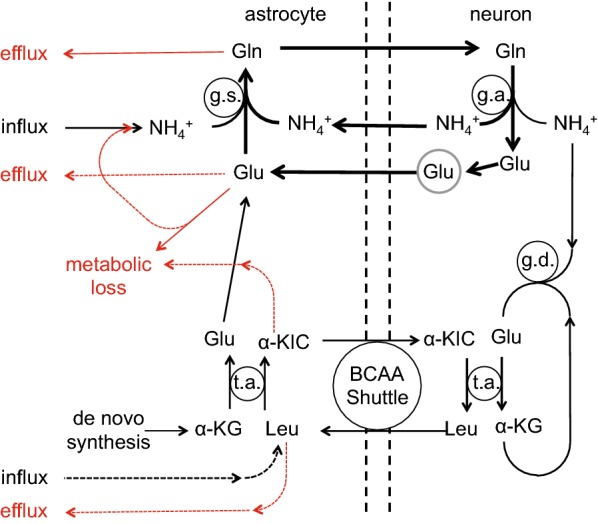
The branched chain amino acid shuttle for provision of branched chain amino acids (BCAA) in the astrocytes to allow de novo synthesis of glutamate. Leucine (Leu) is used as example of a BCAA. *α-KG* α-ketoglutarate, *α-KIC* α-ketoisocaproic acid, *Gln* glutamine, *Glu* glutamate, *g.a* glutaminase, *g.d* glutamate dehydrogenase, *g.s* glutamine synthetase, *t.a* transaminase. Losses of Gln, primarily by efflux, and of Glc, primarily by catabolism are replaced by de novo synthesis of α-KG in astrocytes and transamination using Leu producing α-KIC. Leu is regenerated from α-KIC in the neuron by transamination from Glu producing α-KG. The Glu is in turn regenerated from the α-KG and NH_4_^+^ by gdh. Loss of N via efflux of Gln, Glu, and Leu is made good by net inward flux of Leu and NH_4_^+^. The BCAA shuttle greatly reduces the need for net inward flux of Leu as this is only required to make good the metabolic loss of α-KIC (Based on Figure 1 in Hutson [384])

One detailed suggestion (see Fig. 18) is that loss of the branched chain α-ketoacids (BCKA), e.g. α-ketoisocaproate, generated in the transamination in the astrocytes is prevented by using a branched chain amino acid (BCAA) shuttle ([382, 384], reviewed in [385]). In this scheme instead of being further metabolized within the astrocytes as shown in Fig. 17, the BCKA are transferred to neurons where the branched chain amino acids (BCAA), e.g. leucine, can be regenerated by transamination from glutamate producing α-ketoglutarate. The leucine is then exported back to the astrocytes while the glutamate within the neuron is regenerated by glutamate dehydrogenase from NH_4_^+^ and the α-ketoglutarate [384, 386]. In this scheme NH_4_^+^ is taken from the neuron where it is released from glutamine and will be at relatively high concentration. This is shifted to the astrocyte by the BCAA shuttle where it can be combined with new α-ketoglutarate to complete the de novo synthesis of glutamate. This scheme greatly reduces the need for net flux of BCAA across the blood–brain barrier.

#### Amino acid transporters at the blood–brain barrier

**Fig. 19:**
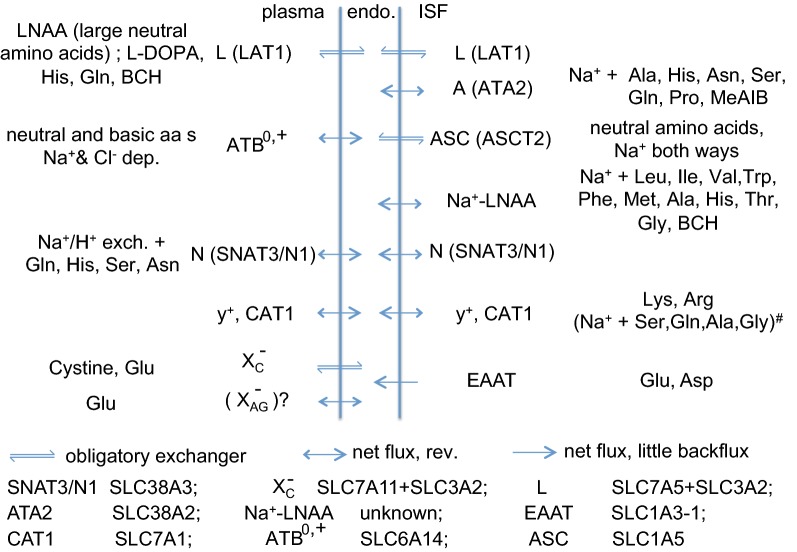
Amino acid transporters thought to exist at the blood–brain barrier. Based on Nalecz [200]; Broer [393]; Mann et al. [520]; O’Kane et al. [657]; and Hawkins et al. [44]. ^#^See [44, 398] but contrast [399, 400]

The transporters currently thought to be involved in amino acid transport across the blood–brain barrier are indicated in Fig. 19. These will be discussed below according to the categories of amino acids transported.

*Anionic amino acids*, in particular glutamate, are transported by EAATs 1, 2 and/or 3 (coded by SLC1A3, 2, 1 respectively) which are found only in the abluminal membrane of the endothelial cells [387]. These EAATs mediate co-transport of the anionic amino acid together with 3 Na^+^ ions and 1 H^+^ ion followed by return transport of 1 K^+^ ion [388–390]. Because the electrochemical gradient for Na^+^ is directed from ISF into the endothelial cells and 3 Na^+^ ions are transported, this coupling renders the amino acid transport effectively unidirectional into the cells. Glutamate is also produced within the endothelial cells from breakdown of glutamine mediated by glutaminase [360]. Glutamate in the endothelial cells can then either be metabolized releasing NH_4_^+^, as argued by Helms and colleagues [391, 392], or be transported to blood plasma by a transporter other than an EAAT. Glutamate metabolism within endothelial cells is analogous to the extensive metabolism known to occur within gut epithelial cells (see e.g. [393]). Glutamate transport from brain endothelial cells to plasma has been demonstrated after sensory stimulation in vivo, which increases glutamate production [394]. This transport is likely to be via the glutamate/cystine exchanger, X_c_^−^ (SLC7A11 + SLC3A2), [200, 395]), though there is also evidence for a transporter, yet to be identified, that functions in the absence of cystine [396].

*Cationic amino acids* such as arginine and lysine are transported by CAT-1 (SLC7A1), which is known to exist in the abluminal membrane of the endothelial cells. Transport of these amino acids across the luminal membrane is less well-characterized but may be also via CAT-1 or possibly ATB^0,+^ (SLCA14). Transport of cationic amino acids by CAT-1 can involve exchange of one amino acid for another (trans-stimulation see Sect. 5.3.1), but this is not essential [397]. There may be at least one more transporter for cationic amino acids at the abluminal membrane (but see [398]). Hawkins et al. [44] reported that cationic amino-acid transport across both membranes can be inhibited by a number of neutral amino acids in the presence of Na^+^. CAT-1 is thought not to be so affected [397, 399, 400]. The additional transporter may be y^+^L [4F2hc (SLC3A2) + either y^+^LAT2 (SLC7A6) or y^+^LAT1 (SLC7A7)] [399, 400].

*Neutral amino acids* are transported by several systems as indicated in Fig. 19.

- System L, primarily the heterodimer 4F2hc/Lat1 (Slc3a2 + Slc7a5) which is present in both membranes and functions independently of Na^+^;
- System A, primarily ATA2 (Slc38a2) in the abluminal membrane which because it is a Na^+^-linked transporter is biased towards transport from ISF into the endothelial cells;
- ASC, primarily ASCT2 (Slc1a5), an obligatory exchanger that requires the presence of Na^+^-but is not driven by the Na^+^ gradient;
- System Na^+^-LNAA a Na^+^-linked system whose molecular basis is still unknown;
- ATB^0,+^ (SLC6A14) which allows net fluxes without exchange;
- And possibly the y^+^L transporter [4F2hc (SLC3A2) + either y^+^LAT2 (SLC7A6) or y^+^LAT1 (SLC7A7)].

The large influxes of neutral amino acids from blood-to-brain seen in the early work and ascribed to system L have subsequently been shown to be mediated by 4F2hc/Lat1 [401–403]. The discovery that not only can this system mediate exchanges of amino acids [404, 405] but the exchange is obligatory [406–408] has far reaching consequences for amino acid transport at the blood–brain barrier [409]. It provides an important part of the explanation for how it is that there are large unidirectional fluxes (influx and efflux) but only small net fluxes. In order for system L to mediate a net inward flux of one amino acid, it must have net outward flux of another. An exchanger of neutral solutes, like system L, tends to equilibrate the concentration ratios for all of its substrates. Thus predicting the flux of any one of the amino acids across a membrane requires knowledge of the concentrations of all of the substrates on *both* sides of the membrane.^20^ Consumption of any system L substrate within the parenchyma will by reducing its ISF concentration tend to lead to net inward flux of that substrate and net outward flux of others. Similarly production of any system L substrate will tend to lead to its net outward flux together with net inward flux of others.

The function of 4F2hc/Lat1 (Slc3a2/Slc7a5), the principal component of system L, was explored in mice by Tarlungeanu et al. [410]. They compared the concentrations of amino acids in brain (amount per unit weight of brain) between a conditional Slc7a5 knockout [411] and normal controls. In adult mice they found that the levels of methionine, leucine and isoleucine in the knockouts were about 0.66 times the levels in normals, i.e. a reduction of about 35%. This suggests that there is normally a net inward flux of these amino acids via 4F2hc/Lat1 but that there are other routes at least as important. By contrast levels of phenylalanine, proline, glycine, threonine, and serine in the knockouts were about 1.3 times higher than in normals, i.e. an increase of about 30%. This suggests that for these amino acids there is normally a net outward flux via Lat1 but that there are other important routes for their elimination. More dramatically with histidine the level in knockouts was sevenfold higher, a 600% increase compared to normals. This suggests that 4F2hc/Lat1 is normally the main route for eliminating histidine from the parenchyma and that a net inward flux of histidine occurs by some route other than 4F2hc/Lat1. However, it is important to note that while these results show that 4F2hc/Lat1 is very important for the fluxes of histidine, they do not in themselves show that histidine efflux is a large fraction of the total efflux carried by 4F2hc/Lat1. Further evidence for exchanges involving histidine have been obtained using 4F2hc/Lat1 expressed in proteoliposomes. High concentrations of cysteine inside the vesicles can allow or drive influx of histidine and high concentrations of many amino acids outside of the vesicles can allow or drive efflux of histidine [412].

It has been tempting to propose that the combined net flux of neutral amino acids, inward or outward, is determined by their fluxes via systems other than system L and by their synthesis and breakdown in the parenchyma. System L is, however, still important, because it is the combined action of system L with the other transporters that determines which of the neutral amino acids move inwards and which outwards. A coherent overall account of the transport of neutral amino acids across the blood–brain barrier is still awaited.

With regard to glutamine, which is synthesized within the parenchyma, it has been tempting to propose that a substantial part of its efflux occurs via a system L mediated exchange for the essential large neutral amino acids such as leucine, isoleucine, valine and phenylalanine entering the parenchyma. Indeed such exchanges can be observed with isolated microvessels under experimental conditions [413, 414]. However, there is no evidence for this effect under conditions that exist in vivo.

The observation that there is a net efflux of glutamine is especially important because it is present at high concentration in plasma and ISF and it is the obvious sink for excess NH_4_^+^ in the brain. Glutamine is a substrate not only of 4F2hc/LAT1 (system L) as outlined above but also of Snat3 (SLC38A3) (system N), ATA2 (SLC38A2) (system A), and CAT (SLC7A1) (system y^+^) [200]. Of these the principal transport that has been observed is mediated by system N. Localization of system N has been controversial. Lee et al. [360] (see also [44]) found that vesicles prepared from abluminal membranes displayed a Na^+^-linked transport for glutamine while vesicles prepared from luminal membranes had only Na^+^-independent transport. This combination would explain net outward flux of glutamine from the brain. However, Ennis et al. [415] found marked Na^+^-dependent tracer influx of glutamine. While there are alternatives (see footnote 3 on p. 9 in [4]) the simplest interpretation is that there are Na^+^-linked transporters in both membranes. More recently immunohistochemical localization studies [416] have shown Snat3 primarily on the abluminal membrane but also on the luminal membrane of brain capillaries. It should be noted that while linking transport of glutamine to that of a single Na^+^ confers a bias towards transport into the endothelial cells, it does not preclude flux in the opposite direction via the same transporter and thus it is possible that Snat3 is responsible for the transport across both membranes.

As already mentioned, Lee et al. [360] found that brain endothelial cells have glutaminase activity, and thus following glutamine transport from ISF into the cells, at least some of the glutamine will be broken down to glutamate and NH_4_^+^. Helms et al. [391, 392] have suggested that some of the glutamate can be metabolized further releasing more NH_4_^+^. As a consequence of metabolism within the endothelial cells, glutamine removal from the parenchyma and glutamine appearance in plasma need not be the same. Glutamine net flux cannot be assessed in isolation.

### Na^+^ and Cl^−^

It has been known for almost 50 years [152, 417] that influx and efflux of Na^+^ and Cl^−^ across the blood–brain barrier are much larger than the net flux [4]. It was proposed by Crone [151, 418] that these apparently passive fluxes might well be paracellular, a suggestion that is still in agreement with all available data [4]. (The partial inhibitions seen in some studies with amiloride derivatives are discussed in Sections 4.3.3 and 4.3.4 of [4]).

The permeability of the blood–brain barrier to Na^+^ was measured by Davson and Welch in 1971 [417] and subsequently using a different experimental and analytical approach by Smith and Rapoport in 1986 [419] (see Appendix E). Because the fluxes in and out across the barrier are nearly in balance and the potential difference across the barrier is small, the *PS* product measured for influx, ~ 1 µL min^−1^ g^−1^ for each ion, can be used as an estimate for that for efflux, i.e. for the clearance via the barrier (see Appendix A). This cannot be exactly true, because there is a component of active transport, but inhibition of the Na^+^-pump has little effect on the tracer fluxes. This is consistent with both passive influx and passive efflux being much larger than both active transport and the net flux, and with active transport making a major contribution to the net flux (see Section 4.3.5 in [4]).

Perivascular transport does make a contribution to the clearances of Na^+^ and Cl^−^. Perhaps more importantly the *net* perivascular transport of each, the difference between influx and efflux, will be closely similar in size to the net transport across the blood–brain barrier, so that the volume, Na^+^ content and Cl^−^ content of the parenchyma can be constant. The net transport of each across the blood–brain barrier is close to its concentration times the rate of fluid secretion across the blood–brain barrier in the steady-state. The controversy over whether perivascular influx and efflux occur along the same vessels or instead there is a glymphatic circulation with influx primarily by periarterial routes and efflux primarily by perivenular routes was considered in Sect. 4.2.

The net transports across the blood–brain barrier and via perivascular routes need not be exactly equal because there will be some component of diffusion between ISF and CSF at the brain surfaces, e.g. across the ependyma lining the ventricles and across the pia/glial layers. As indicated in Fig. 2 (see also [420], blood vessels enter and leave the parenchyma from subarachnoid spaces and cisterns and not from the ventricles. Thus transport from parenchyma to the ventricles will be primarily by diffusion probably with a component of flow in white matter (see Footnote 2) but it cannot be perivascular.

The possibility that there can be a small but significant net perivascular outflow from the parenchyma of Na^+^, Cl^−^ and accompanying water may be the resolution of a long-standing difficulty. In non-communicating hydrocephalus, CSF production by the choroid plexuses continues at a nearly normal rate, but the normal route for CSF outflow from the III^rd^ ventricle is blocked. Because after an initial period the ventricles do not continue to enlarge at a rate sufficient to accommodate the CSF production, CSF must be escaping via an alternative route (see Sections 4.2.2.1–4.2.2.2 in [41] and Section 4.1 in [4] for discussion and references). The periventricular parenchyma is oedematous which may allow flow of fluid from the ventricles, but the oedema only extends a small distance. In cats with kaolin induced hydrocephalus, Sahar et al. [421] observed penetration of serum albumin only up to about 2.5 mm which they took to mean that the albumin was being absorbed into the blood. There is no known mechanism by which this absorption could have occurred. It would be very interesting to know whether this distance corresponds instead to the distance from the ventricular surface to perivascular pathways that would allow sufficiently rapid removal of albumin to CSF in the subarachnoid spaces and/or to lymph that the concentrations observed deeper in the parenchyma would be small. The importance of fluid escape from the ventricles across the ependyma into the parenchyma in hydrocephalus has recently been given further support by observations of gadobutrol movements in normal pressure hydrocephalus in humans [15].

### Amyloid-β

Accumulation of amyloid-β (Aβ) in plaques within the parenchyma and deposition in the walls of arteries are both closely associated with the development of Alzheimer’s disease. Because the rate of production of Aβ appears not to be altered in the more common, late onset form of Alzheimer’s [422] attention has focused on the possible defects in clearance of Aβ that may lead to its accumulation. Aβ may be removed from the brain via metabolism within the parenchyma, via efflux across the blood–brain barrier or or via perivascular efflux [52]. Attempts to estimate the relative importance of each of these routes were reviewed by Hladky and Barrand [146]. For low nanomolar ISF concentrations, which are in or above the normal or clinical range (see [423]), Aβ is eliminated by all three routes, but efflux via the blood–brain barrier is likely to be the most important (see also [424]). However, as emphasized in a key early study, efflux across the blood–brain barrier is saturable with a half-maximal concentration of only 15 nM [62]. Many studies of Aβ metabolism have used much higher concentrations, e.g. > 1 µM, and at these concentrations metabolism is dominant. A recent study on appearance of Aβ in lymph nodes may also reflect the behaviour at higher concentrations as it was performed in mice with mutant APP and high Aβ production rate [425].

Differences in Aβ clearance between sleep and wakefulness have been reviewed by Hladky and Barrand [146] and by Boespflug et al. [426] who emphasized the role of ISF-CSF exchange. The effects of sleep were found to be more complicated than a simple increase in perivascular clearance. Both reviews [146, 426] should be consulted for more detail and discussion (see also Sect. 3.3).

Aβ polypeptides are produced by neurons (and to some extent by other cell types) by cleavage of the membrane bound amyloid precursor protein (APP) [427]. While there is still uncertainty, the final cleavage step is thought to release Aβ directly into ISF.

Most work has focussed on Aβ_1-40_ and Aβ_1-42_, these being the predominant forms of the Aβ polypeptides present in the parenchyma. In solution at or below low nanomolar concentrations they exist as monomers and, particularly for Aβ_1-42_, also as oligomers [423, 428, 429]. Only soluble forms of Aβ are detectable in young animals. However, in older animals and older people deposits mainly of Aβ_1-40_ accumulate along cerebral arteries (cerebral amyloid angiopathy or CAA) and large aggregates or plaques mainly of Aβ_1-42_ form in the brain parenchyma. Small changes in soluble Aβ concentrations may over time lead to large changes in the formation of Aβ aggregates [430–436]. While it is not known which forms of Aβ are toxic, current evidence appears to suggest that within the parenchyma the main culprits are the oligomers [437–441].

There is evidence that plaques in the brain can be removed by reducing the ISF concentration of Aβ [428, 442]. However, it is likely that this only occurs if the Aβ concentration can be reduced to levels below those present before aggregate formation began [431]. This has been shown experimentally but it may not be achievable in practice without both inhibition of Aβ production (see e.g. [443]) and enhancement of Aβ clearance.

#### Clearance of Aβ from ISF

In the young, Aβ is present in soluble form and is eliminated as rapidly as it is produced with about 7–8% of the total soluble Aβ being replaced each hour [422, 444]. Monomeric and small oligomeric forms of soluble Aβ are cleared from ISF by at least four routes: incorporation into plaques, metabolism [445–451], efflux across the blood–brain barrier [62, 429, 452–454] and efflux via perivascular routes [25, 85, 128, 455]. The relative importance of each of these routes remains controversial [52, 146, 456–458].

##### Evidence for transport of soluble Aβ across the blood–brain barrier

The ways in which soluble Aβ can be transported across the blood–brain barrier have been investigated by several different groups. Shibata et al. [62] were the first to propose that Aβ could cross the blood–brain barrier by transcytosis mediated by low density lipoprotein receptor related protein (LRP1). This they said could account for the loss of ^125^I-Aβ_1-40_ from the brain that they observed. In support of their proposal they found that the loss of total ^125^I from the brain was reduced by antibodies against LRP1, by receptor (LRP1) associated protein (RAP), which interferes with binding of all known substrates to LRP1, and by absence of apoE seen in knockout mice. (ApoE affects the interaction of Aβ with LRP1). In addition the elimination process appeared to be saturable with K_m_ of 15 nM. All of these observations are consistent with the idea that the elimination of soluble ^125^I-Aβ_1-40_ is primarily efflux across the blood–brain barrier and is via an LRP1-dependent process. However it should be kept in mind that demonstrating the importance of LRP1 is not the same as demonstrating elimination via the blood–brain barrier because LRP1 is also present on neurons, astrocytes and vascular smooth muscle cells where it can mediate endocytosis of Aβ leading to its metabolism inside the cells [448, 456, 459] (see [146] for further discussion). Further results supporting the involvement of efflux have been reported by Bell et al. [429], who found that the rate constant for elimination of Aβ_1-42_ was about half that for Aβ_1-40_ and also in other papers by Deane, Zhao, Nelson, Zlokovic and coworkers [452, 454, 460].

Results from several other groups also support the idea that efflux of soluble Aβ does occur at the blood–brain barrier and that LRP1 is involved in this elimination.

- Jaeger et al. [461] showed that antisense oligonucleotides against LRP-1 substantially decreased the loss of Aβ_1-42_ after intraparenchymal injection.
- Pflanzner et al. [462] demonstrated LRP1-dependent Aβ_1-40_ transport across monolayers of primary mouse brain capillary endothelial cells, a transport not observed in monolayers of cells with genetically modified LRP1.
- Roberts et al. [457] confirmed that efflux of Aβ from brain to blood occurs in vivo by finding that the concentration in venous blood leaving the brain was 7.5% higher than that in arterial blood.
- Qosa et al. [424] using the brain efflux index method found that 62% of added ^125^I-Aβ_1-40_ appeared in the blood.
- Storck et al. [453] developed a mouse model in which LRP1 could be knocked out selectively in endothelial cells and showed that the knockout reduced the initial rate of loss of ^125^I-Aβ_1-42_ by 48%.

**Fig. 20:**
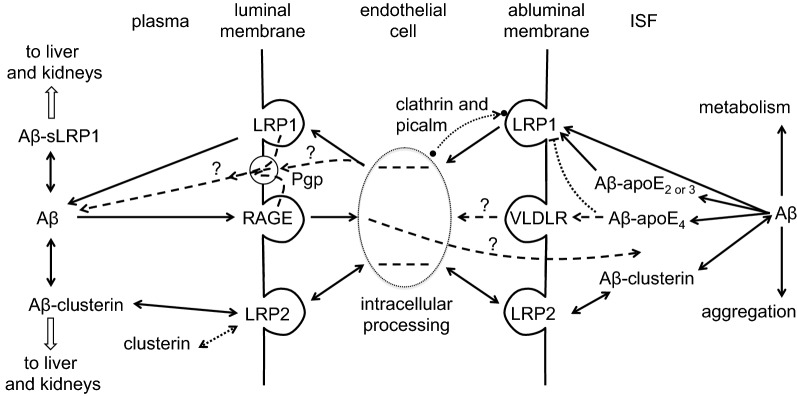
Simplified outline of Aβ transport across the blood–brain barrier. Possible movements of Aβ are shown by solid or dashed lines with arrowheads indicating the principal direction. Endocytotic and exocytotic vesicles are shown as invaginations of the membranes. There is intracellular processing once the vesicles have been endocytosed. Aβ from ISF can bind directly to LRP1 on the abluminal membrane with the complex then being incorporated into a clathrin coated pit which can be endocytosed. The Aβ-LRP1 complex is stabilized by binding of the phosphatidylinositol-binding clathrin assembly protein (PICALM). Aβ in ISF can also be complexed with any of the forms of apoE, 2, 3 or 4 or with clusterin. Aβ-apoE_2_ and Aβ-apoE_3_ are substrates for interaction with LRP1 and endocytosis. By contrast Aβ-apoE_4_ inhibits LRP1 mediated endocytosis (dotted line), but can be endocytosed slowly after binding with the very low density lipoprotein receptor (VLDLR). Aβ-clusterin is a substrate for LRP2 mediated endocytois with transport across the blood–brain barrier to plasma. As Aβ-clusterin can also be transported in the opposite direction by LRP2-mediated endocytosis this is almost certainly by transcytosis of vesicles with LRP2 in the membrane. Vesicles with LRP1 in the membrane are also thought to discharge their contents on the far side of the barrier—i.e. this is transcytosis [465]. Some of the intracellular processing steps for the LRP1 vesicles are now known [452, 658]. Aβ is also transported from plasma to ISF. Aβ clusterin can be transported by LRP2 vesicles, but on the plasma side almost all of the LRP2 receptors are occupied by clusterin (dotted double headed arrow) rather than Aβ-clusterin which greatly reduces blood-to-brain transport by this route. Aβ is however, endocytosed after binding to the receptor for advanced glycation products, RAGE, and somehow transported to the brain side. Pgp may, in a manner which has not been well defined, assist transfer of Aβ from the endothelial cells to plasma whether it has entered the cells from ISF, via the LRP1 system, or from plasma, via the RAGE system. Figure based on [452, 464, 465]

Collectively the studies discussed above leave little doubt that LRP1 dependent transport across the blood–brain barrier plays a substantial role in Aβ elimination. However, the actual mechanisms governing the net inward or outward flux of Aβ across the blood–brain barrier are considerably more complicated and involve complexing Aβ with soluble factors including clusterin (also called apoJ), apoE and a soluble, truncated form of LRP1 (sLRP1). In addition there are at least four endocytotic/transcytotic systems. Figure 20, based mainly on the views of Zlokovic and colleagues [429, 452, 454, 460, 463–466], is a simplified diagram indicating the mechanisms of Aβ transport across the blood–brain barrier. Notable in this scheme is the involvement of apoE, clusterin and the phosphatidylinositol-binding clathrin assembly protein, PICALM (also called CALM). Genetic variations for each of these have been shown to be associated with increased risk of Alzheimer’s disease [467, 468].

Much of the soluble Aβ in ISF may be in the form of complexes with apoE or clusterin while in plasma most Aβ is complexed with clusterin or sLRP1, a truncated, soluble form of LRP1 [469]. The apoE gene has three alleles called apoE_2_, apoE_3_ and apoE_4_. Expression of the apoE_4_ allele is the greatest genetic risk factor known for developing the late-onset form of Alzheimer’s disease [467, 468].

LRP1 mediated transport of Aβ occurs via clathrin pits, with the LRP1, Aβ, clathrin system stabilized by interaction with PICALM. In addition to this transport of Aβ there is LRP1-mediated transport from brain-to-blood of Aβ complexes with apoE_2_ or apoE_3_ and LRP2-mediated transport of Aβ complexes with clusterin. Complexes with apoE4 inhibit LRP1-mediated transport but are transported at a much lower rate by very low-density lipoprotein receptor (VLDLR) mediated transport. This inhibition and slow transport with the resulting tendency to accumulate Aβ in the brain may account for the increased risk of Alzheimer’s disease.

The receptor for advanced glycation end products (RAGE) mediates transport of Aβ from blood-to-brain. Aβ-clusterin blood-to-brain transport by LRP2 can also be demonstrated under experimental conditions, however, in vivo it is likely that the Aβ-clusterin complexes are out-competed by clusterin for inwards transport [470–472]. The net flux of complexes via LRP2 is thus brain-to-blood [429, 464]. sLRP1 is released from LRP1 at the luminal membrane by removal of the membrane binding domain. Aβ complexes with sLRP1 are apparently not transported across the blood–brain barrier but can be delivered to the liver. Thus these serve as a sink reducing backflux of Aβ that has emerged from the brain [469].

The role of *p*-glycoprotein (Pgp) has been considered in many studies [243, 473–490] that indicate that it does play a role, but there have also been studies suggesting that it does not [491–494]. *P*-glycoprotein is present in the luminal membranes of the endothelial cells (see Sect. 4.2.1). With LRP1 mediating entry of Aβ into the endothelial cells from ISF, an obvious role to suggest for *p*-glycoprotein is Aβ efflux to plasma. Another function of *p*-glycoprotein may be to return to plasma some of the Aβ brought into the cells by RAGE [423, 495, 496]. However the intervening steps between endocytosis mediated by either LRP1 or RAGE and efflux by *p*-glycoprotein remain to be established.

The overall net flux of Aβ across the blood–brain barrier is thus seen to be the resultant of a number of transport mechanisms mediating both inward and outward fluxes. The use of complexing agents in plasma to reduce Aβ flux from blood-to-brain is one strategy being tried to reduce Aβ accumulation in the brain.

##### Evidence for Aβ elimination via perivascular routes

The perivascular route has also been considered as a likely pathway for elimination of Aβ peptides from the brain. In initial studies, following exogenous Aβ introduction into the brain, aggregates were first found along the external boundaries of arterial walls [497, 498] (see also [499, 500]) but at later times were seen throughout the smooth muscle layer of the arteries ([497], see also [501]). The results from these initial studies are consistent with the idea that growth of the deposits starts occurring adjacent to an efflux route for Aβ along the outside of the arteries, i.e. an extramural periarterial route.

Subsequent studies followed the routes of exit from the parenchyma of fluorescent dextran. This was used as a non-metabolizable marker for substances of the size of Aβ. Within minutes of its injection fluorescence could be visualized throughout the smooth muscle layer of the arterial walls [70]. From this observation it was proposed that both the fluorescent dextran and the Aβ enter the smooth muscle layer near its end closest to the capillaries and move along the vessel wall towards the subarachnoid space with little further exchange between the smooth muscle layer and the surrounding parenchyma. However, it remains difficult to see how there could be sufficient driving force for movement through the extracellular matrix along the entire length, perhaps a millimeter, of the vessel (compare the discussion in Sect. 3.2.1) while at the same time movement over a 10- to 20-fold shorter distance perpendicular to the vessel wall is prevented. For a different viewpoint see [88, 95, 502–504]).

**Fig. 21:**
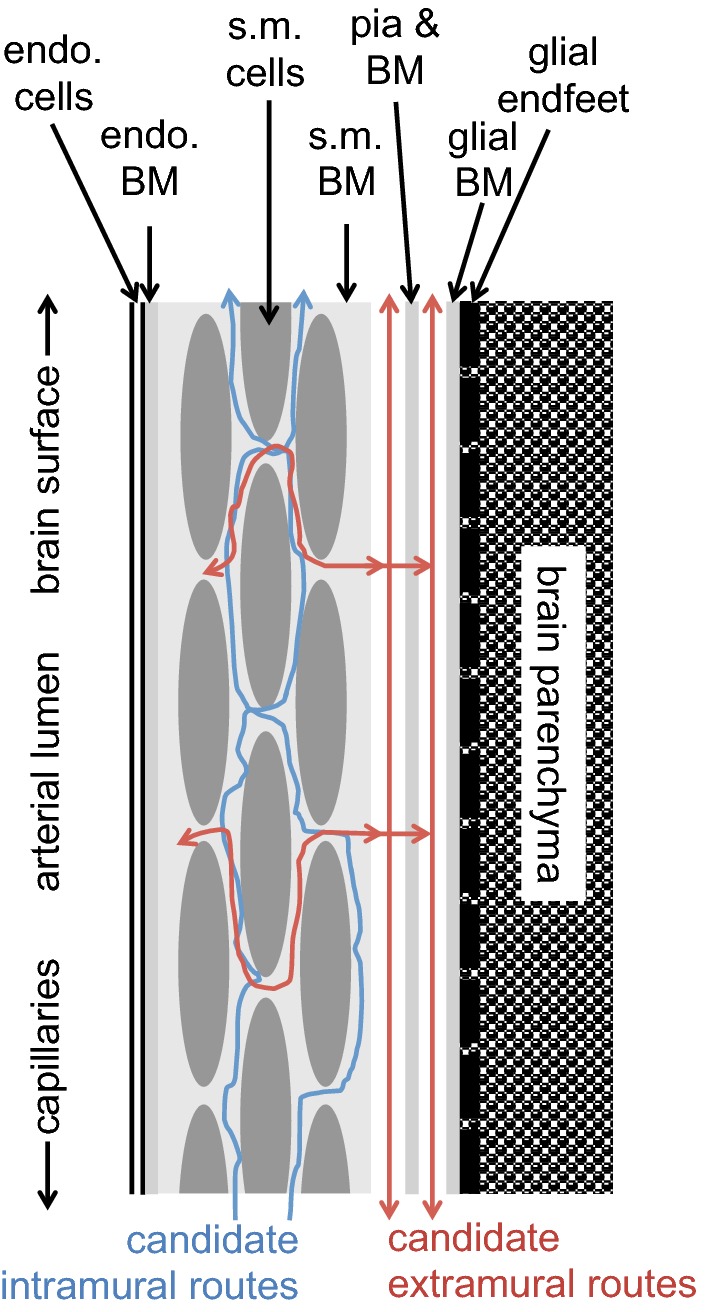
Putative routes for periarterial efflux. In the intramural proposal solutes move parallel to the vessel wall along the basement membranes of the smooth muscle layer, shown as blue trajectories. In the extramural proposal movements of solutes parallel to the vessel occur primarily in a perivascular space with lower resistance to flow. They also move in and out of the wall by a combination of diffusion and convection, shown as the red trajectories. As discussed in Sect. 3.1 the nature of the extramural pathway is still controversial including whether it is a space one side or the other of the pial cells or alternatively the pial and glial basement membranes themselves. *endo* endothelium, *s.m* smooth muscle, *BM* basement membrane. Pial cells and pial basement membrane(s) are shown together because they are very thin

There may be an alternative explanation. The higher observed density of dextran or Aβ within the extracellular spaces of the smooth muscle layer than in the interstitial spaces of the parenchyma [102] might suggest that it binds, reversibly, to some component of the extracellular matrix in the layer. There is in fact good evidence for interaction of the Aβ peptides with some components [505, 506]. If the high concentrations within the basement membranes of the layer reflect binding rather than some form of impermeant sheath, then it is not clear whether Aβ and the dextrans reach the sites of the binding by moving parallel to the vessel wall or by traversing it (see Fig. 21). If the latter, movements parallel to the vessel would be occurring via an extramural route that might have a much lower resistance to flow. Transverse movement has been observed for both horseradish peroxidase and 3H-leucine with large cerebral arteries [507], and no additional impermeant layer is known to exist around smaller arteries inside the parenchyma [98]. There is at present no compelling evidence to decide between the intramural and extramural routes for movement parallel to the vessels.

The importance of the perivascular route for Aβ elimination may be not so much that it removes Aβ from the parenchyma but rather that it delivers Aβ into the vessel walls of arterioles and arteries. Cerebral amyloid angiopathy is often seen before formation of senile plaques within the parenchyma (see e.g. [508]) and the damage to the arterioles and arteries may have secondary consequences for the well-being of parenchymal cells, either by effects on blood flow or via local inflammation [509–511].

#### Relative importance of metabolism, blood–brain barrier transport and the perivascular route for elimination of soluble Aβ

Attempts have been made to estimate the proportions of soluble Aβ removed from the brain by metabolism, by transport across the blood–brain barrier, and by perivascular efflux. It is possible to get an estimate of perivascular elimination alone using inulin. When this was done in mice, Shibata et al. [62] found that the half-time for the elimination of ^125^I-Aβ_1-40_ was much shorter than could be explained by elimination by the perivascular route, with calculated rate constants of 0.027 min^−1^ and 0.0029 min^−1^ respectively (see Table 5). As they had concluded that metabolism played little part, the faster, non-perivascular elimination was held to be transfer across the blood–brain barrier. Bell et al. [429] (see Appendix 2 in [146] for corrections to their calculations) extended these observations to ^125^I-Aβ_1-42_.

It is interesting to note that Xie et al. [128] found the half-lives for both Aβ and inulin to be different when the mice were asleep as compared to when they were awake. In both conditions the rate constant was larger for Aβ than for inulin (see Table 5). The interpretation of these differences in rate constants between wakefulness and sleep has been considered in some detail in [146] and will not be considered further here.

**Table 5:** Rate constants for elimination of ^125^I-Aβ_1-40_, ^125^I-Aβ_1-42_ and inulin in mice

	Rate constant/min^−1^			
	Shibtata et al.	Bell et al.	Xie et al. awake	Xie et al. asleep
Inulin	0.0029	0.0024	0.006	0.016
Aβ_1-40_	0.027	0.0184	0.024	0.053
Aβ_1-42_		0.011		

Data from Shibata et al. [62], Bell et al. [429] (see Appendix 2 of [146]) and Xie et al. [128]

The results of Shibata et al. [62], Iliff et al. [25] and Xie et al. [128] all imply that the rate constant of perivascular elimination, as estimated by the constant for inulin efflux, is considerably less than the rate constant of elimination by other means.^21^

Roberts et al. [457] sought to compare rates of metabolism of Aβ with those of Aβ efflux. To do this they used values for: the turnover rate for Aβ [512]; the pool size for Aβ; the difference between Aβ concentrations in arterial blood and in venous blood leaving the brain; the cerebral blood flow and the rate of return of CSF to the general circulation. From these values they calculated that 25% of Aβ elimination was via efflux across the blood–brain barrier, 25% was via CSF and the remaining 50% was via metabolism. As discussed in [146] while the results of Roberts et al. do suggest that all of these mechanisms are involved, the fraction of Aβ leaving the brain across the blood–brain barrier may have been underestimated and could be as high as 50%. By contrast the fraction accounted for by metabolism may have been smaller than estimated.

On balance the available data suggests a significant involvement in elimination of Aβ from the brain for all three routes of elimination: metabolism, net outward flux across the blood–brain barrier and net perivascular outward flux.

#### Estimating the value of the total clearance of soluble Aβ from ISF

Calculating a clearance value for the elimination of Aβ from ISF is not straightforward as much of the Aβ in ISF is complexed with other solutes, e.g. apoE and clusterin. However, an estimate can be made if it is assumed that all the forms that are accessible to be eliminated are dissolved in the ISF and eliminated with the same rate constant. The volume of distribution for the total soluble Aβ, whether or not as part of complexes, will be that of ISF and thus the clearance can be calculated as rate constant × volume of distribution = 0.05 min^−1^ × 0.2 mL g^−1^ = 10 µL g^−1^ min^−1^. On this basis perivascular clearance, expected using the same assumptions to be about 1 µL g^−1^ min^−1^, may be about 1/10th as large, a small but still significant fraction of the total.

In all of the preceding, the rates of elimination by various routes have been considered almost as if they are constant. However, reduction in the overall clearance and thus in the rates of elimination by some of the routes are likely to be very important in the development of Alzheimer’s disease [422]. In this regard LRP1 expression has been found to be reduced and RAGE expression increased with age [478, 513]. Similarly perivascular elimination has been found to decrease with age possibly as a result of decreased variations in the size of arteries and arterioles during the cardiac cycle [514] (see Sect. 3.2). All of these changes will tend to increase Aβ ISF concentration and hence lead to increased formation of plaques and vascular Aβ deposits.

## Maintenance of brain ISF composition

Some substances in ISF simply need to be expelled, others must be eliminated in a more controlled manner to allow a stable concentration. For most xenobiotics or waste products, the objective is simply to get rid of the substance and keep the extracellular concentration as low as is practical. However, for a number of substances, the objective is to achieve the proper balance between influx, production, consumption and elimination so that their ISF concentrations can be kept within an acceptable range. The objective in this section is to consider how control of ISF concentrations is achieved.

There are several substances whose ISF concentrations must be kept within narrow limits to ensure correct neuronal function. Na^+^, Cl^−^ and K^+^ are good examples. Regulation of Na^+^ and Cl^−^ amounts and concentrations is inextricably linked to the control of extracellular fluid volume and intracranial pressure and is outside the scope of this review (for some discussion see [41]). The control of K^+^ and HCO_3_^−^ ISF concentrations was considered in [4]. The following sections consider the general principles and the control of ISF concentrations of CO_2_ and glucose.

### General principles of concentration maintenance: balancing input and output. CO_2_ as an example

The concentration of a substance can only be maintained at a constant level if its rate of elimination, *R*_*elim*_, is equal to its rate of input, *R*_*in*_,

11 $$R_{elim} = R_{in} .$$

**Fig. 22:**
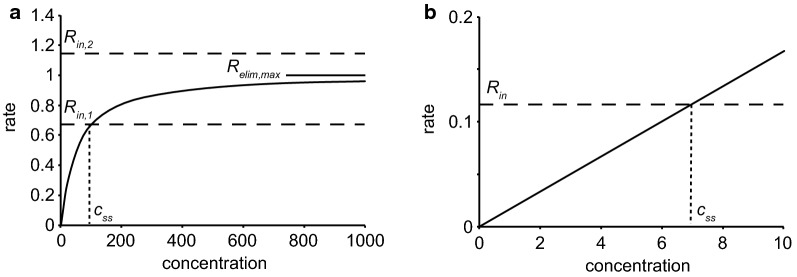
The relation between the rate of elimination of a substance and its concentration. The solid curve in **a** and line in **b** show the rate of elimination as a proportion of its possible maximum versus concentration. Possible rates of input are shown as the dashed lines. In **a** if the rate of input is *Rin*_,*1*_ which is less than the maximum possible rate of elimination, *R*_*elim*,*max*_, the concentration can be maintained at *css*. If the rate of input is *Rin*_,*2*_, which exceeds *Relim*,*max*, no steady-state is possible and the concentration continually increases. At low concentrations as shown in detail in **b** the rate of elimination is usually proportional to concentration

If input exceeds elimination the concentration will increase; if it is less the concentration will decrease. In the face of a given rate of input, be it by influx from outside or local production within the brain, a steady-state can only be achieved if the elimination rate can increase far enough to balance the input (see input *R*_*in2*_ in Fig. 22a). A steady-state is not possible if elimination is unable to match input (see input at level 2) and under these conditions the concentration will continually increase. Thus it is the relative rates of input and elimination, rather than the rate of input itself that is of primary importance.

The rate of elimination of a substance from the brain parenchyma is determined by its concentration and the ability of the efflux mechanisms to remove the substance. This ability is usually described as the clearance. For a substance eliminated by a single type of transport, the clearance is determined by the number of transporters, the affinity-constant for the substrate and the transporter and the maximum turnover rate. Clearance can be calculated from measurable quantities as

12 $${\text{CL}} = {{R_{elim} } \mathord{\left/ {\vphantom {{R_{elim} } c}} \right. \kern-0pt} c}.$$

where *R*_*elim*_ is the rate of elimination and *c* is the concentration of the substance. At sufficiently low concentrations the relation between elimination rate and concentration is linear and the clearance is a constant (see Fig. 22b). At higher concentrations (see Fig. 22a) the relation is no longer linear and the clearance decreases as concentration increases.

**Fig. 23:**
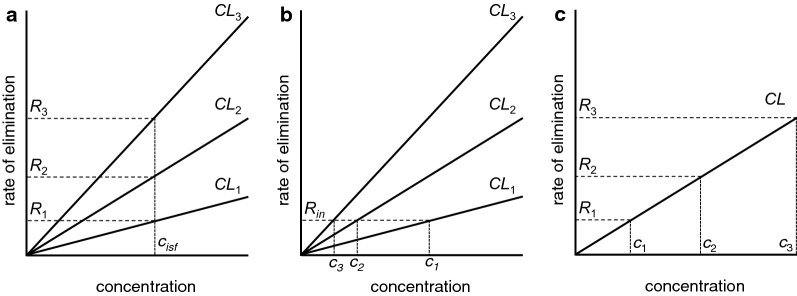
The relationship between the rates of input and elimination, substrate concentration in ISF and clearance. At steady-state the rate of elimination must equal the rate of input. The horizontal dashed lines show rates of input (*R*_1_, *R*_2_, *R*_3_ and *R*_*in*_). The clearance, *CL*, is the slope of the line for the plot of rate of elimination versus concentration. Lines for three values of clearance (*CL*_1_, *CL*_2_ and *CL*_3_) are shown. **a** To achieve the steady-state concentration, *cisf*, clearance must be higher to balance the higher rate of input i.e. the rate of input required is proportional to clearance. **b** For a given rate of input, the steady-state concentration is inversely proportional to *CL* (compare the three steady state concentrations c1 c2 and c3 achievable for the three clearance values *CL*_1_, *CL*_2_ and *CL*_3_). **c** For a given clearance the steady-state concentration is proportional to the rate of input. Changes in input need not produce changes in concentration if the clearance can be changed, e.g. for the increase from *R*1 to *R*3 shown in **a** the concentration would be constant if the clearance could be increased from *CL*_1_ to *CL*_3_

The larger the clearance, the higher the rate of elimination possible at any given concentration (see Fig. 23a) and therefore the lower the concentration needed to achieve an elimination rate equal to a particular rate of input, *R*_*in*_, (see Fig. 23b), i.e.

13 $$c = {{R_{in} } \mathord{\left/ {\vphantom {{R_{in} } {CL}}} \right. \kern-0pt} {CL}}.$$

When the clearance is constant, changes in input (*R*_1_, *R*_2_, *R*_3_ in Fig. 23c) lead to proportional changes in steady-state concentration. Such changes in ISF concentration may be fine if the ISF concentration is not critical. Constant clearance avoids the disasters that could occur if the elimination rate could not increase with ISF concentration because then increased rate of input would produce progressively increasing concentration within the parenchyma.

If close control of ISF concentration is required there must either be some means to reduce or prevent changes in input or the clearance must alter. When input is from plasma one way in which changes in input can be made less sensitive to plasma concentration is for the input mechanism to be operating not too far from its maximum rate, i.e. for the substrate concentration in plasma to be well above the *K*_*m*_ for the input mechanism. However, the same limitation may apply to efflux as to influx, with the resulting changes in ISF concentration difficult to predict (e.g. for glucose, see Fig. 14 and Appendix D).

If input is determined by production within the parenchyma, closer control in the face of variable input than would be seen for constant clearance must be achieved by altering the mechanisms of elimination to change the clearance. In order for the system to be modified some sort of signal is required ‘to inform’ the elimination system that the input and/or the concentration has changed.

**Fig. 24:**
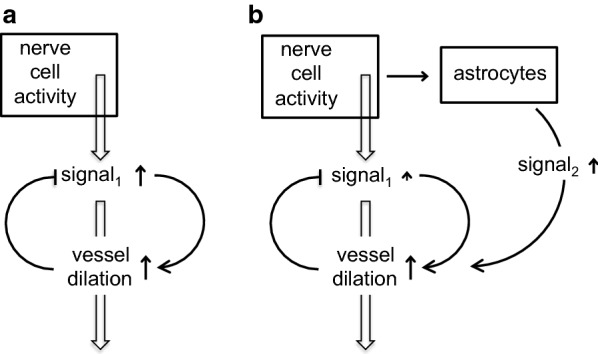
Diagram illustrating possible schemes for neurovascular coupling, i.e. regulation of blood flow changes associated with nerve activity. Two forms of control are shown, **a** simple feedback based on the signal to be regulated, e.g. pCO_2_, and **b** feedback plus feed-forward. The feed-forward element, signal_2_, in **b**, possibly from astrocytes, allows blood flow to increase with smaller changes in the primary quantity to be regulated, signal_1_ (Figure reproduced from [4])

In principle this can be done by feedback control in which increased concentration somehow modifies the mechanism of elimination to increase the clearance, e.g. by recruiting more transporters. To some extent this occurs with CO_2_. Increased pCO_2_ is associated with lower pH and stimulation of cerebral blood flow, which washes away the excess CO_2_ (see Sect. 5.2), i.e. increased pCO_2_ increases the clearance for CO_2_. However, feedback control still requires that there be a change in the concentration to stimulate and maintain the process (see Fig. 24).

Closer control is possible with feed-forward regulation, in which the change in input itself or something closely linked to the input stimulates the change in clearance whether or not the concentration changes. In principle the control could be perfect if somehow a change in input rate could produce proportional change in clearance as indicated in Fig. 23a. It is now clear that increased brain activity, which increases production of CO_2_, increases blood-flow even without increases in CO_2_ concentration. This process, called neurovascular coupling [515, 516], is considered in more detail in [4] which can be consulted for further references.

When substrate elimination is limited by transport across the blood–brain barrier rather than by blood-flow, the clearance can be increased by inserting more transporters, by increasing the activity of each transporter, i.e. an increase in the turnover rate or, if the transport isn’t saturated by increasing the affinity of the transporter for the substrate.

### Achieving a net flux: glucose as an example

There is regulation of transport across the blood–brain barrier both for glucose and for ions like Na^+^, K^+^ or Cl^−^. Regulation of glucose transport serves primarily to achieve the correct flux to support metabolism whereas regulation of ion transport is important to maintain the correct concentrations in extracellular fluid. The actual glucose concentration in ISF is relatively unimportant so long as it remains well above the *K*_*m*_ for hexokinase (0.04–0.05 mM, see Sect. 5.3) but low enough to avoid formation of unwanted glycation products. The requirements for the regulation of the glucose transporter, GLUT1, were considered in detail by Barros et al. [314] and Simpson et al. [315]. Thus this section considers only the principles and the extent to which regulation can be obtained by altering glucose efflux.

GLUT1 transport across the blood–brain barrier must be capable of producing a net flux that is equal to the cerebral metabolic rate for glucose, *CMR*_*glc*_, at all times both at rest and during nervous activity. Furthermore the system must be capable of increasing net flux quickly to match demand. If the net inward flux were not increased, then for a glucose content in brain of 1.3 mM × 0.77 mL g^−1^ and an increase in glucose consumption rate of 0.65 µmol g^−1^ min^−1^ (i.e. to twice resting level, figures for rats), the entire glucose reserve would be consumed in < 2 min.

*CMR*_*glc*_ of stimulated nervous tissue isn’t easy to measure, partly because a region large enough to assay is likely to contain both stimulated and unstimulated tissue. Using quantitative autoradiography in rats exposed to monotonic sounds, Cruz et al. [517] were able to see as much as 85% increase in *CMR*_*glc*_ in tonotopic bands of the inferior colliculus. Using PET imaging in human subjects viewing a reversing checkerboard pattern, Fox et al. [518] saw 50% increases in the visual cortex. Using measured arterio-venous concentration differences in human volunteers undergoing exhausting cycling or rowing exercise, Quistorff et al. ([353], data from [519]) found more than twofold increases in glucose uptake rate across the blood–brain barrier. (There was also a substantial uptake of lactate). From these and other studies, in order to support nervous activity it must be possible to increase the net flux across the blood–brain barrier by at least twofold within a few minutes.

There are three important steps in the delivery of glucose: arrival in the blood; net transport across the blood–brain barrier; subsequent diffusion to the sites of hexokinase. At rest, the blood flow delivers 5–10 times more glucose than does the net flux across the blood–brain barrier into the parenchyma. As a consequence the glucose concentrations in arterial blood and the capillaries are similar, and increasing blood flow can only produce modest changes in capillary concentration and the net inward flux into the parenchyma. Both diffusion within the parenchyma and transport across astrocyte and neuron membranes have been found to be fast (see Sect. 5.3). Thus the rate-limiting step in delivery of glucose to regions where it is required in the parenchyma is its transfer across the blood–brain barrier.

Increased glucose consumption by cells within the parenchyma will reduce glucose *c*_*isf*_ and so reduce glucose efflux, resulting in increased net inward flux. Because the Michaelis–Menten constant, *K*_*m*_, for hexokinase is so low, the concentrations of glucose inside the cells and in ISF can be reduced to values much smaller than that found during times of low nervous activity. The size of this effect can be seen in the data of Betz et al. [327] as described in Appendix D. From that analysis there would be an increase of about 40% in the net inward flux, even if there were no change in transport capacity.^22^ Decreased glucose efflux is an important part of the response to increased nervous activity but it is not sufficient on its own to support demand [314, 315, 322]. Decreased efflux has the advantage that it occurs rapidly with the increase in glucose demand.

Changes in GLUT1 transporter expression have been documented and reviewed elsewhere [322, 336, 520]. However, such changes are too slow to provide minute to minute changes in response to nervous activity. As discussed by Cura et al. [322] there are two types of changes that may occur quickly (see following), one is recruitment of additional preformed GLUT1 from intracellular stores and the other is an increase in transport rate for the existing GLUT1. Both may be occurring. There are suggestions that these changes may result in large effects, but there is no clear evidence of which if any are important at the blood–brain barrier.

With regard to GLUT1 recruitment to the cell surface, it can be detected not only on the luminal and abluminal membranes but also on vesicle membranes within the cytoplasm of brain endothelial cells [521]. Early studies on recruitment in a number of tissues were reviewed by Carruthers [328]. Subsequently it has been found that activation of AMP protein kinase (AMPK) by AMP when AMP is produced from ATP in response to nerve activity can in turn lead to recruitment to the cell surface. With cell culture systems including brain endothelial cells recruitment in response to AMP can be large, resulting in a two to threefold increase in GLUT1 at the plasma membrane [322, 522, 523].

With regard to modification of GLUT1 transport rate, it is known from studies on red blood cells that GLUT1 can be substantially inhibited by binding of ATP, an effect that is inhibited by AMP. When ATP hydrolysis is stimulated, ATP concentrations decrease and AMP concentrations increase, both of these events acting to release inhibition of GLUT1 [322, 524]. This effect can be large, a four to tenfold increase in glucose transport. Because increased AMP can increase both recruitment and activity of GLUT1 at the cell surface, it is easily imagined that small changes in AMP levels in endothelial cells could increase glucose transport sufficiently to support increased nervous activity.

## Summary

Substances can be eliminated from the brain parenchyma either by metabolism or efflux. This review considers efflux, which can occur via perivascular routes or via the blood–brain barrier. The quantitative importance of these different mechanisms is assessed using clearance defined as the rate of elimination of the substance from interstitial fluid (ISF) divided by its ISF concentration (see Appendix A). If the rate of elimination and the concentration can both be measured, the clearance is calculated using this definition. Often, however, it is calculated from the half-life and volume of distribution of the substance as explained in Appendix A. The total clearance of a substance is the sum of its clearances by all mechanisms.

Table 6 (see also Tables 1 and 7) lists a number of substances that are cleared by different mechanisms together with indication of the values of their clearances. The last row in the table indicates the clearance of markers for perivascular elimination. These are substances that are known to be neither transported across the blood–brain barrier nor metabolised at a significant rate but leave entirely by perivascular routes (see Appendix B). Each of these markers has a total clearance that is similar to the others. Every water soluble substance in ISF will have a total clearance at least this large, about 1 µL g^−1^ min^−1^, because the clearances by other mechanisms will be added on top of this basal value.

**Table 6:** Overview of efflux routes showing clearance values for substances leaving the brain parenchyma from ISF

Substances	Features	Clearance/µL g^−1^ min^−1^
Passive, non-specific transfer across the blood–brain barrier		
H_2_O^a^, CO_2_^b^, O_2_^c^, NH_3_^c^	Very small molecules	1000–7000^a^, > 6500 large^b^
Methanol, ethanol, antipyrine, isopropanol	Highly lipid soluble molecules^d^	> 100
Glycerol, ethylene glycol, butyric acid	Moderately lipid soluble^d^	100 > CL > 10
Transfer across the blood–brain barrier by specific transporters		
Glucose^e^	Via GLUT1	50–100
Lactate^f^	Via MCT1	60–100
Many substrates^g^	Via Slc22 and Slco transporters	11–364
Amino acids^h^	Via L, A, ASC, N, y^+^, EAAT and others	See^h^
K^+i^	Via several routes including the Na^+^ pump and NKCC1^i^	11.3
Efflux via blood–brain barrier and perivascular fluxes		
Amyloid-β^k^	Primarily across blood–brain barrier	~10^k^
Na^+ j^, Cl^− j^ mannitol^l^	Via both blood–brain barrier and perivascular routes	c. 1–2
Efflux via perivascular routes only		
Sucrose, inulin, albumin, larger dextrans and PEGs^m^	Used as markers for perivascular efflux	c. 1

Values substantially greater than ~ 1 µL g^−1^ min^−1^ imply that clearance is primarily across the blood–brain barrier rather than via perivascular efflux

^a^See Sect. 5.1

^b^See Sect. 5.2

^c^Clearance known to be large but difficult to measure

^d^See Fig. 8

^e^See Sect. 5.3 and Appendix D

^f^See Sect. 5.4

^g^See Table 1

^h^Net fluxes at blood–brain barrier 1–20 nmol g^−1^ min^−1^, perivascular effluxes (except glutamine) ~ 0.1 nmol g^−1^ min^−1^, glutamine ~ 1 nmol g^−1^ min^−1^

^i^See Appendix E, NKCC1 is the Na^+^, K^+^, 2Cl^−^—cotransporter; ^j^see Appendix E

^k^See Sect. 5.7.3

^l^See Appendix B

^m^Negligible blood–brain barrier clearance, see Sect. 3 and Appendix B

**Table 7:** Blood–brain permeability-surface area products (*PS)* from influx data and calculated blood–brain barrier efflux rate constants, *k*_*eff*,*BBB*_ for mannitol, sucrose and inulin

	*PS*/(10^−2^ µL g^−1^ min^−1^)	*k*_*eff*,*BBB*_/min^−1^
Mannitol		
Ohno et al. [591]	180	0.0090
Amtorp [592]	121	0.0061
Preston et al. [593]	72	0.0036
Sisson and Oldendorf [594]	10	0.0005
Daniel et al. [595]	49	0.0025
Preston and Haas [531] as purchased^a, c^	*68*	*0.0034*
Purified^c^	44	0.0022
Average		0.0040
s.e.m		0.0013
Sucrose		
Ohno et al. [591]	40	0.002
Amtorp [592]	20	0.001
Preston et al. [593]	48	0.0024
Davson and Spaziani # [596]	22	0.0011
Reed and Woodbury [597]	5.3	0.00026
Cameron et al. # [598]	33	0.0017
Preston and Haas [531] as purchased^a, c^	*37*	*0.0019*
Purified^c^	15.3	0.00076
Smith [599]	24	0.0012
Preston and Webster 2002 [600]	11.8	0.00059
Miah et al. [532] radiolabelled^a^	*40*	*0.002*
Miah et al. [532] mass spec.	4	0.0002
Average		0.0011
s.e.m		0.0002
Inulin		
Ohno et al. [591]	1.44–2	0.00008
Amtorp [592]	15	0.00075
Preston et al. [593]	9	0.00045
Reed and Woodbury [597]	< ~ 0.3	0.00002
Daniel et al. [595]	6	0.0003
Smith [599]	< 1.5	0.00006
Preston and Webster [600]	3.9	0.0002
Kakee et al. [601]^b^	*30*	*0.0015*
Average		0.00027
s.e.m		0.00010

The volume of distribution is assumed to be 0.2 mL g^−1^. Italic values are excluded from calculations because ^a^they are for comparison with the accompanying values or ^b^it is an outlier, more than 4 st. dev. from the mean of the other values for inulin. The values for mannitol and sucrose marked ^c^are reported for the radiolabelled substances both as purchased and following purification to remove impurities which might be transported more rapidly (see text). The average for sucrose is significantly different from than that for mannitol, p = 0.012, and that for inulin different from that for sucrose, p = 0.01. Data obtained with rats (except # in rabbits)

Perivascular transport is a relatively non-selective flow-based process. The mechanism and detailed route of this transport have attracted a great deal of attention. Controversies still not finally resolved include: (i) the direction of solute movements and flows in periarterial and perivenous routes (Sects. 3.1 and 3.2); (ii) whether perivascular pathways are spaces containing free fluid or basement membranes (Sect. 3.1” section and Footnote 5); (iii) the driving force for the flows (Sect. 3.2); (iv) whether the immediate destination of perivascular efflux is CSF or lymph (Sect. 3.1, Fig. 6 and Footnote 6); and (v) whether there is enough flow either through the parenchyma or via perivascular routes along capillaries to allow there to be a net periarterial inflow and a net perivenous outflow as proposed in the glymphatic hypothesis (Sects. 3.2 and 3.2.1). The evidence for the involvement of flow or convection in perivascular transport of solutes is convincing but flow appears not to be important for transfers of solutes within the interstitial spaces of the parenchyma. Further work is required before it will be possible to reach a definite conclusion whether or not there is a net inward flow along arteries and net outward flow along veins as proposed in the glymphatic hypothesis.

Transport across the blood–brain barrier can occur by a number of different mechanisms (see Fig. 3). The simplest of these, appropriate for small molecules that are lipid soluble, is *diffusion across the lipid membranes and cytoplasm* of brain endothelial cells (Sect. 4.1 and Appendix C). The blood–brain barrier is the main route for the large fluxes of water measured using tracers (Sect. 5.1), but it is almost certainly not the main route for the net inward flux of water into the brain because it is not the main route for net inward flux of Na^+^ or Cl^−^ (see [4, 41]). The net inward flux of water, which occurs primarily at the choroid plexuses, together with the metabolic production of water, must balance the net outward flow of water as CSF and ISF are returned to blood and/or lymph. With the polar solutes Na^+^, Cl^−^, and mannitol the small fluxes that have been observed in tracer studies may be via a small *“leak” through the tight junctions* (Sects. 4.1 and 5.6 and Appendix B).

For many relatively small polar molecules there are *specific transporters* in the membranes (Sects. 4.2 and 5.2 through 5.6). Not surprisingly GLUT1, the transporter for glucose, is highly abundant in the endothelial cell membranes and glucose transport is rapid (Sect. 5.3 and Appendix D). The need for an increased glucose supply during periods of enhanced nervous activity is considered in Sect. 6.2. While lactate is also transported rapidly across the blood–brain barrier at low concentrations (Sect. 5.4), during nervous activity lactate must to some extent either be transferred to inactive regions within the brain or be effluxed to CSF or lymph. Amino acid transport (Sect. 5.5) is more complicated in that there are many different amino acids that are to some extent inter-convertible by transamination. Furthermore there are many different transporters with differing but overlapping substrate preferences (Sect. 5.5.6). The largest fluxes of amino acids across the blood–brain barrier measured using radiotracers are for the large neutral amino acids. However, these occur via a system that mediates obligatory exchanges of amino acids without resulting in an overall net flux (Sect. 5.5.4). The net inward flux of the large neutral amino acids is small compared to the rate at which they are used to allow synthesis of proteins and the neurotransmitters, glutamate and GABA. This implies substantial reuse of amino acids and the corresponding α-keto-acids when proteins, glutamate and GABA are catabolized within the cells (see Sect. 5.5.5).

*Receptor mediated transcytosis* can transport large molecules across the blood–brain barrier. Amyloid-β is an important example (Sect. 5.7) of a substance primarily eliminated by this mechanism. Amyloid-β can also leave the brain parenchyma via perivascular efflux and this may be important as the route by which amyloid-β reaches arterial walls resulting in cerebral amyloid angiopathy.

The majority of substances listed in Table 6 have total clearances greater than those for inulin and sucrose, which implies that they are leaving the brain by routes in addition to the perivascular pathways. For those whose clearances are not much greater than 1 µL g^−1^ min^−1^, perivascular transport will still make a noticeable contribution. Na^+^ and Cl^−^ enter and leave the brain parenchyma by perivascular and blood–brain barrier routes and both will be important in processes like the development and resolution of oedema (not considered in this review). Non-metabolized substances with clearances greater than about 10 µL g^−1^ min^−1^ leave the brain parenchyma primarily via transport across the blood–brain barrier.

## Conclusion

This review has assessed the evidence from a number of different sources regarding the routes and mechanisms of elimination of substances from the brain parenchyma. Early studies comprehensively and admirably reviewed by Bradbury [55] and by Davson and Segal [56] revealed that there were two important routes, across the blood–brain barrier and via cerebrospinal fluid (CSF). For glucose, O_2_ and CO_2_ it was obvious that exchanges across the blood–brain barrier were dominant. At the other extreme for sucrose and inulin the concentrations in the parenchyma followed those in CSF much more closely than those in blood plasma. For Na^+^ and Cl^−^ it was clear that both routes were important. For certain specific substances, e.g. Ca^2+^, Mg^2+^ and some vitamins and hormones the major route of entry is secretion by the choroid plexuses followed by distribution around the brain in CSF. However, the discovery of specific transporters for many substances at the blood–brain barrier and the finding, based largely on the work of Cserr and associates [82, 83, 126, 525], that efflux of solutes from the parenchyma to CSF was slow with half-lives of many hours led to the view that entry of most substances to the parenchyma was via the blood–brain barrier.

There were early suggestions that movements of large molecules between the parenchyma and CSF could be faster than seen in Cserr’s work. The first of these was the work of Wagner [68] followed by Rennels et al. [69, 135] on entry and exit of horseradish peroxidase. Within an hour of being added to CSF, it could be seen outlining blood vessels deep in the parenchyma. Somewhat later Shibata et al. [62] found that the half-life of inulin was shorter than expected and Groothuis et al. [131] found that when rats were anaesthetized with barbiturates the half-life for sucrose was as Cserr had seen with albumin, but when animals were either awake or anaesthetized with ketamine/zylazine the half-life was much shorter (see Table 1).

The recent explosion of interest in perivascular routes for delivery to and removal of substances from the parenchyma stems largely from observations made using two photon fluorescence microscopy and magnetic resonance imaging [25, 526]. These led to the proposal that there is a “glymphatic pathway” through which CSF flows into and within the parenchyma propelling “the waste products of metabolism into the paravenous space” [136]. As discussed in this review there is convincing evidence for perivascular routes of access to the parenchyma from CSF and also exit from the parenchyma to CSF and/or lymph. However, the balance of available evidence does not support a “glymphatic pathway” for flow through the parenchyma.

Perivascular pathways are the principal routes of elimination of sucrose, inulin, and serum albumin. The blood–brain barrier is the principal site of efflux of many solutes including CO_2_, O_2_, glucose, lactate, K^+^, amino acids, many lipid soluble substances, many substrates of the SLC transporters (Sect. 4.2.2) and a few substrates carried across the endothelial cells by transcytosis. Both routes are important for movements of Na^+^, Cl^−^ and water and both will be important in processes like the development and resolution of oedema (not considered in this review). With important exceptions including Na^+^, and Cl^−^, the available evidence can be summarized with a broad generalization: if there is a transport mechanism for a substance at the blood–brain barrier, then the blood–brain barrier is more important than perivascular pathways for the elimination of that substance.

## Abbreviations, names and symbols

### Abbreviations

α-KG: α-ketoglutarate; α-KIC: α-ketoisocaproic acid; Aβ: amyloid-β = β amyloid; AMT: adsorptive mediated transcytosis; ANLS: astrocyte neuron lactate shuttle (hypothesis); apoE: apolipoprotein E; apoJ: clusterin; A − V difference: the difference between the concentrations in arterial blood entering and venous blood leaving the brain; BCAA: branched chain amino acid (large neutral amino acid); BCH: 2-aminobicyclo-(2,2,1)-heptane-2-carboxylic acid; BCKA: branched chain α-ketoacid (deaminated BCAA); BUI: brain uptake index; CSF: cerebrospinal fluid; ISF: interstitial fluid; LFER: linear free energy relations (Appendix C); LRP1, LRP2: low density receptor related proteins 1 and 2; MAO: monoamine oxidase; MeAIB, αMeAIB: α-(methylamino)isobutyric acid = *N*-methyl-α-aminoisobutyric acid; MW: molecular weight (dimensionless, MW of ^12^C is 12); NMR: nuclear magnetic resonance; PICALM: phosphatidylinositol-binding clathrin assembly protein; RAGE: receptor for advanced glycation end products; RMT: receptor mediated transcytosis; sLRP1: water soluble truncated form of LRP1; TfR: transferrin receptor; THO: tritiated water; VLDLR: very low-density lipoprotein receptor.

### Symbols

$\beta_{2}^{H}$: solute hydrogen bond basicity (Appendix C); *c* (*c*_*isf*_, *c*_*plasma*_): concentration (in ISF or plasma); *CL* (*CL*_*BBB*_, *CL*_*perivascular*_): clearance (via blood–brain barrier or perivascular routes); *CMR*_*glc*_: cerebral metabolic rate of glucose; *D*: diffusion constant; *F*: Faraday constant; Δ*G*_*x/y*_: free energy change for the transfer from *y* to *x* (e.g. Eq. 5); *J*: flux; *J*_*inf*_: water influx (measured using THO); *J*_*net*_: water net flux (in response to an osmotic gradient); *K*_*m*_: Michaelis–Menten constant for an enzyme reaction (e.g. by hexokinase) or transport process; *K*_*t*_: apparent dissociation constant when Michaelis–Menten-like expressions (e.g. Eq. 28) are fitted to flux data for carrier transport; *K*_*x/y*_: partition coefficient from *y* to *x* (e.g. Eq. 5); *k*: rate constant; *k*_*eff*_: rate constant for efflux; *k*_*eff*,*BBB*_: rate constant for efflux across the blood–brain barrier; *N*: amount (not to be confused with N, nitrogen); $\pi_{2}^{H}$ : polarizability (Appendix C); *P*: permeability; *PS*: permeability surface area product; *P*_*w*,*osmotic*_: water permeability calculated from net flux down an osmotic gradient; *P*_*w*,*tracer*_: water permeability measured using tracers; *R*: universal gas constant; *R*_*in*_: rate of input; *R*_*elim*_: rate of elimination; *R*_1_, *R*_2_, *R*_3_: different rates of input (Sect. 6); *R*_2_: excess molar refraction (Appendix C); *S*: surface area of microvessels; *T*: absolute temperature; *T* (Appendix E), period of time during which influx occurs; *T*_*inf*_: influx (Appendix D); *T*_*eff*_: efflux (Appendix D); *T*_*max*_: maximum rate of transport in Michaelis–Menten type equations (Appendix D); *T*_*net*_: net flux (Appendix D); *t*: time; *t*_1/2_: half-life; *V*_*br*_: volume of distribution (Appendix E); $\bar{V}_{brain}$: conversion factor between the mass and volume of the brain, assumed to be 1 cm^3^ g^−1^ (Appendix E); *V*_*D*_: volume of distribution; *V*_*x*_: molecular volume (Appendix C); *z*: charge on an ion, e.g. 1 for Na^+^.

### Amino acid systems and transporters (see Fig. 19)^23^

system A: transport system for alanine and other amino acids, transports MeAIB; system ASC neutral amino acid transporter; system L: large neutral amino acid transport (4F2hc/Lat1 in the rat), transports BCH; system N: Na^+^-linked transport system for large N rich amino acids, e.g. glutamine, see SNAT; system x_c_^−^: cystine, glutamate exchange (mediated by 4F2hc/xCT); system X_AG_^−^: possible glutamate transport in luminal membrane; system y^+^: amino acid transport mediated by CAT1 at blood–brain barrier; system y^+^L: possible transport of neutral amino acids; ASCT2: Na^+^-dependent transporter for neutral amino acids (system ASC); ATA2: Na^+^-linked transporter—alanine preferring (system A); ATB^o,+^: neutral and basic amino acid transporter; CAT-1: cationic amino acid transporter 1; EAAT1, EAAT3: excitatory amino acid transporters 1 and 3 (abluminal membrane); Na^+^-LNAA: Na^+^ linked large neutral amino acid transporter (unknown gene); SNAT3: transporter for N rich amino acids (e.g. glutamine) linked to Na^+^/H^+^ exchange; 4F2hc/Lat1: heterodimeric transporter for large neutral amino acids, obligatory exchanger.

### Other transporters and enzymes

BCRP: breast cancer resistance protein; gdh: glutamate dehydrogenase; GLUT1: glucose transporter 1 (not Na^+^-linked); g.s: glutamine synthetase; MCT1: monocarboxylate transporter 1; MRP1, MRP4: multidrug resistance proteins 1 and 4; OAT, Oat: organic anion transporter in HUMAN or other species; OATp, Oatp: organic anion transporting polypeptide in HUMAN or other species; OCT, Oct: organic cation transporter in HUMAN or other species; Pgp: *p*-glycoprotein; SLC (as in SLc22a8): gene name for a solute carrier (gene for Oat3); t.a: transaminase.

### Additional file

**Additional file 1.** Spreadsheet to be used in fitting the carrier equations described in Appendix 4 to the data reported by Betz et al [327]. A number of fits are provided that can be inspected using Microsoft Excel. Generation of additional fits requires use of the Solver Add-in.

## Figures and Tables

**Fig. 25:**
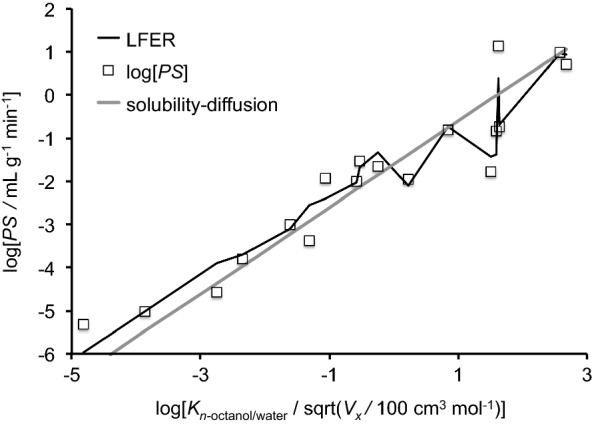
Comparison of the predictions of the solubility-diffusion theory and of the linear free energy relation (LFER) for the *PS* product of the series of 18 compounds considered by Gratton et al. [166]. The solubility-diffusion theory, described in Sect. 4.1, predicts that log[*PS*] is proportional to *K*_*octanol*/water_ MW^−1/2^. MW is in turn approximately proportional to the McGowan characteristic volume used by Gratton et al. The straight, grey line with slope 1 indicates the predicted proportionality. It has one adjustable parameter that determines the vertical position of the line. The LFER prediction, described in this Appendix and shown as the line with multiple segments, has four adjustable parameters, three coefficients of descriptors of the compounds and the constant determining the vertical position of the whole curve. The improvement in fit is statistically significant (extra sum of squares test [640], F = 8.85 for 17 and 14 degrees of freedom, p < 0.001)

## Appendix A. The parameters used in describing elimination from the brain parenchyma

The rate of elimination, *R*_*elim*_, of a substance from the brain parenchyma is determined by its concentration and the ability of the efflux mechanisms to remove the substance. Clearance can be calculated from measurable quantities as

14 $$CL = {{R_{elim} } \mathord{\left/ {\vphantom {{R_{elim} } c}} \right. \kern-0pt} c}.$$

If *R*_*elim*_ is expressed as amount per unit time and the concentration as amount per unit volume, the clearance has the units of a volume flow, e.g. for rate of elimination in mol min^−1^ and concentration in mol mL^−1^ the units of clearance are mL min^−1^. If instead the rate of elimination is expressed per unit mass of tissue, e.g. mol min^−1^ g^−1^, then the units of clearance would be mL min^−1^ g^−1^. For many substances provided the concentration isn’t too large, the rate of elimination is proportional to the concentration and the ratio used to define clearance is actually a constant. For higher concentrations, the rate of elimination may approach a limiting value and the clearance then decreases as the concentration increases (see Fig. 22).

In practice when calculating the clearance it may not be immediately obvious which concentration is appropriate. Ideally it should be the free concentration of the substance. Concentrations that have been used include the exchangeable concentration measured using microdialysis [428] or, for ions, the activities obtained using microelectrodes. The free concentration is not the same as the total concentration; because, for instance, some of the substance may be bound to other things and the substance may be inside cells. The total concentration is the amount of the substance divided by the volume.

Because the amount of a substance is usually much easier to measure than its free concentration, what is reported in studies of elimination is frequently not clearance but rather the rate constant for elimination, k, defined as

15 $$k = {{R_{elim} } \mathord{\left/ {\vphantom {{R_{elim} } N}} \right. \kern-0pt} N}$$

where *N* is the amount present in the region and *R*_*elim*_ is now the rate of elimination from that region. If the units of rate of elimination are mol min^−1^ g^−1^ and those of amount are just mol g^−1^, the units of the rate constant are min^−1^. Strictly for Eq. 15 to apply, *N* must be the amount that can become free as the free concentration is reduced—i.e. it should not include forms that are irreversibly bound, aggregated, or sequestered inside cells.

Because the rate of elimination is equal to both *CL* × *c* (Eq. 14) and *k* × *N* (Eq. 15), the elimination rate constant, *k*, and the clearance, *CL*, are related by *CL* × *c *=* k* × *N*, i.e. *CL*/*k* = *N*/*c*. The ratio, *N*/*c*, has the units of a volume and is usually called the volume of distribution, *V*_*D*_, defined as

16 $$V_{D} = {N \mathord{\left/ {\vphantom {N c}} \right. \kern-0pt} c}$$

and thus

17 $$k = CL/V_{D} .$$

If the units chosen for *N*, the total amount present are mol and the units of *c* are mol mL^−1^ then the units of *V*_*D*_ will be mL. If instead, *N* is the amount present per unit mass of tissue, stated in mol g^−1^, then the units of *V*_*D*_ are mL g^−1^. Because the rate constant, *k*, depends on the volume of distribution, which is determined by the distribution of the substance within the parenchyma, e.g. on binding, sequestration, etc., *k* is also affected by the distribution of the substance and not just on the processes that govern elimination. As a consequence the rate constant is less suitable than the clearance as a description of elimination.

The use of tissue slices to evaluate *V*_*D*_ is discussed in [527, 528].

The half-life for elimination, *t*_1/2_, can be defined as the time taken for the amount present to decrease by half if production of the substance abruptly ceases. When the rate of elimination is proportional to concentration (and the volume of distribution is constant), the concentration decreases exponentially with time, the half-life is constant, and

18 $$t_{1/2} = {{0.69} \mathord{\left/ {\vphantom {{0.69} k}} \right. \kern-0pt} k}.$$

The flux, *J*, of a substance across a membrane or barrier is properly defined as amount transferred per unit area per unit time. However the symbol, *J* is also often used to mean the total amount entering or emerging from a region per unit time. It is usually left to the reader to figure out which: the units are usually the surest guide: for amounts transferred per unit area typical units might be mol cm^−2^ s^−1^ while for amounts entering or leaving a region, mol s^−1^.

The concentration of a substance in ISF is most simply expressed as the amount present per unit volume of ISF,

19 $$c_{isf} = {{\left( {{\text{amount}}\;{\text{in ISF}}} \right)} \mathord{\left/ {\vphantom {{\left( {{\text{amount}}\;{\text{in ISF}}} \right)} {\left( {{\text{volume}}\;{\text{of}}\;{\text{ISF}}} \right)}}} \right. \kern-0pt} {\left( {{\text{volume}}\;{\text{of}}\;{\text{ISF}}} \right)}}.$$

The concentration determined using dialysis or ion selective electrodes comes close to this simplest definition. However, with most chemical or radiotracer assays, what is determined is the amount in a tissue expressed per unit volume of tissue,

20 $$C_{tissue} = {{\left( {{\text{amount}}\;{\text{in}}\;{\text{tissue}}} \right)} \mathord{\left/ {\vphantom {{\left( {{\text{amount}}\;{\text{in}}\;{\text{tissue}}} \right)} {\left( {{\text{volume}}\;{\text{of}}\;{\text{tissue}}} \right)}}} \right. \kern-0pt} {\left( {{\text{volume}}\;{\text{of}}\;{\text{tissue}}} \right)}}.$$

For those substances that are confined entirely to the ISF,

21 $$\begin{aligned} C_{tissue} & = \frac{{\left( {{\text{amount}}\;{\text{in}}\;{\text{ISF}}} \right)}}{{\left( {{\text{volume}}\;{\text{of}}\;{\text{tissue}}} \right)}} = \frac{{\left( {{\text{amount}}\;{\text{in}}\;{\text{ISF}}} \right)}}{{\left( {{\text{volume}}\;{\text{of}}\;{\text{ISF}}} \right)}} \times \frac{{\left( {{\text{volume}}\;{\text{of}}\;{\text{ISF}}} \right)}}{{\left( {{\text{volume}}\;{\text{of}}\;{\text{tissue}}} \right)}}. \\ & = c_{isf} \times \alpha \\ \end{aligned}$$

Current estimates of α are near 0.2 ([24, 74]). If the substance is not restricted to ISF, the more general relation is

22 $$c_{tissue} = c_{isf} \times \frac{{V_{D} }}{{\left( {{\text{volume}}\;{\text{of}}\;{\text{tissue}}} \right)}}.$$

### Appendix A.1. Clearance per unit mass of tissue and permeability surface area product

Clearance and many of the other constants are often stated per unit mass of tissue. Whether *CL* is being used to mean clearance from a named region, e.g. the brain, or clearance per unit mass is usually easy to determine from context. The units, e.g. cm^3^ s^−1^ or cm^3^ s^−1^ g^−1^ respectively, make it clear.

Expressing clearance per unit mass can be particularly convenient when the mechanism is efflux across the blood–brain barrier. For instance if a substance is cleared by passive transport across the barrier at a rate that is proportional to concentration, i.e. the efflux is *J*_*efflux*_ = *Pc*, where *P* is the permeability. The amount transferred per unit time out of unit mass of tissue is *J*_*efflux*_ × *S* =* P* × *S* × *c* where *S* is the surface area of the blood–brain barrier per unit mass of tissue. Because the amount transferred brain-to-blood per unit mass is both *P* × *S* × *c* and *CL*_*BBB *_× *c*, where *CL*_*BBB*_ is the clearance by efflux across the blood–brain barrier, *CL*_*BBB*_ and the *PS* product are synonyms.

The *PS* product is usually measured for influx. If the transport mechanism is passive, then this is also the *PS* product for efflux. (For ions see the next section). Many instances of transport are not well-described using a single value of permeability. For instance if the transport process saturates, the permeability, calculated as observed flux from source to destination divided by source concentration, will decrease as the concentration increases. Results are sometimes reported in terms of permeabilities even when there is known to be an active component of the transport. This allows comparison of the fluxes via active and passive mechanisms, but otherwise permeability is not a good description of active transport. If permeability is to be used, it is necessary to allow the values of *P* to be different for influx and efflux.

### Appendix A.2. Membrane potential and permeability for ions

If a passive transport mechanism transfers a charge *zF* across a membrane and the potential difference across the membrane Δ*V*_0_ (inside minus outside) is the value for zero current via this mechanism then

23 $$\frac{{P_{\text{efflux}} }}{{P_{\text{influx}} }} = e^{{\frac{{zF\Delta V_{0} }}{RT}}}$$

because this is then an equilibrium. If in addition the fluxes are proportional to the concentrations, i.e. the *P*s are independent of concentration, this relation must hold for all potentials and the flux can be written as

24 $$\begin{aligned} J_{net} & = J_{\text{influx}} - J_{\text{efflux}}\\ & = P_{\text{influx}} c_{\text{outside}} - P_{\text{efflux}} c_{\text{inside}} \\ &= P_{\text{influx}} \left( {c_{\text{outside}} - e^{{\frac{{zF\Delta V_{0} }}{RT}}} \;c_{\text{inside}} } \right) \\ \end{aligned}$$

where, however, *P*_*influx*_ can be a function of potential (see e.g. [529, 530]).

For the blood–brain barrier the potential difference is normally small enough (< 4 mV) that when interpreting experimental data for passive transport the potential dependence of *P*_*influx*_ and *P*_*efflux*_ is usually ignored. (It should be noted that potential must be considered explicitly when considering transport into and out of the cells as the potential difference between the inside and outside is much larger than the potential difference between ISF and plasma). The potential difference across the blood–brain barrier changes with pH of plasma and can take on appreciable values (for a review and references see [4]). The consequences of these changes for transport of ions other than H^+^ appear not to have been considered.

## Appendix B. Blood–brain barrier permeabilities of mannitol, sucrose and inulin and the identification of markers for perivascular elimination

Evidences that various substances are markers for perivascular elimination and that elimination to CSF or lymph from the parenchyma is primarily perivascular are intertwined. One of the principal arguments that many substances leave the parenchyma by a convective process is that the clearances for these are all similar despite their being a large range of sizes, e.g. from mannitol and sucrose on one hand, to serum ablumin and many of the polyethyleneglycols (PEGs) and dextrans on the other. These arguments were first advanced by Cserr and associates [126, 127, 129]. However, the data on which they based their argument was obtained under barbiturate anaesthesia, which is now known to greatly suppress the perivascular efflux process.

It is thus important that Groothuis et al. [131] have reexamined the rates of elimination for a range of polar substances including sucrose (MW 342), inulin (MW 5500) and dextran-70 K (MW 70,000) (see Table 1) and have found again that there is no variation in the rate constant for elimination. Others have also measured efflux rate constants, for mannitol and inulin and have found similar values (see Table 1). For many of these substances there is no evidence for transport across the blood–brain barrier. However, for mannitol, sucrose and inulin there appear to be measurable rates of influx, so it must be asked whether they should be included in the list of markers.

The measured rate constant for elimination *k*_*eff *− *total*_, of the putative markers from the parenchyma is the sum of the efflux rate constants for the perivascular and blood–brain barrier routes. Groothuis et al. [131] found values close to 0.24 h^−1^ = 0.003 min^−1^. The rate constant for the passive efflux of a neutral solute across the blood–brain barrier can be calculated from the permeability-surface area product, *PS*, and the volume of distribution, *V*_*D*_, as *k*_*eff*,*BBB*_= *PS/V*_*D*_ (see [127, 131] and Appendix A). Because these substances are restricted to ISF within the parenchyma, *V*_*D*_ can be taken to be 0.2 mL g^−1^. *PS* can be measured from the initial rate of accumulation when the substance is added to the blood. Values are tabulated in Table 7. There is obviously considerable variation in the values found in different studies, which probably reflects the difficulties inherent in measuring small permeabilities. However, several features are apparent. Firstly in each of the three studies that compared mannitol, sucrose and inulin, mannitol had the highest permeability, inulin the least. Secondly the same order is apparent in the averages, 0.0040 ± 0.0013 min^−1^, 0.0011 ± 0.0002 min^−1^ and 0.00027 ± 0.0001 min^−1^. Thirdly for sucrose and inulin the rate constants for elimination by transport across the blood–brain barrier are substantially less than the total rate constant for elimination, 0.003 min^−1^, implying that some other mechanism accounts for most of the elimination. It is at present unclear whether or not mannitol is suitable as a marker for perivascular elimination.

Preston et al. [531] pointed out a major difficulty that occurs in the measurement of very small permeabilities using radiotracers. If the sample of labelled substance contains small quantities of labelled impurities that are more permeable than the principal substance, the impurities will make a disproportionately large contribution to permeability measured by accumulation of the radiolabel. They showed that further purification by thin layer chromatography decreased the measured permeability for mannitol and sucrose (see Table 7). Miah et al. [532] have taken this one step further and have compared the uptake of radiolabel from a sample of ^14^C-sucrose with the uptake of ^13^C-sucrose measured by mass spectrometry. Their measurements using a sample of radiolabelled sucrose yielded one of the higher measured permeabilities, while that obtained using assay of sucrose yielded the lowest. It is thus plausible that perivascular elimination accounts for an even larger fraction of the elimination of sucrose than indicated by comparison of the average of the rate constants in the table with the total rate constant of elimination.

The available evidence strongly supports the commonly held view that sucrose, inulin, albumin, and a number of dextrans are suitable markers for elimination from the brain parenchyma by perivascular convection.

## Appendix C. Passive permeability of the blood–brain barrier: further consideration including the use of linear free energy relations

There are at least four reasons why the permeability-surface area product, *PS*, may not be predicted by comparison with the lipid solubility as assessed using n-octanol and molecular weight, i.e. with *K*_*n*-*octanol*/water_ MW^−1/2^: (1) a biological membrane is more ordered than a layer of n-octanol and thus partition into the membrane core may well differ from partition into the hydrophobic solvent particularly for larger solutes; (2) for large solutes the diffusion constant, even in a homogenous medium, is expected to vary with MW^−1/3^ rather than MW^−1.2^ [156, 157, 533]; (3) n-octanol, with the water it contains in a partition experiment [161], may differ from the membrane core in how it interacts with hydrogen bonding groups;^24^ (4) permeation may occur by pathways other than via the core of the membrane, e.g. transport for some of the substances considered may be via specific transporters (see Sect. 4.2), by transcytosis (see Sect. 4.3) or, particularly for small polar solutes, by a paracellular pathway (as mentioned in Sect. 4.1 an example may be mannitol); and (5) substances which are sufficiently lipid soluble to enter the endothelial cells may be effluxed or metabolised before they even reach the parenchyma (see Sect. 4.2.1).

The difference between partition into a membrane and partition into a liquid like n-octanol may be particularly marked for large solutes [162, 534]. The hydrophobic portions of membranes are composed of hydrophobic side chains of proteins and the chains of lipids both of which have positions constrained by the rest of the protein or the lipid headgroup. Attempts to insert large molecules into a membrane will inevitably require changes in membrane structure, e.g. lipid headgroups may be pushed apart, which will have an energy cost which must affect the permeability. However, it should be noted that the idea that larger molecules are excluded from the membranes was based on their failure to permeate which in many instances may have been because they were substrates for efflux transporters (see Sect. 4.2.1). It is somewhat puzzling that there have not been any attempts to correlate blood–brain barrier permeability with the partition of substances into easily obtained membranes, e.g. those of liposomes or red blood cells, rather than into simple solvents. Partition into red blood cell membranes was measured extensively in studies on the mechanism of general anaesthesia [535].

The predictions of the simple theory presented in Sect. 4.1 for passive, non-specific transport of neutral solutes across the blood–brain barrier describes important features of this transport, but leaves quite large discrepancies between the theoretical predictions and the experimental results (see Fig. 25). A more elaborate approach, based on the use of linear free energy relations has been described by Abraham and colleagues [165, 171, 536, 537]. In this approach each compound is represented by a set of quantitative descriptors that are properties of the substance considered in isolation. (Thus for instance *K*_*n*-*octanol*/water_ is not permitted). These have been chosen as far as possible to represent sufficient independent properties of the substances to allow characterization of their interactions with water, solvents, and sites of action (see below). To predict a property, e.g. the *PS* product, each descriptor is multiplied by a coefficient which depends on the property, but not on the substance. The sum of the products of coefficients and descriptors is the prediction of the logarithm of the property for that substance. Whenever the property concerned is an equilibrium constant, the logarithm of that property is, up to a constant, a free energy. Hence the name “linear free energy relation” as the prediction can be regarded as a linear sum of contributions to a free energy.

Abraham et al. [165] applied this approach to the prediction of partition coefficients from water into a number of solvents, including n-octanol for which

25 $$\log \left[ {K_{{n{\text{-octanol}}/{\text{water}}}} } \right] = 0.088 + 0.562R_{2} - 1.054\pi_{2}^{H} - 3.46\sum {\beta_{2}^{H} } + 3.81V_{x}$$

where the descriptors are: excess molar refraction, *R*_2_; polarizability, *π*_2_^*H*^; solute hydrogen bond basicity, *β*_2_^*H*^, which is summed over the appropriate groups in the molecule; and molecular volume, *V*_*x*_. See [165] and references therein for the rationale for choosing these descriptors and their definitions. The volume term makes the largest contribution to the differences between the 18 substances considered [166] with the larger substances being more soluble in n-octanol.

Gratton et al. [166] have applied this approach to the prediction of *PS* products using 18 substances to determine the values of the coefficients and test the accuracy of the predictions with the result:

26 $$\log [PS] = - 1.21 + 0.77R_{2} - 1.87\pi_{2}^{H} - 2.8\sum {\beta_{2}^{H} } + 3.31V_{x} .$$

This more elaborate theory does allow a statistically significant improvement in the fit of the calculated values of *PS* to those observed (see Fig. 25). Gratton et al. note that there is a strong dependence on molecular volume, with increases in volume being associated with *increases* in permeability.

The LFER approach does improve the ability to predict *PS* for a new substance. But it’s formulation has served to hide a major clue as to the mechanism of permeation. That clue can be revealed by calculating the prediction of the LFER approach for the ratio, *PS*/*K*_*n*-*octanol*/water_:

27 $$\begin{aligned} \log \left[ {{{PS} \mathord{\left/ {\vphantom {{PS} {K_{{n{\text{-octanol}}/{\text{water}}}} }}} \right. \kern-0pt} {K_{{n{\text{-octanol}}/{\text{water}}}} }}} \right] & = \log \left[ {PS} \right] - \log \left[ {K_{{n{\text{-octanol}}/{\text{water}}}} } \right] \\ & = - 1.12 + 0.21R_{2} - 0.82\pi_{2}^{H} + 0.66\sum {\beta_{2}^{H} } - 0.5V_{x} . \\ \end{aligned}$$

Thus after allowing for the effect of the changes in n-octanol/water partition, there is much less variation to be explained and *the remaining effect of an increase in molecular volume is predicted to be a decrease in permeability*. (That this is the inverse square root relation predicted by the solubility-diffusion model is to some extent an “accident” of the choice of the preferred solvent for comparison, n-octanol. If olive oil had been chosen instead increases in volume would again be predicted to decrease permeability, but with a different more negative coefficient).

Fong [161] and Abraham [168, 171] provide discussions of which properties to use. Fong chooses desolvation from water, solvation in the membrane modelled by n-octanol, dipole moment and molecular volume. Molecular volume is negatively correlated.

## Appendix D. Kinetics of glucose transport across the blood–brain barrier

The concentration dependence of the rate of glucose influx, *T*_*inf*_, has often been described empirically using the simplest form of Michaelis–Menten kinetics [310]

28 $$T_{\inf } = {{T_{\text{max} } *c_{plasma} } \mathord{\left/ {\vphantom {{T_{\text{max} } *c_{plasma} } {\left( {K_{t} + c_{plasma} } \right)}}} \right. \kern-0pt} {\left( {K_{t} + c_{plasma} } \right)}}$$

where *T*_*max*_ is the maximum transport rate and *K*_*t*_ is the Michaelis constant for influx (*t* and *T* stand for “transport”). From this expression for the rate, the decrease in clearance with concentration is predicted to be

29 $$CL = {{T_{\text{max} } } \mathord{\left/ {\vphantom {{T_{\text{max} } } {\left( {K_{t} + c_{plasma} } \right)}}} \right. \kern-0pt} {\left( {K_{t} + c_{plasma} } \right)}}.$$

Betz et al. [327] reported *T*_*max*_ = 1.6 µmol g^−1^ min^−1^ and *K*_*t*_= 8.6 mM (based on blood rather than plasma) in the dog based on tracer uptake rate after 1 min of hypoxia, while Pardridge and Oldendorf [538] investigated transport of five different hexoses into rat brains and found *T*_*max*_ = 1.6 µmol g^−1^ min^−1^ for all, but differing apparent dissociation constants with 9 mM for glucose, which they took to imply that the conformation changes of the carrier rather than binding of the substrates were rate limiting. This is plausible because relatively low affinity binding of small substrates to sites is often diffusion controlled and rapid while conformation changes of large molecules may well be much slower. At a plasma concentration of 6 mM these values correspond to a glucose clearance of 100 µL g^−1^ min^−1^. Mason et al. [334] lists many values of *T*_*max*_ and *K*_*t*_ determined from flux studies prior to 1992. These range from 0.5 to 6.7 µmol g^−1^ min^−1^ for *T*_*max*_ and 4.9–11 mM for *K*_*t*_.

Efflux has not been measured directly, but it can be calculated by difference from the net flux and the influx. The net flux can be calculated from the rates of extraction of glucose from the blood as indicated in Sect. 5.3 (assuming negligible metabolism within the endothelial cells), or from the rates of glucose metabolism, perivascular loss and accumulation within the brain. Perivascular loss is likely to occur, but at a much lower rate than metabolism. At steady-state the rate of accumulation is zero and thus the net flux equals the rate of glucose metabolism,

30 $$T_{net} = CMR_{glc} .$$

The net flux and efflux have often been interpreted using the simplest extension of the Michaelis–Menten description used for influx, i.e. (see e.g. [310, 323, 539]) leading to

31 $$T_{eff} = \, T_{max} *{{c_{isf} } \mathord{\left/ {\vphantom {{c_{isf} } {\left( {K_{t} + \, c_{isf} } \right)}}} \right. \kern-0pt} {\left( {K_{t} + \, c_{isf} } \right)}}$$

and

32 $$T_{net} = T_{\inf } - T_{eff} = T_{max} \left( {{{c_{plasma} } \mathord{\left/ {\vphantom {{c_{plasma} } {\left( {K_{t} + c_{plasma} } \right)}}} \right. \kern-0pt} {\left( {K_{t} + c_{plasma} } \right)}} - {{c_{isf} } \mathord{\left/ {\vphantom {{c_{isf} } {\left( {K_{t} + \, c_{isf} } \right)}}} \right. \kern-0pt} {\left( {K_{t} + \, c_{isf} } \right)}}} \right).$$

These equations have been called irreversible Michaelis–Menten kinetics [540] because in Eqs. 31 and 32 the product of the “reaction”, which is the substrate on the far side of membrane after transport, has no effect on the rate of the reaction. This has been described as implying that influx and efflux occur by completely separate mechanisms, which was regarded as being most unlikely (see e.g. [338]). However, it should be noted that Eqs. 31 and 32 and even the extensions of these when two species are present are the same as equations that can be derived from the simple carrier model with the additional assumptions that association and dissociation are rapid, the rate constants for the conformation changes of the carrier are the same with or without a bound substrate and the mechanism is symmetrical, i.e the same viewed from either side [324].

The carrier model for kinetics was introduced to account for the transport of sugars across sheep placenta [323] and human red blood cells [324]. The name “carrier” arose because the model should apply when the transporter collects the substrate on one side of the membrane and “carries” it across the membrane to deposit it on the other. This appears to be the actual physical mechanism for ion transport by low molecular weight ion carriers like nonactin and trinactin ([530, 541, 542] and probably valinomycin [530, 543–545]. However for much larger transporters such as GLUT1, it has always been much more likely that the physical mechanism is somewhat different. The essential feature of carrier kinetics is not transfer of the carrier molecule across the membrane, but rather the change in exposure of the binding site for the substrate. It must be possible for this site to be exposed on each side of the membrane, but not both at the same time. The structures of GLUT1 (see Fig. 12) and related transporters all indicate that there is a transport pathway through the molecule which is occluded or gated at one end or the other and furthermore suggest conformation changes that could close the gate at one end while opening the gate at the other.

A second substrate can inhibit transport of the first by binding to the carriers thus reducing the number of carriers free to complex with the first. However, it is also possible for a second substrate to increase transfer of the first. In the extreme case if the carrier can only change conformation while a substrate is bound, the carrier is an obligatory exchanger and net transfer of one solute can only occur in the presence of another. More generally efflux of a substrate can, by increasing the rate of changes from inward to outward facing conformations increase the availability of carrier to collect a different substrate on the outside and hence its influx. Exchange whether or not obligatory can result in secondary active transport in which uphill transport of one solute is driven by downhill flux of another [322–324, 326]. Manifestations of this coupling are sometimes called variously counter-transport, counter-flow or trans-stimulation (see Fig. 12).

There are, of course, many extensions that can be made to the carrier model, examples include invoking more than one binding site, allowing co-transport and accounting for diffusion limited access in unstirred layers. Some form of extension has been found necessary for GLUT1 transport in red blood cells (see e.g. [546]) and almost certainly, given its greater complexity, will be necessary for glucose transport at the blood–brain barrier. The following is more an empirical description of results than a mechanistic model.

The general solution of the simple carrier model for the steady-state fluxes in terms of the concentrations and the constants of the model can be derived using standard methods for reaction kinetics [547–549]. This solution has been reported and discussed a number of times (see e.g. [326, 327, 339, 550] together with some possible extensions [329, 546].

The “simple” pore is another type of mechanism that has been considered for glucose transport across the blood–brain barrier. Pores can be gated but when the gates are open the transport pathway allows solute movement from one side of the membrane to the other with no further movement of the gates. Lieb and Stein [551] have described the kinetics for movements through “simple” pores that are defined as pores that can be occupied by only one substrate at a time. Generally, because the substrates are small and can move rapidly and no conformation changes of the pore are required, transport rates through an open pore can be large. By contrast for a simple carrier movement of each substrate molecule requires conformation changes, which are likely to be slow.

The simple pore mechanism does not predict or explain counter-transport or trans-stimulation. Reversible Michaelis–Menten kinetics (see [337, 338, 540, 552], which have been used in some descriptions of glucose transport at the blood–brain barrier, are the same as the kinetics of the simplest single occupancy pore [551].

As counter-transport is well established for GLUT1 in red blood cells and both counter-transport and trans-stimulation have been demonstrated for glucose transport at the blood–brain barrier, the simple pore model and reversible Michaelis–Menten kinetics are not considered further here (compare [339]).

Caution is advisable in the interpretation of flux data using the carrier model for at least three reasons: steady-state data cannot determine all of the rate constants even in the “simple” carrier model (see e.g. [326]); there must be transport across two membranes in series; and there is likely to be interaction between the GLUT1 monomers in the tetramers thought to be present in membranes. Thus it would be unwise to attach mechanistic significance to the values of constants determined by fitting the model to data. However, the forms of the equations relating the fluxes to the concentrations [325–327, 550] remain the simplest available framework capable of describing the transport. Using the constants that are defined below,

33 $$T_{inf} = T_{1,\text{max} } K_{2} \frac{{c_{plasma} \left( {1 + \frac{{c_{isf} }}{R}} \right)}}{{K_{1} K_{2} + K_{2} c_{plasma} + K_{1} c_{isf} + \frac{{c_{plasma} c_{isf} }}{{K_{inh} }}}}$$

34 $$T_{eff} = T_{2,\text{max} } K_{1} \frac{{c_{isf} \left( {1 + \frac{{c_{plasma} }}{R}} \right)}}{{K_{1} K_{2} + K_{2} c_{plasma} + K_{1} c_{isf} + \frac{{c_{plasma} c_{isf} }}{{K_{inh} }}}}$$

and

35 $$T_{net} = T_{1,\text{max} } K_{2} \frac{{c_{plasma} - c_{isf} }}{{K_{1} K_{2} + K_{2} c_{plasma} + K_{1} c_{isf} + \frac{{c_{plasma} c_{isf} }}{{K_{inh} }}}}$$

36 $${\text{with}}\quad \quad \quad \quad {{T_{{ 1,{ \text{max} }}} } \mathord{\left/ {\vphantom {{T_{{ 1,{ \text{max} }}} } {K_{ 1} }}} \right. \kern-0pt} {K_{ 1} }} = {{T_{{ 2,{ \text{max} }}} } \mathord{\left/ {\vphantom {{T_{{ 2,{ \text{max} }}} } {K_{ 2} }}} \right. \kern-0pt} {K_{ 2} }}.$$

In these equations *c*_*plasma*_ and *c*_*isf*_ are concentrations, the *K*s are apparent dissociation constants, and the *T*_max_ values are transport maxima for transport in the two directions. The inhibition constant, *K*_*inh*_, which is dimensionless, and the trans-stimulation constant, *R*, are related to the constants used by Betz et al. [327] by

37 $$K_{inh} = {{K_{1i} } \mathord{\left/ {\vphantom {{K_{1i} } {K_{1} }}} \right. \kern-0pt} {K_{1} }} = {{K_{2i} } \mathord{\left/ {\vphantom {{K_{2i} } {K_{2} }}} \right. \kern-0pt} {K_{2} }}$$

and

38 $$R = R_{1} = R_{2} .$$

1/*K*_*inh*_ indicates a combined effect of glucose in plasma and ISF to compete for transport and 1/R indicates the strength of trans-stimulation. An equation of the same form as Eq. 35 was used by Simpson et al. [315] to describe glucose transport in their modelling of cerebral energy metabolism. A symmetrical version was used by Duarte et al. [341] in their interpretation of data for the amount of glucose in the brain versus plasma concentration.

In the simple carrier model trans-stimulation can increase the unidirectional flux but not the net flux. For the unidirectional flux if conformation changes of the carrier are faster when substrate is bound, trans-stimulation is expected to be more important than the combined inhibition. Alternatively if the conformation changes are faster when substrate is not bound, trans-inhibition is expected to be more important.

If the transport were by the simple carrier model across a single membrane, the empirical constants could be calculated from the number of carriers and the rate constants of the model [325–327, 339, 550].

The investigation of glucose fluxes in the isolated, perfused dog brain by Betz et al. [327] appears to be the only study that allows calculation of rates of efflux for a range of values of both *c*_*plasma*_ and *c*_*isf*_. (They measured concentrations of glucose in whole blood, which are expected to be about 11% smaller than those in plasma. In the following the distinction has been ignored). In their study the steady-state values of *c*_*isf*_ were calculated as amount accumulated in the brain divided by the volume of distribution, *V*_*D*_ = milliliters of brain water per gram of brain; and the dependence of the rate of influx, *T*_*inf*_, on *c*_*plasma*_ was determined for a range of values of *c*_*isf*_ preset by perfusing the brains with different concentrations of glucose.

As shown in Fig. 14 over a wide range of glucose concentrations, from roughly 2 mM to 40 mM, at steady-state the amount in the brain and hence *c*_*isf*_ increases proportional to (*c*_*plasma*_ − offset) where the offset is about 2 mM.

Betz et al. [327] fitted their influx data using

39 $$T_{\inf } = {{T_{app} } \mathord{\left/ {\vphantom {{T_{app} } {\left( {K_{app} + c_{plasma} } \right)}}} \right. \kern-0pt} {\left( {K_{app} + c_{plasma} } \right)}}$$

with apparent values of the Michaelis–Menten constants shown in Table 8 for brains with *c*_*isf*_ set by pre-exposure to different *c*_*plasma*_. As *c*_*isf*_ is increased both the apparent transport maximum and the apparent Michaelis constants for influx increase which is evidence for both competition and trans-stimulation. Betz et al. interpreted the variation of the apparent constants with *c*_*isf*_ in terms of Eq. 33. As shown in the Additional file 1, all of the data for influx can be described empirically using Eq. 35. It is possible to calculate the rate of efflux, under steady-state conditions using *T*_*eff*_ = *T*_*inf*_ − *T*_*net*_ = *T*_*inf*_ − *CMR*_*glc*_ for the combinations of *c*_*plasma*_ and *c*_*isf*_ seen at steady-state. Furthermore, if influx is described empirically by Eq. 33 then Eq. 34 is expected to be a reasonable description of efflux over the same range of concentrations and thus can be used to calculate the efflux for all combinations of concentrations using the fitted empirical constants.

**Table 8:** Parameters obtained by Betz et al. [327] from fits of the simple Michaelis–Menten expression *T*_inf_ = *T*_*app*_/(*K*_*app*_ + *c*_*plasma*_) to glucose influx versus plasma concentration, *c*_*plasma*_, for preset concentrations of glucose in the brain, *c*_*isf*_

*c*_*isf*_/mM	6.11	16.8	26.3	43.9	56
*K*_*app*_/mM	8.46	11.2	17.7	28.2	37.7
*T*_*app*_/µmol g^−1^ min^−1^	1.61	1.84	2.21	2.68	3.83

The fits predict that for all *c*_*plasma*_, the net flux, *T*_*net*_ when *c*_*isf*_ is at the corresponding steady-state value is between 0.6 µmol g^−1^ min^−1^ and 0.65 µmol g^−1^ min^−1^. If it is demanded that the constants used to fit *T*_*inf*_ produce the same *T*_*net*_ for all steady-state conditions, i.e. that *CMR*_*glc*_, is constant, then the estimated value is 0.65 µmol g^−1^ min^−1^. This value is very close to the value expected for rats but somewhat greater than that expected for humans. The fits indicate that an adequate net flux can be maintained for *c*_*plasma*_ as low as about 3 mM. Increases in *c*_*plasma*_ produce relatively modest increases in influx with matching increases in efflux at steady-state such that the net flux remains constant. The corresponding increase in *c*_*isf*_ is shown in Fig. 14. A notable feature of the fits is that for *c*_*plasma*_ = 6 mM, if there were no change in transport capacity, glucose consumption, *CMR*_*glc*_, could increase only to about 0.9 µmol g^−1^ min^−1^. At that rate, *c*_*isf*_ would be close to 0. This limit on *CMR*_*glc*_ is substantially below the *T*_max_ value, which can be approached only if *c*_*plasma*_ is increased. As discussed in Sect. 6.2, how transport capacity is increased to support nervous activity is not fully understood.

Betz et al’s data show a pronounced trans-stimulation effect, but only for *c*_*plasma*_ > ~ 20 mM. For *c*_*plasma*_ < ~ 10 mM increasing *c*_*isf*_ decreases influx. In terms of the model this is expected because higher *c*_*isf*_ reduces the concentration of free carrier available to complex glucose from plasma.

## Appendix E. Blood–brain barrier permeabilities of Na^+^, K^+^ and Cl^−^

Determining the permeabilities of the blood–brain barrier for Na^+^, K^+^ and Cl^−^ was a major challenge because these ions are transferred between blood and the parenchyma by two routes, directly across the blood–brain barrier and indirectly via CSF. Davson and Welch [417] calculated permeabilities for the blood–brain barrier in rabbits by fitting data for accumulation of tracers in CSF and the parenchyma simultaneously using a simplified, but still complicated, model that allowed for transfers directly between blood and ISF, between blood and CSF and between CSF and ISF. While the model allowed the concentration of tracer in CSF to vary with time, it assumed that there was no variation with position, i.e. that the concentration was the same throughout the ventricles, cisterns and subarachnoid spaces. The model was based on equations that do allow the concentration to vary with position within the parenchyma, but no measurements of the variation were made. Davson and Welch’s approach suffers from the inevitable shortcomings associated with fitting a complicated model to limited data.

Using rats, Smith and Rapoport [419] took the more direct experimental approach of measuring the accumulation of tracer within the parenchyma at sites sufficiently far from the choroid plexuses, e.g. the frontal cortex, that, at least initially, entry had to be across the blood–brain barrier. They allowed accumulation to proceed for only 10 min which they reasoned was short enough that they could ignore both backflux from parenchyma to blood and indirect transfer from blood to CSF to parenchyma. One of the arguments that the permeabilities of the blood–brain barrier calculated by Davson and Welch and by Smith and Rapoport are at least reasonable approximations is that these two very different approaches yielded similar answers.

There is, however, an apparent difficulty with accepting the values calculated by Smith and Rapoport. Their analysis of the time course of the concentrations within the cortex started with their Eq. 1,

40 $$\frac{{dc_{br} \left( {x,t} \right)}}{dt} = \left( {PS} \right)c_{plasma} - \frac{{\left( {PS} \right)^{\prime } c_{br} \left( {x,t} \right)}}{{V_{br} }} + \frac{D}{{V_{br} }}\left[ {\frac{{d^{2} c_{br} \left( {x,t} \right)}}{{dx^{2} }}} \right],$$

which is dimensionally inconsistent. In this equation *c*_*br*_(*x*,*t*) is the concentration of tracer in the parenchyma, units dpm g^−1^; *P* is the permeability of the blood–brain barrier, units cm s^−1^; *S* is the area of the blood–brain barrier, units cm^2^ g^−1^; *c*_*plasma*_ is the concentration in plasma, units dpm cm^−3^; *V*_*br*_ is the volume of distribution of the tracer substance, units cm^3^ g^−1^; *D* is the diffusion constant of the tracer in the extracellular fluid, units cm^2^ s^−1^, *x* is distance from the ventricular surface, units cm; and *t* is the time, units s. The units of the first two terms on the right hand side, those which describe blood–brain barrier transport, are dpm s^−1^ g^−1^, which is the same as for the left hand side, but the units of the third term, which describes diffusion within the cortex, are dpm s^−1^ cm^−3^. Terms with different units cannot be added together, thus this equation cannot be correct.

On closer inspection of Eq. 40 and the model on which it is based, the conversion factor in the third term, *V*_*br*_, is wrong (if the substance is restricted to the extracellular space, *V*_*br*_ should simply be omitted [553]) but in addition there are more fundamental difficulties. The model is based on at least two unstated assumptions that limit its use: it is assumed that the only movement of ions through the cortex is via diffusion in the extracellular space and that there is no exchange of substance with CSF in the sub-arachnoid spaces. (The first of these shortcomings also compromises the analysis by Davson and Welch [417]). At least for K^+^ it is clear that movements within cell processes make an important contribution to movements within the cortex, so called spatial buffering (see e.g. [554–556]). As can be seen from Gardner-Medwin’s papers, if the tracer can enter and leave cells on the time scale of the experiments a proper description of the third term on the right hand side of Eq. 40 would be very complicated. The second assumption becomes important if perivascular clearance is comparable to the clearance across the blood–brain barrier (see end of this Appendix).

An immediate consequence of the use of Smith and Rapoport’s starting equation is that calculation of the concentrations within the parenchyma cannot be relied upon whenever these concentrations vary with position, as in their Fig. 6, unless the substance in question cannot enter the cells and all positions considered are far from perivascular spaces and the brain surfaces.

Fortunately, Smith and Rapoport designed their experiments in such a way that the calculation of the transfer constants and permeabilities for the blood–brain barrier does not depend on how the model describes diffusion within the parenchyma. Their Eq. 4 for the transfer constant, taken from Fenstermacher and Rapoport [159] yields constants with units cm^3^ s^−1^ g^−1^. Their actual calculations leading to the values in their Table 2 were equivalent to using an equation,

41 $$k_{br} = \frac{{c_{br} \left( T \right)/\bar{V}_{brain} }}{{\int\limits_{0}^{T} {c_{plasma} dt} }},$$

which incorporates a conversion factor between the mass and volume of the brain, $\bar{V}_{brain}$ assumed to be 1 cm^3^ g^−1^. In this equation the units of *k*_*br*_ are s^−1^; *c*_*br*_(*T*), units dpm g^−1^, is the total concentration per gram of tissue; *T*, units s, is the period of time during which influx occurs; and *c*_*plasma*_ is the concentration in plasma, units dpm cm^−3^. Smith and Rapoport assumed that *c*_*plasma*_ was constant so that the integral becomes the product *c*_*plasma*_ × *T* and

42 $$k_{br} = \frac{{c_{br} \left( T \right)}}{{c_{plasma} T\bar{V}_{brain} }}.$$

The rate of change of the concentration within the brain can be related to the permeability and area of the blood–brain barrier using

43 $$\frac{{dc_{br} \left( t \right)}}{dt} = \left( {PS} \right)c_{plasma}$$

which, using the same assumptions needed for Eq. 42, integrates to

44 $$c_{br} \left( T \right) = \left( {PS} \right)c_{plasma} T.$$

Combining Eqs. 42 and 44

45 $$P = \frac{{c_{br} \left( T \right)}}{{c_{plasma} TS}} = \frac{{k_{br} \bar{V}_{brain} }}{S}.$$

Smith and Rapoport used *S* = 140 cm^2^ g^−1^ from [557], and a tissue volume per gram, $\bar{V}_{brain}$ = 1 cm^3^ g^−1^, leading to the values of *P* (in cm s^−1^) quoted in their Table 3. (Eq. 44 shows that the *PS* product, which is the estimate of the clearance, does not depend on the values assumed for either $\bar{V}_{brain}$ or *S*. For Na^+^ *PS* was 2 × 10^−5^ cm^3^ s^−1^ g^−1^ = 1.2 µL min^−1^ g^−1^. This is similar to estimates of perivascular clearance, ~ 1 µL min^−1^ g^−1^ (see Sect. 3.2).

The *PS* product for K^+^ was [419] 11.3 µL min^−1^ g^−1^. This larger value is based on fluxes that were somewhat smaller than those for Na^+^ but at substantially smaller concentrations, e.g. 4 mM rather than 140 mM. Unlike those for Na^+^, the fluxes for K^+^ can be substantially reduced by inhibitors of transporters known to be present in the endothelial cells (reviewed in [4]).

The model used by Smith and Rapoport ignores exchange of substance between the parenchyma and CSF in the sub-arachnoid spaces, i.e. it ignores perivascular transport. However, for the same reason that backflux from parenchyma to blood does not alter the initial rate of increase in concentration within the parenchyma, loss to CSF will also not alter the initial rate and thus the calculation of *PS*. However, once concentrations in parenchyma and CSF increase, net perivascular fluxes for Na^+^ will be comparable to the net fluxes across the blood–brain barrier and thus cannot be ignored in calculations of the time course.

## Footnotes

1 The limitation of movements from cisterna magna towards the IIIrd ventricle occurs presumably because the volume displaced in the to and fro fluid movements through the cerebral aqueduct [7, 602] is too small for efficient transfer of solutes. More movement occurs in the opposite direction as a consequence of net flow. However, Vartan Kurtcuoglu (personal communication) has pointed out that simulation of the convective mixing in the IIIrd ventricle and aqueduct indicates that the transient jet of fluid entering the IIIrd ventricle from the aqueduct in each cardiac cycle (see the sub-figures for 0.2 T and 0.3 T in Figure 8 of [8]) is as long as the aqueduct itself implying that some transfer from the IVth to the IIIrd ventricle should occur. The data reported by Ringstad et al. [15] indicate that gadobutrol added to lumbar CSF does reach the IIIrd ventricle in control patients, but at a low concentration, while the concentration seen in patients with idiopathic normal pressure hydrocephalus is substantially higher. This is consistent with the view that the net flow through the aqueduct is normally from IIIrd to IVth ventricle but in communicating hydrocephalus it is reversed in direction (for references, review and discussion see sections 4.2.2–4.2.5 in [41]).

2 The effects of diffusion across the ependyma lining the ventricles are usually restricted to regions close to the ventricles [21, 25, 603–605]. However, in the presence of oedema or in the immediate aftermath of infusion of even quite small amounts of fluid into the parenchyma [129] flow across the ependymal layer lining the ventricles can be substantial (see e.g. [129, 606, 607]). Rosenberg et al. [65] investigated the penetration of sucrose from the ventricles into the parenchyma during ventriculo-cisternal perfusions. In grey matter they found concentration profiles in the parenchyma consistent with simple diffusion. However, in white matter the profile was altered as if there were a 10 µm min^−1^ flow of ISF towards the ventricles that countered diffusion into the tissue. Flow at this velocity could move solutes as far as a millimetre in 100 min.

3 The relative lack of consideration of the choroid plexuses is not meant to suggest that the only important function of the choroid plexuses is to secrete CSF. The choroid plexuses are the "obvious" best route for transfer to or from the blood for anything that is intended to act on regions of the brain close to the surfaces of the ventricles or that is produced in those regions for actions elsewhere in the body. They must also be considered for transfer into the brain of anything that is needed neither quickly nor in large quantity such as Ca2^+^, Mg^2+^, micronutrients and some hormones. Finally they should be considered as a potential pathway for drug delivery to the brain via CSF [36].

4 While the tight junctions have a very low permeability to Na^+^ and Cl^−^, the passive influx or efflux of these across the blood–brain barrier may still be primarily paracellular as proposed by Crone [151]. However the passive influxes are almost equal to the passive effluxes and the net fluxes for Na^+^ and Cl^−^ across the endothelial layer may be determined primarily by the mechanisms for transfer through the cells (see Section 5.6 and Sections. 4.3.4 and 4.3.5 of [4] for further discussion).

5 It is clear that extramural, fluid-filled perivascular spaces can exist, because large particles can be introduced into them (see e.g. [70, 95] and fluorescent tracers injected into the cisterna magna are seen in regions extending well outside the vessel walls (see e.g. Figures 2 and 3 in [25] and Figure 2 in [96]). Furthermore, after subarachnoid haemorrhage blood can accumulate between the walls of arteries and the glial endfeet [92]. The question is whether normally the spaces are inflated with fluid or collapsed virtual spaces [53]. A need for "inflation" would provide a ready explanation for why altering CSF pressures, e.g. by puncture of the cisterna magna [608], can greatly reduce perivascular influx. In fixed, sectioned tissue of gray matter inflated spaces are rarely if ever seen [98, 99]. However, spaces particularly, one imagines, labile spaces, are difficult to fix, or as Coles et al. [1] put it "in fixed tissue, extracellular spaces tend to be occluded, and marker molecules are bound to host tissue". It is not clear that this type of evidence obtained with fixed material is a valid description of perivascular spaces in vivo.

6 A study by Bradbury et al. [82] comparing the distribution of radio-iodinated serum albumin (RISA) in rabbits after intraparenchymal injection with that after intraventricular injection remains one of the most informative undertaken on the routes of elimination of large solutes. Their finding of RISA in the walls of arteries in the circle of Willis after intraparenchymal injection provides strong evidence that some of the albumin leaves the parenchyma along or within arterial walls and continues along them beyond the subarachnoid spaces. This evidence does not, however, indicate how large a fraction of the RISA takes this route. Bradbury et al. also observed that after intraparenchymal injection a smaller fraction of the amount injected passed through the cisterna magna and a larger fraction reached lymph than was evident after intraventricular injection. The RISA distribution following intraventricular injection can be interpreted as tracing the routes followed by CSF emerging from the ventricles. After mixing and passing through the cisterna magna, the CSF flows out of the brain by more than one route, some via the arachnoid villi leading to venous sinuses and some via the cribriform plate leading to the nasal mucosa. From the nasal mucosa large solutes including RISA pass into lymph. After intraventricular injection, the fraction of RISA reaching lymph is somewhat greater than half and represents that fraction of CSF from the ventricles that flows out via the cribriform plate [82]. After intraparenchymal injection, the fraction of RISA reaching lymph is substantially larger than that seen after intraventricular injection. This observation was interpreted by Bradbury et al. [82] as indicating that most of the RISA contained in ISF flowed out of the parenchyma into a portion of CSF that subsequently left the brain via the cribriform plate to the nasal mucosa rather than via the arachnoid villi. Weller, Carare and associates [95, 98, 107], who have used mice for their functional experiments, have since favoured the view that outflow of ISF from the parenchyma occurs via a route that does not entail mixing with any portion of the CSF. In their view ISF drains from the parenchyma along intramural periarterial pathways and exchange of large solutes is not possible between these pathways and CSF as the vessels pass through the subarachnoid spaces or basal cisternae.

7 In terms of resisting putative water flow from periarterial to perivenular spaces, the endfeet are in parallel with the gaps between them and in series with the parenchymal tissue. Jin et al. conclude that the overall calculated resistance to flow is similar with or without a water permeability of the endfeet—i.e. the permeability of the slits is sufficient for the overall resistance to be determined largely by that of the parenchymal tissue.

8 The macromolecular components of ISF may greatly influence the resistance to bulk flow as emphasized for peripheral extracellular fluid by Guyton and associates [609]. Quantitatively macromolecules in peripheral extracellular fluid can increase resistance to flow by orders of magnitude [138, 610, 611] while having much less effect on diffusion of small molecules [76, 81, 612]. A well-known illustration of a closely related effect is the reduced flow with maintained diffusion when agar is added to solutions.

9 Charles Nicholson and Anthony Gardner-Medwin (personal communications reported in [41]) have observed that the adequacy of diffusion to explain movements of solutes in the interstitium and the observed symmetrical spread does not preclude the existence of net flows of the order of those proposed by Cserr and coworkers [83, 130] or Rosenberg et al. [65]. It is not clear whether the flows envisaged in the glymphatic hypothesis are sufficiently larger that they should produce observable asymmetry in the spread of markers. Comparison of the flow required if glymphatic circulation accounts for the movement of markers like inulin through the perivascular spaces (0.6 µL g^−1^ min^−1^ or more as discussed above) and the largest flow that could have been missed by Smith et al. [79] would be very welcome.

10 Compared to the change in rate of efflux of inulin, Xie et al. [128] observed a much larger change in the rate of delivery of Texas Red dextran from the cisterna magna to the parenchyma: 20-fold less influx when the mice were awake compared to when they were anaesthetized. Benveniste et al. [613] have also observed increases in delivery of a gadolinium chelate with anaesthesia. However, Gakuba et al. [147] using another gadolinium chelate, Evan's blue and indocyanine green have observed that anaesthesia greatly reduces the spread of the markers into the parenchyma from the cisterna magna. It is not yet clear how to reconcile these results (see also [426]). It is also unclear whether the effects of sleep/wakefulness/anaesthesia on influx of markers are mediated primarily in the subarachnoid spaces, in the perivascular spaces or within the parenchyma (see Section 2.4 in [146]).

11 The polar headgroups of lipid membranes produce a large dipole potential (membrane core positive) which favours permeation of anions over cations (see e.g. [529, 614–619]. None of the descriptors that are suitable for describing neutral molecules can be expected to allow the LFER approach to be able to cope with this difference. It is thus not at all surprising that when Abraham came to consider charged molecules explicitly, he found it necessary to introduce descriptors that allow for the charge on the molecule [168]. One difficulty encountered when attempting to correlate permeability and lipophilicity for ions is that it is only possible to measure *K*_*n-octanol*/water_ for neutral combinations of ions. This is an example of the consequences of the Principle of electroneutrality (see Section 6.1.2 in [4] for another example and further discussion). This difficulty could be avoided by measuring partition into unilamellar liposomes, lipid bilayers or biological membranes rather than into a hydrophobic solvent, because with these systems the counterions can remain in the aqueous phases. However, while partition into membranes has been measured, there has not been any attempt to correlate these measurements with blood–brain barrier permeability (see e.g. [161]).

12 Both the Oat and Oatp transporters appear to be exchangers. Using Xenopus oocytes transfected with Oat3, influx of labelled p-aminohippuric acid (PAH) or estrone sulphate was found to be coupled in some way to movement of glutarate, and probably other dicarboxylates, in the opposite direction, i.e. there was trans-stimulation of transport [620]. Interestingly however, influx of labelled estrone sulphate, was not stimulated by increased internal concentration of estrone sulphate, or PAH, i.e. there was no "self" trans-stimulation [236]. For Oatp transporters the exchange has different properties. For instance for Oatp1a4 (Oatp2) expressed in Xenopus oocytes suspended in low bicarbonate solution, increased concentrations of a variety of solutes present inside the cells, including taurocholate, glutathione, and glutathione conjugates, stimulate influx of labelled taurocholate [621]. However, when Oatp1a4 is expressed in a HeLa cell line suspended in bicarbonate buffered solution, the influx of taurocholate seems to be coupled to efflux of bicarbonate [622]. This coupling with bicarbonate has been confirmed using a number of different Oatp transporters expressed in CHO cells [623]. Exchangers are able to perform secondary active transport by coupling the downhill transport of one solute to the uphill movement of the other. Thus the demonstration of uphill transport from brain to blood might correspond to abluminal secondary active transport into the endothelial cells driven by an outward gradient of something like glutarate or glutathione or to luminal primary active transport out of the endothelial cells via an ABC transporter or to both. For instance PAH may be taken up into the cells by secondary active transport via Oat3 and subsequently expelled from them by primary active transport via an ABC transporter, possibly MRP4 [560]. Further work is required to establish the interplay of the effects of the various transporters.

13 TfR has been shown to be accessible to antibodies on both surfaces of the endothelial cells [280, 624]. This suggests that transferrin could bind to or dissociate from TfR on either side. Furthermore there is effective transfer across the blood–brain barrier of low-affinity antibodies to TfR [625], which strongly suggests that there can be transcytosis of substances bound to the TfR. In terms of one current conceptual model (see Figure 1 in [280]) there are two possible fates of transferrin and iron after endocytosis. In the first fate, holo-transferrin is exocytosed across the abluminal membrane as in the original hypothesis. However the current consensus is that relatively little holo-transferrin is in fact exocytosed on the brain side [262, 279–282, 626–628]. In the second fate, dissociation of the holo-transferrin occurs within endosomes in the endothelial cells with the iron being transported across the abluminal membrane by ferroportin. In this option, one that is preferred by Simpson et al. [280], the iron-free apo-transferrin is exocytosed, part across the abluminal membrane accounting for the observed transfer of transferrin from blood to brain but primarily across the luminal membrane back to blood. This model allows the possibility that transcytosis of apo-transferrin can occur from brain to blood. The site of dissociation of the iron from the holo-transferrin inside the endothelial cell layer and the intervening steps are still under active investigation [281, 282]).

14 Simpson et al. [315] have calculated the distribution of glucose using estimates of the rates of metabolism and the rates of transport across the various barriers in the brain including diffusion through basal laminae and interstitial spaces and transport across the various cell membranes. Their calculated concentrations in ISF 1.4 mM, in neurons 1.2 mM and in astrocytes 0.9 mM are consistent with the relatively uniform distribution of glucose over brain water found by NMR.

15 There has been a claim that "The glymphatic [perivascular] pathway is important for the brain-wide delivery of nutrients, specifically glucose" [109]. This was based on results presented by Lundgaard et al. [629] for movements of a near-infrared 2-deoxyglucose probe (2DG-IR). However, Lundgaard et al. showed that 2DG-IR could not be delivered across the blood–brain barrier and thus it is at best a poor substrate for GLUT1. Since GLUT1 is essential for the normal entry and distribution of glucose, the results for 2DG-IR cannot be used to infer the relative importance of the blood–brain barrier and perivascular routes for the distribution of glucose. Petit and Magistretti [344] have also criticized the use of 2DG-IR as a probe for glucose movements into astrocytes and neurons.

16 Lundgaard et al. [630] have shown that four different manoeuvres that decrease perivascular efflux of markers increase lactate levels in the brain and decrease them in the submandibular and parotid lymph nodes. In their view some lactate leaves the brain in CSF notably via the cribriform plate to the nasal mucosa from which it is removed in lymph. There may be removal of lactate from the brain via lymph, but Bradbury and Westrop [125] noted that while high molecular weight markers like albumin delivered to the nasal mucosa are removed from the mucosa by lymph, low molecular weight substances like lactate may be removed from the mucosa by the peripheral blood flow. Lundgaard et al's results provide no means to verify that the lactate found in the lymph nodes originated in the brain and even if the lactate in the glands originates by perivascular efflux from the brain, they do not quantify the rate of efflux. The effects of sleep and wakefulness on lactate clearance from the brain were considered further in [146]. Lundgaard et al's results indicate that the manoeuvres that affect perivascular efflux do not alter lactate concentrations in the brain of usually awake mice (dark phase of 24 h cycle), which is evidence that perivascular efflux is not important under just the circumstances when there is likely to be a need for lactate removal.

17 The concentrations in plasma and CSF have been measured in samples of the fluids, but those in ISF have been measured using microdialysis. In the microdialysis procedure a probe is inserted and fluid perfused through the probe. To avoid grossly disturbing the ISF around the probe, the composition of the perfusion fluid must be close to that of ISF. The perfusate comes into contact with ISF only through a dialysis membrane. The diffusable solutes to be measured enter the perfusate during the relatively brief time that it is within the probe, and thus the slower the perfusion rate, the closer the concentration emerging from the probe is to the concentration in ISF in the region surrounding it. The ISF data in Table 3 were obtained by measuring concentrations at several different flow rates and extrapolating back to zero flow. However, even with these precautions, without measurements for substances whose concentrations are already known it is difficult to be certain that the ISF concentration measured is the same as that in ISF that isn't close to the probe. Because in all the studies in Table 3 the probe removes the substance being measured, there is an obvious risk of bias towards values that are too low. Evidence that these concerns are not just theoretical is provided by measurements for glucose. The early microdialysis measurements yielded values, e.g. 0.47 mM [631] or 0.35 mM [632], that are substantially smaller than the lower limit of ISF concentration obtained from NMR data, ca 1.2 mM for 6 mM in plasma (see Sect. 5.3). More recent microdialysis measurements have yielded larger concentrations [314], e.g 1 mM [633]; 1.66, [634]; 1.26 [635] and 1.4 [636].

18 The results were later extended to allow calculation of Michaelis–Menten constants for each of the amino acids [372]. For the large neutral amino acids the *V*_*max*_ and *K*_*m*_ values varied from 30 nmol g^−1^ min^−1^ and 0.12 mM for phenylalanine to 49 nmol g^−1^ min^−1^ and 0.63 mM for valine. Competition reduces the fluxes seen for each amino acid but for any one transporter may have a *V*_*max*_ for the combination of amino acids present somewhere in the range observed for the individual amino acids. Thus from these results from the rat studies the maximum collective influx of the large neutral amino acids is expected to be less than 50 nmol g^−1^ min^−1^. Smith and Stoll [43] provide a useful table of influxes from nearly normal composition of plasma with a total influx of 72 nmol g^−1^ min^−1^. It should be noted that (a) this total is for the influx not the net flux and (b) virtually all of the data for tracer influxes have been obtained with rats.

19 It should be noted that the total net flux of N as part of amino acids is not zero in any of the studies measuring net fluxes. The estimates for studies that included glutamine and at least 10 other amino acids are + 26 nmol g^−1^ min^−1^ [588]; − 17.3 nmol g^−1^ min^−1^ [589] and − 30 nmol g^−1^ min^−1^ [590]. Eriksson et al. [588] noted that their data could not be reconciled with N balance. The net effluxes found by Grill et al. [589] and Strauss et al. [590] were dominated by net release of glutamine, which conceivably could be balanced by small net influxes for many amino acids, each below the limit of detection, or a net influx of NH_4_^+^ though there is no evidence for this [359]. How N balance is achieved remains to be clarified.

20 This may explain the only modest success of attempts to understand the effects of raised concentration of one amino acid in plasma on the fluxes and ISF concentrations of other amino acids. Those attempts have considered only the concentrations in plasma while it is now clear that concentrations on both sides are important. (See [42] for discussion of the early work and [637] for a more recent example).

21 The results of Kress et al. [514] differ from the earlier studies described above. Kress et al. have reported the fractions of Aβ and inulin remaining in the brain 1 h after parenchymal injection in young, middle aged, or old mice. In each group they found that the fraction remaining of Aβ was smaller than that of inulin but the difference between Aβ and inulin was much less marked than in the earlier studies primarily because more Aβ remained in the brain. It is difficult to suggest any reason for this discrepancy.

22 In a number of studies (see e.g. [341]) the net flux during low nervous activity has been compared to an estimate of the transport maximum, *T*_max_. *T*_max_ is typically two to threefold larger. However, *T*_max_ gives a false impression of the transport reserve as these large fluxes can only be reached by increasing *c*_*plasma*_ to levels that are never achieved.

23 ("Systems" have functional definitions and may be mediated by one or more transporters. Transporters are gene products).

## References

[1] Coles JA, Myburgh E, Brewer JM, McMenamin PG (2017). Where are we? The anatomy of the murine cortical meninges revisited for intravital imaging, immunology, and clearance of waste from the brain. Prog Neurobiol.

[2] Damkier HH, Brown PD, Praetorius J (2013). Cerebrospinal fluid secretion by the choroid plexus. Physiol Rev.

[3] Spector R, Keep RF, Snodgrass SR, Smith QR, Johanson CE (2015). A balanced view of choroid plexus structure and function: focus on adult humans. Exp Neurol.

[4] Hladky SB, Barrand MA (2016). Fluid and ion transfer across the blood–brain and blood–cerebrospinal fluid barriers; a comparative account of mechanisms and roles. Fluids Barriers CNS.

[5] Chen L, Beckett A, Verma A, Feinberg DA (2015). Dynamics of respiratory and cardiac CSF motion revealed with real-time simultaneous multi-slice EPI velocity phase contrast imaging. Neuroimage.

[6] Yildiz S, Thyagaraj S, Jin N, Zhong X, Heidari Pahlavian S, Martin BA, Loth F, Oshinski J, Sabra KG (2017). Quantifying the influence of respiration and cardiac pulsations on cerebrospinal fluid dynamics using real-time phase-contrast MRI. J Magn Reson Imaging.

[7] Takizawa K, Matsumae M, Sunohara S, Yatsushiro S, Kuroda K (2017). Characterization of cardiac- and respiratory-driven cerebrospinal fluid motion based on asynchronous phase-contrast magnetic resonance imaging in volunteers. Fluids Barriers CNS.

[8] Kurtcuoglu V, Soellinger M, Summers P, Poulikakos D, Boesiger P (2007). Mixing and modes of mass transfer in the third cerebral ventricle: a computational analysis. J Biomech Eng.

[9] Yamada S, Kelly EJ (2016). Cerebrospinal fluid dynamics and the pathophysiology of hydrocephalus: new concepts. Semin Ultrasound CT MRI.

[10] Strecker EP, James AE (1973). Evaluation of cerebrospinal-fluid flow and absorption - clinical and experimental studies. Neuroradiology.

[11] Proescholdt MG, Hutto B, Brady LS, Herkenham M (2000). Studies of cerebrospinal fluid flow and penetration into brain following lateral ventricle and cisterna magna injections of the tracer [C-14]inulin in rat. Neuroscience.

[12] Vladic A, Klarica M, Bulat M (2009). Dynamics of distribution of H-3-inulin between the cerebrospinal fluid compartments. Brain Res.

[13] Iliff JJ, Wang MH, Zeppenfeld DM, Venkataraman A, Plog BA, Liao YH, Deane R, Nedergaard M (2013). Cerebral arterial pulsation drives paravascular CSF-interstitial fluid exchange in the murine brain. J Neurosci.

[14] Yamada S, Tsuchiya K, Bradley WG, Law M, Winkler ML, Borzage MT, Miyazaki M, Kelly EJ, McComb JG (2015). Current and emerging MR imaging techniques for the diagnosis and management of CSF flow disorders: a review of phase-contrast and time-spatial labeling inversion pulse. AJNR Am J Neuroradiol.

[15] Ringstad G, Vatnehol SAS, Eide PK (2017). Glymphatic MRI in idiopathic normal pressure hydrocephalus. Brain.

[16] Pizzo ME, Wolak DJ, Kumar NN, Brunette E, Brunnquell CL, Hannocks M-J, Abbott NJ, Meyerand ME, Sorokin L, Stanimirovic DB, Thorne RG (2018). Intrathecal antibody distribution in the rat brain: surface diffusion, perivascular transport, and osmotic enhancement of delivery. J Physiol (Lond).

[17] Rall DP, Lajtha A, Ford DH (1968). Transport through the ependymal linings. Progress brain research.

[18] Brightman MW, Reese TS (1969). Junctions between intimately apposed cell membranes in the vertebrate brain. J Cell Biol.

[19] Brightman MW, Klatzo I, Olsson Y, Reese TS (1970). The blood–brain barrier to proteins under normal and pathological conditions. J Neurol Sci.

[20] Strazielle N, Ghersi-Egea JF (2013). Physiology of blood–brain interfaces in relation to brain disposition of small compounds and macromolecules. Mol Pharm.

[21] Rall DP, Oppelt WW, Patlak CS (1962). Extracellular space of brain as determined by diffusion of inulin from the ventricular system. Life Sci.

[22] Fenstermacher J, Kaye T (1988). Drug diffusion within the brain. Ann NY Acad Sci.

[23] Nagaraja TN, Patel P, Gorski M, Gorevic PD, Patlak CS, Fenstermacher JD (2005). In normal rat, intraventricularly administered insulin-like growth factor-1 is rapidly cleared from CSF with limited distribution into brain. Cerebrospinal Fluid Res.

[24] Sykova E, Nicholson C (2008). Diffusion in brain extracellular space. Physiol Rev.

[25] Iliff JJ, Wang M, Liao Y, Plogg BA, Peng W, Gundersen GA, Benveniste H, Vates GE, Deane R, Goldman SA (2012). A paravascular pathway facilitates CSF flow through the brain parenchyma and the clearance of interstitial solutes, including amyloid β. Sci Transl Med.

[26] Cserr HF (1971). Physiology of choroid plexus. Physiol Rev.

[27] Redzic ZB, Segal MB (2004). The structure of the choroid plexus and the physiology of the choroid plexus epithelium. Adv Drug Deliv Rev.

[28] Strazielle N, Khuth ST, Ghersi-Egea JF (2004). Detoxification systems, passive and specific transport for drugs at the blood-CSF barrier in normal and pathological situations. Adv Drug Deliv Rev.

[29] Zheng W, Chodobski A (2005). The blood-cerebrospinal fluid barrier.

[30] Spector R (2009). Nutrient transport systems in brain: 40 years of progress. J Neurochem.

[31] Redzic Z (2011). Molecular biology of the blood–brain and the blood-cerebrospinal fluid barriers: similarities and differences. Fluids Barriers CNS.

[32] Liddelow SA, Dziegielewska KM, Ek CJ, Habgood MD, Bauer H, Bauer HC, Lindsay H, Wakefield MJ, Strazielle N, Kratzer I (2013). Mechanisms that determine the internal environment of the developing brain: a transcriptomic, functional and ultrastructural approach. PLoS ONE.

[33] Redzic ZB (2013). Studies on the human choroid plexus in vitro. Fluids Barriers CNS.

[34] Spector R, Snodgrass SR, Johanson CE (2015). A balanced view of the cerebrospinal fluid composition and functions: focus on adult humans. Exp Neurol.

[35] Liddelow SA (2015). Development of the choroid plexus and blood-CSF barrier. Front Neurosci.

[36] Strazielle N, Ghersi-Egea J-F (2016). Potential pathways for CNS drug delivery across the blood-cerebrospinal fluid barrier. Curr Pharm Des.

[37] Liddelow SA, Dziegielewska KM, Ek CJ, Habgood MD, Bauer H, Bauer H-C, Lindsay H, Wakefield MJ, Strazielle N, Kratzer I (2016). Correction: mechanisms that determine the internal environment of the developing brain: a transcriptomic, functional and ultrastructural approach. PLoS ONE.

[38] Praetorius J, Damkier HH (2017). Transport across the choroid plexus epithelium. Am J Physiol.

[39] Simon MJ, Iliff JJ (2016). Regulation of cerebrospinal fluid (CSF) flow in neurodegenerative, neurovascular and neuroinflammatory disease. Biochim Biophys Acta.

[40] Abbott NJ, Pizzo ME, Preston JE, Janigro D, Thorne RG (2018). The role of brain barriers in fluid movement in the CNS: is there a ‘glymphatic’ system?. Acta Neuropathol.

[41] Hladky SB, Barrand MA (2014). Mechanisms of fluid movement into, through and out of the brain: evaluation of the evidence. Fluids Barriers CNS.

[42] Pardridge WM (1998). Blood–brain barrier carrier-mediated transport and brain metabolism of amino acids. Neurochem Res.

[43] Smith QR, Stoll J, Pardridge WM (1998). Bood-brain barrier amino acid transport. Introduction to the blood–brain barrier methodology, biology and pathology.

[44] Hawkins RA, O’Kane RL, Simpson IA, Viña JR (2006). Structure of the blood–brain barrier and its role in the transport of amino acids. J Nutr.

[45] Engelhardt B, Sorokin L (2009). The blood–brain and the blood-cerebrospinal fluid barriers: function and dysfunction. Semin Immunopathol.

[46] Abbott NJ, Patabendige AAK, Dolman DEM, Yusof SR, Begley DJ (2010). Structure and function of the blood–brain barrier. Neurobiol Dis.

[47] Abbott NJ (2013). Blood–brain barrier structure and function and the challenges for CNS drug delivery. J Inherit Metab Dis.

[48] de Lange EC, Hammarlund-Udenaes M (2015). Translational aspects of blood–brain barrier transport and central nervous system effects of drugs: from discovery to patients. Clin Pharmacol Ther.

[49] Banks WA (2016). From blood–brain barrier to blood–brain interface: new opportunities for CNS drug delivery. Nat Rev Drug Discov.

[50] Summerfield SG, Zhang Y, Liu H (2016). Examining the uptake of central nervous system drugs and candidates across the blood–brain barrier. J Pharmacol Exp Ther.

[51] Pollak TA, Drndarski S, Stone JM, David AS, McGuire P, Abbott NJ (2017). The blood–brain barrier in psychosis. Lancet Psychiatry.

[52] Tarasoff-Conway JM, Carare RO, Osorio RS, Glodzik L, Butler T, Fieremans E, Axel L, Rusinek H, Nicholson C, Zlokovic BV (2015). Clearance systems in the brain-implications for Alzheimer disease. Nat Rev Neurol.

[53] Brinker T, Stopa EG, Morrison J, Klinge PM (2014). A new look at cerebrospinal fluid circulation. Fluids Barriers CNS.

[54] Benveniste H, Liu X, Koundal S, Sanggaard S, Lee H, Wardlaw JM (2018). The glymphatic system and waste clearance with brain aging: a review. Gerontology.

[55] Bradbury MWB (1979). The concept of a blood–brain barrier.

[56] Davson H, Segal MB (1996). Physiology of the CSF and blood–brain barriers.

[57] Campos-Bedolla P, Walter FR, Veszelka S, Deli MA (2014). Role of the blood–brain barrier in the nutrition of the central nervous system. Arch Med Res.

[58] Kubo Y, Ohtsuki S, Uchida Y, Terasaki T (2015). Quantitative determination of luminal and abluminal membrane distributions of transporters in porcine brain capillaries by plasma membrane fractionation and quantitative targeted proteomics. J Pharm Sci.

[59] Duffy KR, Pardridge WM (1987). Blood–brain-barrier transcytosis of insulin in developing rabbits. Brain Res.

[60] Banks WA, Kastin AJ, Fasold MB, Barrera CM, Augereau G (1988). Studies of the slow bidirectional transport of iron and transferrin across the blood–brain barrier. Brain Res Bull.

[61] Zhang Y, Pardridge WM (2001). Rapid transferrin efflux from brain to blood across the blood–brain barrier. J Neurochem.

[62] Shibata M, Yamada S, Kumar SR, Calero M, Bading J, Frangione B, Holtzman DM, Miller CA, Strickland DK, Ghiso J, Zlokovic BV (2000). Clearance of Alzheimer’s amyloid-β(1-40) peptide from brain by LDL receptor-related protein-1 at the blood–brain barrier. J Clin Invest.

[63] Preston JE, Abbott NJ, Begley DJ, Davis TP (2014). Transcytosis of macromolecules at the blood–brain barrier. Pharmacology of the blood brain barrier: targeting CNS disorders.

[64] Lajoie JM, Shusta EV (2015). Targeting receptor-mediated transport for delivery of biologics across the blood–brain barrier. Annu Rev Pharmacol Toxicol.

[65] Rosenberg GA, Kyner WT, Estrada E (1980). Bulk flow of brain interstitial fluid under normal and hyperosmolar conditions. Am J Physiol.

[66] Terasaki T, Pardridge WM (1998). Development of brain efflux index (BEI) method and its application to the blood–brain barrier efflux transport study. Introduction to the blood–brain barrier methodology, biology and pathology.

[67] Liu S, Lam MA, Sial A, Hemley SJ, Bilston LE, Stoodley MA (2018). Fluid outflow in the rat spinal cord: the role of perivascular and paravascular pathways. Fluids Barriers CNS.

[68] Wagner HJ, Pilgrim C, Brandl J (1974). Penetration and removal of horseradish peroxidase injected into the cerebrospinal fluid: role of cerebral perivascular spaces, endothelium and microglia. Acta Neuropathol.

[69] Rennels ML, Blaumanis OR, Grady PA (1990). Rapid solute transport throughout the brain via paravascular fluid pathways. Adv Neurol.

[70] Carare RO, Bernardes-Silva M, Newman TA, Page AM, Nicoll JAR, Perry VH, Weller RO (2008). Solutes, but not cells, drain from the brain parenchyma along basement membranes of capillaries and arteries: significance for cerebral amyloid angiopathy and neuroimmunology. Neuropathol Appl Neurobiol.

[71] Bedussi B, van der Wel NN, de Vos J, van Veen H, Siebes M, VanBavel E, Bakker ENTP (2017). Paravascular channels, cisterns, and the subarachnoid space in the rat brain: a single compartment with preferential pathways. J Cereb Blood Flow Metab.

[72] Hannocks M-J, Pizzo ME, Huppert J, Deshpande T, Abbott NJ, Thorne RG, Sorokin L (2017). Molecular characterization of perivascular drainage pathways in the murine brain. J Cereb Blood Flow Metab.

[73] Asgari M, de Zelicourt D, Kurtcuoglu V (2015). How astrocyte networks may contribute to cerebral metabolite clearance. Sci Rep.

[74] Nicholson C, Sykova E (1998). Extracellular space structure revealed by diffusion analysis. Trends Neurosci.

[75] Nicholson C (2001). Diffusion and related transport mechanisms in brain tissue. Rep Prog Phys.

[76] Wolak DJ, Thorne RG (2013). Diffusion of macromolecules in the brain: implications for drug delivery. Mol Pharm.

[77] Jin B-J, Smith AJ, Verkman AS (2016). Spatial model of convective solute transport in brain extracellular space does not support a “glymphatic” mechanism. J Gen Physiol.

[78] Asgari M, de Zélicourt DA, Kurtcuoglu V (2016). Glymphatic solute transport does not require bulk flow. Sci Rep.

[79] Smith AJ, Yao X, Dix JA, Jin B-J, Verkman AS (2017). Test of the ‘glymphatic’ hypothesis demonstrates diffusive and aquaporin-4-independent solute transport in rodent brain parenchyma. eLife.

[80] Holter KE, Kehlet B, Devor A, Sejnowski TJ, Dale AM, Omholt SW, Ottersen OP, Nagelhus EA, Mardal K-A, Pettersen KH (2017). Interstitial solute transport in 3D reconstructed neuropil occurs by diffusion rather than bulk flow. Proc Natl Acad Sci USA.

[81] Pizzo ME, Thorne RG, Jerome B, Plesnila N (2017). Chapter 6—the extracellular and perivascular spaces of the brain. Brain edema.

[82] Bradbury MW, Cserr HF, Westrop RJ (1981). Drainage of cerebral interstitial fluid into deep cervical lymph of the rabbit. Am J Physiol.

[83] Szentistvanyi I, Patlak CS, Ellis RA, Cserr HF (1984). Drainage of interstitial fluid from different regions of rat brain. Am J Physiol.

[84] Ichimura T, Fraser PA, Cserr HF (1991). Distribution of extracellular tracers in perivascular spaces of the rat brain. Brain Res.

[85] Ball KK, Cruz NF, Mrak RE, Dienel GA (2010). Trafficking of glucose, lactate, and amyloid-beta from the inferior colliculus through perivascular routes. J Cereb Blood Flow Metab.

[86] Barua NU, Bienemann AS, Hesketh S, Wyatt MJ, Castrique E, Love S, Gill SS (2012). Intrastriatal convection-enhanced delivery results in widespread perivascular distribution in a pre-clinical model. Fluids Barriers CNS.

[87] Arbel-Ornath M, Hudry E, Eikermann-Haerter K, Hou S, Gregory JL, Zhao LZ, Betensky RA, Frosch MP, Greenberg SM, Bacskai BJ (2013). Interstitial fluid drainage is impaired in ischemic stroke and Alzheimer’s disease mouse models. Acta Neuropathol.

[88] Albargothy NJ, Johnston DA, MacGregor-Sharp M, Weller RO, Verma A, Hawkes CA, Carare RO (2018). Convective influx/glymphatic system: tracers injected into the CSF enter and leave the brain along separate periarterial basement membrane pathways. Acta Neuropathol.

[89] Rangroo Thrane V, Thrane AS, Plog BA, Thiyagarajan M, Iliff JJ, Deane R, Nagelhus EA, Nedergaard M (2013). Paravascular microcirculation facilitates rapid lipid transport and astrocyte signaling in the brain. Sci Rep.

[90] Lochhead JJ, Wolak DJ, Pizzo ME, Thorne RG (2015). Rapid transport within cerebral perivascular spaces underlies widespread tracer distribution in the brain after intranasal administration. J Cereb Blood Flow Metab.

[91] Ratner V, Gao Y, Lee H, Elkin R, Nedergaard M, Benveniste H, Tannenbaum A (2017). Cerebrospinal and interstitial fluid transport via the glymphatic pathway modeled by optimal mass transport. Neuroimage.

[92] Goulay R, Flament J, Gauberti M, Naveau M, Pasquet N, Gakuba C, Emery E, Hantraye P, Vivien D, Aron-Badin R, Gaberel T (2017). Subarachnoid hemorrhage severely impairs brain parenchymal cerebrospinal fluid circulation in nonhuman primate. Stroke.

[93] Lee JC, Olszewski J (1960). Penetration of radioactive bovine albumin from cerebrospinal fluid into brain tissue. Neurology.

[94] Aspelund A, Antila S, Proulx ST, Karlsen TV, Karaman S, Detmar M, Wiig H, Alitalo K (2015). A dural lymphatic vascular system that drains brain interstitial fluid and macromolecules. J Exp Med.

[95] Morris AWJ, Sharp MM, Albargothy NJ, Fernandes R, Hawkes CA, Verma A, Weller RO, Carare RO (2016). Vascular basement membranes as pathways for the passage of fluid into and out of the brain. Acta Neuropathol.

[96] Bakker ENTP, Bacskai BJ, Arbel-Ornath M, Aldea R, Bedussi B, Morris AWJ, Weller RO, Carare RO (2016). Lymphatic clearance of the brain: perivascular, paravascular and significance for neurodegenerative diseases. Cell Mol Neurobiol.

[97] Carare RO, Teeling JL, Hawkes CA, Puntener U, Weller RO, Nicoll JAR, Perry VH (2013). Immune complex formation impairs the elimination of solutes from the brain: implications for immunotherapy in Alzheimer’s disease. Acta Neuropathol Commun.

[98] Weller RO, Sharp MM, Christodoulides M, Carare RO, Mollgard K (2018). The meninges as barriers and facilitators for the movement of fluid, cells and pathogens related to the rodent and human CNS. Acta Neuropathol.

[99] Sharp MM, Bulters D, Brandner S, Holton J, Verma A, Werring DJ, Carare RO (2018). The fine anatomy of the perivascular compartment in the human brain: relevance to dilated perivascular spaces in cerebral amyloid angiopathy. Neuropathol Appl Neurobiol.

[100] Diem AK, Sharp MM, Gatherer M, Bressloff NW, Carare RO, Richardson G (2017). Arterial pulsations cannot drive intramural periarterial drainage: significance for Abeta drainage. Front Neurosci.

[101] Aldea R. Modelling cerebral interstitial flows and their failure in Alzheimer’s disease. Ph.D. University of Southampton, Faculty of Social, Human and Mathematical Sciences School of Mathematical Sciences, Applied Mathematics; 2017.

[102] Carare RO, Hawkes CA, Jeffrey M, Kalaria RN, Weller RO (2013). Review: cerebral amyloid angiopathy, prion angiopathy, CADASIL and the spectrum of protein elimination failure angiopathies (PEFA) in neurodegenerative disease with a focus on therapy. Neuropathol Appl Neurobiol.

[103] Zhang ET, Inman CBE, Weller RO (1990). Interrelationships of the pia mater and the perivascular (Virchow-Robin) spaces in the human cerebrum. J Anat.

[104] Zhang ET, Richards HK, Kida S, Weller RO (1992). Directional and compartmentalized drainage of interstitial fluid and cerebrospinal-fluid from the rat-brain. Acta Neuropathol.

[105] Weller RO, Djuanda E, Yow H-Y, Carare RO (2009). Lymphatic drainage of the brain and the pathophysiology of neurological disease. Acta Neuropathol.

[106] Carare RO, Hawkes CA, Weller RO (2014). Afferent and efferent immunological pathways of the brain. Anatomy, function and failure. Brain Behav Immun.

[107] Engelhardt B, Carare RO, Bechmann I, Flugel A, Laman JD, Weller RO (2016). Vascular, glial, and lymphatic immune gateways of the central nervous system. Acta Neuropathol.

[108] Bedussi B, Almasian M, de Vos J, VanBavel E, Bakker ENTP (2017). Paravascular spaces at the brain surface: low resistance pathways for cerebrospinal fluid flow. J Cereb Blood Flow Metab.

[109] Louveau A, Plog BA, Antila S, Alitalo K, Nedergaard M, Kipnis J (2017). Understanding the functions and relationships of the glymphatic system and meningeal lymphatics. J Clin Invest.

[110] Louveau A, Smirnov I, Keyes TJ, Eccles JD, Rouhani SJ, Peske JD, Derecki NC, Castle D, Mandell JW, Lee KS (2015). Structural and functional features of central nervous system lymphatic vessels. Nature.

[111] Ma Q, Ineichen BV, Detmar M, Proulx ST (2017). Outflow of cerebrospinal fluid is predominantly through lymphatic vessels and is reduced in aged mice. Nat Commun.

[112] Antila S, Karaman S, Nurmi H, Airavaara M, Voutilainen MH, Mathivet T, Chilov D, Li Z, Koppinen T, Park J-H (2017). Development and plasticity of meningeal lymphatic vessels. J Exp Med.

[113] Absinta M, Ha S-K, Nair G, Sati P, Luciano NJ, Palisoc M, Louveau A, Zaghloul KA, Pittaluga S, Kipnis J, Reich DS (2017). Human and nonhuman primate meninges harbor lymphatic vessels that can be visualized noninvasively by MRI. eLife.

[114] Kwon S, Janssen CF, Velasquez F, Sevick-Muraca E. Fluorescence imaging of lymphatic outflow of cerebrospinal fluid in mice. In: Procedings of SPIE 10578, medical imaging 2018: biomedical applications in molecular, structural, and functional imaging; 12 March 2018; Houston, TX USA. 2018. p. 1057816.

[115] Eide PK, Vatnehol SAS, Emblem KE, Ringstad G (2018). Magnetic resonance imaging provides evidence of glymphatic drainage from human brain to cervical lymph nodes. Sci Rep.

[116] Bradbury MW, Cole DF (1980). The role of the lymphatic system in drainage of cerebrospinal fluid and aqueous humour. J Physiol (Lond).

[117] Bradbury MWB, Cserr HF, Westrop RJ (1980). Drainage of cerebral interstitial fluid into deep cervical lymph of the rabbit. J Physiol (Lond).

[118] Kida S, Pantazis A, Weller RO (1993). CSF drains directly from the subarachnoid space into nasal lymphatics in the rat—anatomy, histology and immunological significance. Neuropathol Appl Neurobiol.

[119] Pollay M (2010). The function and structure of the cerebrospinal fluid outflow system. Cerebrospinal Fluid Res.

[120] Johnston M, Zakharov A, Koh L, Armstrong D (2005). Subarachnoid injection of Microfil reveals connections between cerebrospinal fluid and nasal lymphatics in the non-human primate. Neuropathol Appl Neurobiol.

[121] Nagra G, Koh L, Zakharov A, Armstrong D, Johnston M (2006). Quantification of cerebrospinal fluid transport across the cribriform plate into lymphatics in rats. Am J Physiol Regul Integr Comp Physiol.

[122] Koh L, Zakharov A, Johnston M (2005). Integration of the subarachnoid space and lymphatics: is it time to embrace a new concept of cerebrospinal fluid absorption?. Cerebrospinal Fluid Res.

[123] Dienel GA, Cruz NF (2008). Imaging brain activation: simple pictures of complex biology. Ann NY Acad Sci.

[124] Dienel GA, Cruz NF (2016). Aerobic glycolysis during brain activation: adrenergic regulation and influence of norepinephrine on astrocytic metabolism. J Neurochem.

[125] Bradbury MWB, Westrop RJ (1983). Factors influencing exit of substances from cerebrospinal-fluid into deep cervical lymph of the rabbit. J Physiol (Lond).

[126] Cserr HF, Cooper DN, Milhorat TH (1977). Flow of cerebral interstitial fluid as indicated by removal of extracellular markers from rat caudate-nucleus. Exp Eye Res.

[127] Cserr HF, Cooper DN, Suri PK, Patlak CS (1981). Efflux of radiolabeled polyethylene glycols and albumin from rat brain. Am J Physiol.

[128] Xie L, Kang H, Xu Q, Chen MJ, Liao Y, Thiyagarajan M, O’Donnell J, Christensen DJ, Nicholson C, Iliff JJ (2013). Sleep drives metabolite clearance from the adult brain. Science.

[129] Cserr HF, Ostrach LH (1974). Bulk flow of interstitial fluid after intracranial injection of blue dextran 2000. Exp Neurol.

[130] Cserr HF, Patlak CS, Bradbury MWB (1992). Secretion and bulk flow of interstitial fluid. Physiology and pharmacology of the blood–brain barrier.

[131] Groothuis DR, Vavra MW, Schlageter KE, Kang EW-Y, Itskovich AC, Hertzler S, Allen CV, Lipton HL (2007). Efflux of drugs and solutes from brain: the interactive roles of diffusional transcapillary transport, bulk flow and capillary transporters. J Cereb Blood Flow Metab.

[132] Rey J, Sarntinoranont M (2018). Pulsatile flow drivers in brain parenchyma and perivascular spaces: a resistance network model study. Fluids Barriers CNS.

[133] Papisov MI, Belov VV, Gannon KS (2013). Physiology of the intrathecal bolus: the leptomeningeal route for macromolecule and particle delivery to CNS. Mol Pharm.

[134] Asgari M, de Zelicourt DA, Kurtcuoglu V (2017). Barrier dysfunction or drainage reduction: differentiating causes of CSF protein increase. Fluids Barriers CNS.

[135] Rennels ML, Gregory TF, Blaumanis OR, Fujimoto K, Grady PA (1985). Evidence for a paravascular fluid circulation in the mammalian central nervous system, provided by the rapid distribution of tracer protein throughout the brain from the subarachnoid space. Brain Res.

[136] Nedergaard M (2013). Neuroscience. Garbage truck of the brain. Science.

[137] Plog BA, Nedergaard M (2018). The glymphatic system in central nervous system health and disease: past, present, and future. Annu Rev Pathol.

[138] Levick JR (1987). Flow through interstitium and other fibrous matrices. Quart J Exp Physiol.

[139] Lei Y, Han H, Yuan F, Javeed A, Zhao Y (2017). The brain interstitial system: anatomy, modeling, in vivo measurement, and applications. Prog Neurobiol.

[140] Smith AJ, Verkman AS (2018). The “glymphatic” mechanism for solute clearance in Alzheimer’s disease: game changer or unproven speculation?. FASEB J.

[141] Kinney JP, Spacek J, Bartol TM, Bajaj CL, Harris KM, Sejnowski TJ (2013). Extracellular sheets and tunnels modulate glutamate diffusion in hippocampal neuropil. J Comp Neurol.

[142] Thorne RG, Nicholson C (2006). In vivo diffusion analysis with quantum dots and dextrans predicts the width of brain extracellular space. Proc Natl Acad Sci USA.

[143] Nicholson C, Hrabetova S (2017). Brain extracellular space: the final frontier of neuroscience. Biophys J.

[144] Mestre H, Kress BT, Zou W, Pu T, Murlidharan G, Rivera RMC, Simon MJ, Pike MM, Plog BA, Xavier ALR (2017). Aquaporin-4 dependent glymphatic solute transport in rodent brain. bioRxiv.

[145] Iliff JJ, Thrane AS, Nedergaard M, Caplan LR, Biller J, Leary MC, Lo EH, Thomas AJ, Yenari M, Zhang JH (2017). Cerebrovascular anatomy and hemodynamics. Primer on cerebrovascular diseases.

[146] Hladky SB, Barrand MA (2017). Metabolite clearance during wakefulness and sleep. Handb Exp Pharmacol.

[147] Gakuba C, Gaberel T, Goursaud S, Bourges J, Di Palma C, Quenault A, de Lizarrondo SM, Vivien D, Gauberti M (2018). General anesthesia inhibits the activity of the “glymphatic system”. Theranostics.

[148] Purves MJ (1972). The physiology of the cerebral circulation.

[149] Mathiisen TM, Lehre KP, Danbolt NC, Ottersen OP (2010). The perivascular astroglial sheath provides a complete covering of the brain microvessels: an electron microscopic 3D reconstruction. Glia.

[150] Korogod N, Petersen CCH, Knott GW (2015). Ultrastructural analysis of adult mouse neocortex comparing aldehyde perfusion with cryo fixation. eLife.

[151] Crone C (1986). The blood–brain barrier as a tight epithelium: where is information lacking?. Ann NY Acad Sci.

[152] Davson H, Segal MB (1970). The effects of some inhibitors and accelerators of sodium transport on the turnover of ^22^Na in the cerebrospinal fluid and the brain. J Physiol (Lond).

[153] Ennis SR, Ren X-D, Betz AL (1996). Mechanisms of sodium transport at the blood–brain barrier studied with in situ perfusion of rat brain. J Neurochem.

[154] Brasnjevic I, Steinbusch HWM, Schmitz C, Martinez-Martinez P (2009). Delivery of peptide and protein drugs over the blood–brain barrier. Prog Neurobiol.

[155] Thovert G (1910). Diffusion and kinetic theory of solutions. C R Hebd Seances Acad Sci.

[156] Davson H, Danielli JF (1943). The permeability of natural membranes.

[157] Stein WD (1967). The movement of molecules across cell membranes.

[158] Davson H, Danielli JF (1952). The pemeability of natural membranes.

[159] Fenstermacher JD, Rapoport SI, Renkin EM, Michel CC (1984). Blood–brain barrier. The cardiovascular system vol 4, microcirculation. Volume 4 Part 2.

[160] Habgood MD, Begley DJ, Abbott NJ (2000). Determinants of passive drug entry into the central nervous system. Cell Mol Neurobiol.

[161] Fong CW (2015). Permeability of the blood–brain barrier: molecular mechanism of transport of drugs and physiologically important compounds. J Membr Biol.

[162] Levin VA (1980). Relationship of octanol/water partition coefficient and molecular weight to rat brain capillary permeability. J Med Chem.

[163] Bodor N, Buchwald P (2003). Brain-targeted drug delivery. Am J Drug Deliv.

[164] Lipinski CA, Lombardo F, Dominy BW, Feeney PJ (2001). Experimental and computational approaches to estimate solubility and permeability in drug discovery and development settings. Adv Drug Deliv Rev.

[165] Abraham MH, Chadha HS, Pliska V, Testa B, van de Waterbeemd H (1996). Applications of a solvation equation to drug transport properties. Lipophilicity in drug action and toxicity.

[166] Gratton JA, Abraham MH, Bradbury MW, Chadha HS (1997). Molecular factors influencing drug transfer across the blood–brain barrier. J Pharm Pharmacol.

[167] Abraham MH (2004). The factors that influence permeation across the blood–brain barrier. Eur J Med Chem.

[168] Abraham MH (2011). The permeation of neutral molecules, ions, and ionic species through membranes: brain permeation as an example. J Pharm Sci.

[169] Liu X, Tu M, Kelly RS, Chen C, Smith BJ (2004). Development of a computational approach to predict blood–brain barrier permeability. Drug Metab Dispos.

[170] van de Waterbeemd H, Camenisch G, Folkers G, Chretien JR, Raevsky OA (1998). Estimation of blood–brain barrier crossing of drugs using molecular size and shape, and H-bonding descriptors. J Drug Target.

[171] Abraham MH, Acree WE (2016). Descriptors for ions and ion-pairs for use in linear free energy relationships. J Chromatogr A.

[172] Geldenhuys WJ, Mohammad AS, Adkins CE, Lockman PR (2015). Molecular determinants of blood–brain barrier permeation. Ther Deliv.

[173] Enerson BE, Drewes LR (2006). The rat blood–brain barrier transcriptome. J Cereb Blood Flow Metab.

[174] Warren MS, Zerangue N, Woodford K, Roberts LM, Tate EH, Feng B, Li C, Feuerstein TJ, Gibbs J, Smith B (2009). Comparative gene expression profiles of ABC transporters in brain microvessel endothelial cells and brain in five species including human. Pharmacol Res.

[175] Daneman R, Zhou L, Agalliu D, Cahoy JD, Kaushal A, Barres BA (2010). The mouse blood–brain barrier transcriptome: a new resource for understanding the development and function of brain endothelial cells. PLoS ONE.

[176] Shawahna R, Uchida Y, Decleves X, Ohtsuki S, Yousif S, Dauchy S, Jacob A, Chassoux F, Daumas-Duport C, Couraud P-O (2011). Transcriptomic and quantitative proteomic analysis of transporters and drug metabolizing enzymes in freshly isolated human brain microvessels. Mol Pharm.

[177] Zhang Y, Chen K, Sloan SA, Bennett ML, Scholze AR, O’Keeffe S, Phatnani HP, Guarnieri P, Caneda C, Ruderisch N (2014). An RNA-sequencing transcriptome and splicing database of glia, neurons, and vascular cells of the cerebral cortex. J Neurosci.

[178] Suhy AM, Webb A, Papp AC, Geier EG, Sadee W (2017). Expression and splicing of ABC and SLC transporters in the human blood–brain barrier measured with RNAseq. Eur J Pharm Sci.

[179] Ohtsuki S, Terasaki T (2007). Contribution of carrier-mediated transport systems to the blood–brain barrier as a supporting and protecting interface for the brain; importance for CNS drug discovery and development. Pharm Res.

[180] Roberts LM, Black DS, Raman C, Woodford K, Zhou M, Haggerty JE, Yan AT, Cwirla SE, Grindstaff KK (2008). Subcellular localization of transporters along the rat blood–brain barrier and blood-cerebral-spinal fluid barrier by in vivo biotinylation. Neuroscience.

[181] Dauchy S, Dutheil F, Weaver RJ, Chassoux F, Daumas-Duport C, Couraud P-O, Scherrmann J-M, De Waziers I, Decleves X (2008). ABC transporters, cytochromes P450 and their main transcription factors: expression at the human blood–brain barrier. J Neurochem.

[182] Kamiie J, Ohtsuki S, Iwase R, Ohmine K, Katsukura Y, Yanai K, Sekine Y, Uchida Y, Ito S, Terasaki T (2008). Quantitative atlas of membrane transporter proteins: development and application of a highly sensitive simultaneous LC/MS/MS method combined with novel in silico peptide selection criteria. Pharm Res.

[183] Chun HB, Scott M, Niessen S, Hoover H, Baird A, Yates J, Torbett BE, Eliceiri BP (2011). The proteome of mouse brain microvessel membranes and basal lamina. J Cereb Blood Flow Metab.

[184] Uchida Y, Ohtsuki S, Katsukura Y, Ikeda C, Suzuki T, Kamiie J, Terasaki T (2011). Quantitative targeted absolute proteomics of human blood–brain barrier transporters and receptors. J Neurochem.

[185] Hoshi Y, Uchida Y, Tachikawa M, Inoue T, Ohtsuki S, Terasaki T (2013). Quantitative atlas of blood–brain barrier transporters, receptors, and tight junction proteins in rats and common marmoset. J Pharm Sci.

[186] Ohtsuki S, Hirayama M, Ito S, Uchida Y, Tachikawa M, Terasaki T (2014). Quantitative targeted proteomics for understanding the blood–brain barrier: towards pharmacoproteomics. Expert Rev Proteomics.

[187] Worzfeld T, Schwaninger M (2016). Apicobasal polarity of brain endothelial cells. J Cereb Blood Flow Metab.

[188] Zhang Z, Uchida Y, Hirano S, Ando D, Kubo Y, Auriola S, Akanuma S-I, Hosoya K-I, Urtti A, Terasaki T, Tachikawa M (2017). Inner blood-retinal barrier dominantly expresses breast cancer resistance protein: comparative quantitative targeted absolute proteomics study of CNS barriers in pig. Mol Pharm.

[189] Pardridge WM (1983). Brain metabolism: a perspective from the blood–brain barrier. Physiol Rev.

[190] Pardridge WM (1998). Introduction to the blood–brain barrier.

[191] Lee G, Dallas S, Hong M, Bendayan R (2001). Drug transporters in the central nervous system: brain barriers and brain parenchyma considerations. Pharmacol Rev.

[192] Redzic ZB, Biringer J, Barnes K, Baldwin SA, Al-Sarraf H, Nicola PA, Young JD, Cass CE, Barrand MA, Hladky SB (2005). Polarized distribution of nucleoside transporters in rat brain endothelial and choroid plexus epithelial cells. J Neurochem.

[193] Terasaki T, Ohtsuki S (2005). Brain-to-blood transporters for endogenous substrates and xenobiotics at the blood–brain barrier: an overview of biology and methodology. NeuroRx.

[194] Eyal S, Hsiao P, Unadkat JD (2009). Drug interactions at the blood–brain barrier: fact or fantasy?. Pharmacol Ther.

[195] O’Donnell ME, Alvarez-Leefmans FJ, Delpire E (2009). Ion and water transport across the blood–brain barrier. Physiology and pathology of chloride transporters and channels in the nervous system: from molecules to diseases.

[196] Hartz AM, Bauer B (2011). ABC transporters in the CNS—an inventory. Curr Pharm Biotechnol.

[197] Chaves C, Shawahna R, Jacob A, Scherrmann J-M, Decleves X (2014). Human ABC transporters at blood-CNS interfaces as determinants of CNS drug penetration. Curr Pharm Des.

[198] Daneman R, Prat A (2015). The blood–brain barrier. Cold Spring Harb Perspect Biol.

[199] Qosa H, Miller DS, Pasinelli P, Trotti D (2015). Regulation of ABC efflux transporters at blood–brain barrier in health and neurological disorders. Brain Res.

[200] Nalecz KA (2017). Solute carriers in the blood–brain barier: safety in abundance. Neurochem Res.

[201] Hediger MA, Clemencon B, Burrier RE, Bruford EA (2013). The ABCs of membrane transporters in health and disease (SLC series): introduction. Molec Aspects Med.

[202] Hediger MA. BioParadigms SLC tables. http://www.bioparadigms.org/slc/intro.htm. Accessed 27 Aug 2018.

[203] Schinkel AH, Smit JJ, van Tellingen O, Beijnen JH, Wagenaar E, van Deemter L, Mol CA, van der Valk MA, Robanus-Maandag EC, te Riele HP (1994). Disruption of the mouse mdr1a *P*-glycoprotein gene leads to a deficiency in the blood–brain barrier and to increased sensitivity to drugs. Cell.

[204] Cordon-Cardo C, O’Brien JP, Casals D, Rittman-Grauer L, Biedler JL, Melamed MR, Bertino JR (1989). Multidrug-resistance gene (*P*-glycoprotein) is expressed by endothelial cells at blood–brain barrier sites. Proc Natl Acad Sci USA.

[205] Thiebaut F, Tsuruo T, Hamada H, Gottesman MM, Pastan I, Willingham MC (1989). Immunohistochemical localization in normal tissues of different epitopes in the multidrug transport protein P170: evidence for localization in brain capillaries and crossreactivity of one antibody with a muscle protein. J Histochem Cytochem.

[206] Sugawara I, Hamada H, Tsuruo T, Mori S (1990). Specialized localization of *P*-glycoprotein recognized by MRK 16 monoclonal antibody in endothelial cells of the brain and the spinal cord. Jpn J Cancer Res.

[207] Tatsuta T, Naito M, Ohhara T, Sugawara I, Tsuruo T (1992). Functional involvement of *P*-glycoprotein in blood–brain barrier. J Biol Chem.

[208] Virgintino D, Robertson D, Errede M, Benagiano V, Girolamo F, Maiorano E, Roncali L, Bertossi M (2002). Expression of *P*-glycoprotein in human cerebral cortex microvessels. J Histochem Cytochem.

[209] Soontornmalai A, Vlaming ML, Fritschy JM (2006). Differential, strain-specific cellular and subcellular distribution of multidrug transporters in murine choroid plexus and blood–brain barrier. Neuroscience.

[210] Raviv Y, Pollard HB, Bruggemann EP, Pastan I, Gottesman MM (1990). Photosensitized labeling of a functional multidrug transporter in living drug-resistant tumor cells. J Biol Chem.

[211] Altenberg G, Vanoye CG, Horton JK, Reuss L (1994). Unidirectional fluxes of rhodamine 123 in multidrug-resistant cells: evidence against direct drug extrusion from the plasma membrane. Proc Natl Acad Sci USA.

[212] Sharom FJ (2014). Complex interplay between the *P*-glycoprotein multidrug efflux pump and the membrane: its role in modulating protein function. Front Oncol.

[213] Aller SG, Yu J, Ward A, Weng Y, Chittaboina S, Zhuo R, Harrell PM, Trinh YT, Zhang Q, Urbatsch IL, Chang G (2009). Structure of *P*-glycoprotein reveals a molecular basis for poly-specific drug binding. Science.

[214] Cooray HC, Blackmore CG, Maskell L, Barrand MA (2002). Localisation of breast cancer resistance protein in microvessel endothelium of human brain. NeuroReport.

[215] Eisenblatter T, Huwel S, Galla HJ (2003). Characterisation of the brain multidrug resistance protein (BMDP/ABCG2/BCRP) expressed at the blood–brain barrier. Brain Res.

[216] Zhang WD, Mojsilovic-Petrovic J, Andrade MF, Zhang H, Ball M, Stanimirovic DB (2003). The expression and functional characterization of ABCG2 in brain endothelial cells and vessels. FASEB J.

[217] Cisternino S, Mercier C, Bourasset F, Roux F, Scherrmann JM (2004). Expression, up-regulation, and transport activity of the multidrug-resistance protein ABCG2 at the mouse blood–brain barrier. Cancer Res.

[218] Yousif S, Marie-Claire C, Roux F, Scherrmann JM, Decleves X (2007). Expression of drug transporters at the blood–brain barrier using an optimized isolated rat brain microvessel strategy. Brain Res.

[219] Leggas M, Adachi M, Scheffer GL, Sun D, Wielinga P, Du G, Mercer KE, Zhuang Y, Panetta JC, Johnston B (2004). Mrp4 confers resistance to topotecan and protects the brain from chemotherapy. Mol Cell Biol.

[220] Nies AT, Jedlitschky G, Konig J, Herold-Mende C, Steiner HH, Schmitt HP, Keppler D (2004). Expression and immunolocalization of the multidrug resistance proteins, Mrp1-Mrp6 (ABCC1-ABCC6), in human brain. Neuroscience.

[221] Miller DS, Davis TP (2014). ABC transporter regulation by signaling at the blood–brain barrier: relevance to pharmacology. Pharmacology of the blood brain barrier targeting CNS disorders.

[222] Seetharaman S, Barrand MA, Maskell L, Scheper RJ (1998). Multidrug resistance-related transport proteins in isolated human brain microvessels and in cells cultured from these isolates. J Neurochem.

[223] Regina A, Koman A, Piciotti M, El Hafny B, Center MS, Bergmann R, Couraud PO, Roux F (1998). Mrp1 multidrug resistance-associated protein and *P*-glycoprotein expression in rat brain microvessel endothelial cells. J Neurochem.

[224] Gutmann H, Torok M, Fricker G, Huwyler J, Beglinger C, Drewe J (1999). Modulation of multidrug resistance protein expression in porcine brain capillary endothelial cells in vitro. Drug Metab Dispos.

[225] Cisternino S, Rousselle C, Lorico A, Rappa G, Scherrmann JM (2003). Apparent lack of Mrp1-mediated efflux at the luminal side of mouse blood–brain barrier endothelial cells. Pharm Res.

[226] Gazzin S, Strazielle N, Schmitt C, Fevre-Montange M, Ostrow JD, Tiribelli C, Ghersi-Egea J-F (2008). Differential expression of the multidrug resistance-related proteins ABCb1 and ABCc1 between blood–brain interfaces. J Comp Neurol.

[227] Dombrowski SM, Desai SY, Marroni M, Cucullo L, Goodrich K, Bingaman W, Mayberg MR, Bengez L, Janigro D (2001). Overexpression of multiple drug resistance genes in endothelial cells from patients with refractory epilepsy. Epilepsia.

[228] Aronica E, Gorter JA, Ramkema M, Redeker S, Ozbas-Gerceker F, van Vliet EA, Scheffer GL, Scheper RJ, van der Valk P, Baayen JC, Troost D (2004). Expression and cellular distribution of multidrug resistance-related proteins in the hippocampus of patients with mesial temporal lobe epilepsy. Epilepsia.

[229] van Vliet EA, Redeker S, Aronica E, Edelbroek PM, Gorter JA (2005). Expression of multidrug transporters MRP1, MRP2, and BCRP shortly after status epilepticus, during the latent period, and in chronic epileptic rats. Epilepsia.

[230] Sun H, Dai H, Shaik N, Elmquist WF (2003). Drug efflux transporters in the CNS. Adv Drug Deliv Rev.

[231] Begley DJ (2004). ABC transporters and the blood–brain barrier. Curr Pharm Des.

[232] Loscher W, Potschka H (2005). Drug resistance in brain diseases and the role of drug efflux transporters. Nat Rev Neurosci.

[233] Breedveld P, Beijnen JH, Schellens JHM (2006). Use of *P*-glycoprotein and BCRP inhibitors to improve oral bioavailability and CNS penetration of anticancer drugs. Trends Pharmacol Sci.

[234] Dallas S, Miller DS, Bendayan R (2006). Multidrug resistance-associated proteins: expression and function in the central nervous system. Pharmacol Rev.

[235] Koepsell H, Endou H (2004). The SLC22 drug transporter family. Pflügers Arch.

[236] Kusuhara H, Sugiyama Y (2005). Active efflux across the blood–brain barrier: role of the solute carrier family. NeuroRx.

[237] Bronger H, Konig J, Kopplow K, Steiner HH, Ahmadi R, Herold-Mende C, Keppler D, Nies AT (2005). ABCC drug efflux pumps and organic anion uptake transporters in human gliomas and the blood-tumor barrier. Cancer Res.

[238] Roth M, Obaidat A, Hagenbuch B (2012). OATPs, OATs and OCTs: the organic anion and cation transporters of the SLCO and SLC22A gene superfamilies. Br J Pharmacol.

[239] Obaidat A, Roth M, Hagenbuch B (2012). The expression and function of organic anion transporting polypeptides in normal tissues and in cancer. Annu Rev Pharmacol Toxicol.

[240] Geier EG, Chen EC, Webb A, Papp AC, Yee SW, Sadee W, Giacomini KM (2013). Profiling solute carrier transporters in the human blood–brain barrier. Clin Pharmacol Ther.

[241] Ronaldson PT, Davis TP (2013). Targeted drug delivery to treat pain and cerebral hypoxia. Pharmacol Rev.

[242] Hagenbuch B, Stieger B (2013). The SLCO (former SLC21) superfamily of transporters. Molec Aspects Med.

[243] Ashraff T, Ronaldson PT, Bendayan R, You G (2014). Drug transport in the brain. Drug transporters: molecular characterization and role in drug disposition.

[244] Farthing CA, Sweet DH (2014). Expression and function of organic cation and anion transporters (SLC22 family) in the CNS. Curr Pharm Des.

[245] Zhou FF, Zhu L, Wang K, Murray M (2017). Recent advance in the pharmacogenomics of human solute carrier transporters (SLCs) in drug disposition. Adv Drug Deliv Rev.

[246] Kovacsics D, Patik I, Ozvegy-Laczka C (2017). The role of organic anion transporting polypeptides in drug absorption, distribution, excretion and drug-drug interactions. Expert Opin Drug Metab Toxicol.

[247] Hagenbuch B, Meier PJ (2004). Organic anion transporting polypeptides of the OATP/SLC21 family: phylogenetic classification as OATP/SLCO superfamily, new nomenclature and molecular/functional properties. Pflügers Arch.

[248] Reese TS, Karnovsky MJ (1967). Fine structural localization of a blood–brain barrier to exogenous peroxidase. J Cell Biol.

[249] Stewart PA (2000). Endothelial vesicles in the blood–brain barrier: are they related to permeability?. Cell Mol Neurobiol.

[250] Tuma PL, Hubbard AL (2003). Transcytosis: crossing cellular barriers. Physiol Rev.

[251] Herve F, Ghinea N, Scherrmann J-M (2008). CNS delivery via adsorptive transcytosis. AAPS J.

[252] Pan W, Kastin AJ (2006). Permeability of the blood–brain barrier to neurotrophic peptides. Handbook of biologically active peptides.

[253] Pardridge WM, Triguero D, Buciak J (1989). Transport of histone through the blood–brain barrier. J Pharmacol Exp Ther.

[254] Zorko M, Langel U (2005). Cell-penetrating peptides: mechanism and kinetics of cargo delivery. Adv Drug Deliv Rev.

[255] Banks WA, Akerstrom V, Kastin AJ (1998). Adsorptive endocytosis mediates the passage of HIV-1 across the blood–brain barrier: evidence for a post-internalization coreceptor. J Cell Sci.

[256] Batrakova EV, Kabanov AV, Touitou E, Barry BW (2007). Strategies to overcome the blood–brain barrier. Enhancement in drug delivery.

[257] Villegas JC, Broadwell RD (1993). Transcytosis of protein through the mammalian cerebral epithelium and endothelium. II. Adsorptive transcytosis of WGA-HRP and the blood–brain and brain–blood barriers. J Neurocytol.

[258] Pardridge WM, Buciak JL, Kang YS, Boado RJ (1993). Protamine-mediated transport of albumin into brain and other organs of the rat—binding and endocytosis of protamine-albumin complex by microvascular endothelium. J Clin Invest.

[259] Banks WA, Broadwell RD (1994). Blood to brain and brain to blood passage of native horseradish peroxidase, wheat germ agglutinin, and albumin: pharmacokinetic and morphological assessments. J Neurochem.

[260] Broadwell RD (1989). Transcytosis of macromolecules through the blood–brain barrier: a cell biological perspective and critical appraisal. Acta Neuropathol.

[261] Visser CC, Voorwinden LH, Crommelin DJA, Danhof M, de Boer AG (2004). Characterization and modulation of the transferrin receptor on brain capillary endothelial cells. Pharm Res.

[262] Smith MW, Gumbleton M (2006). Endocytosis at the blood–brain barrier: from basic understanding to drug delivery strategies. J Drug Target.

[263] Jones AR, Shusta EV (2007). Blood–brain barrier transport of therapeutics via receptor-mediation. Pharm Res.

[264] Begley DJ, Touitou E, Barry BW (2007). Structure and function of the blood–brain barrier. Enhancement in drug delivery.

[265] Zuchero YJY, Chen X, Bien-Ly N, Bumbaca D, Tong RK, Gao X, Zhang S, Hoyte K, Luk W, Huntley MA (2016). Discovery of novel blood–brain barrier targets to enhance brain uptake of therapeutic antibodies. Neuron.

[266] Pardridge WM (2017). Delivery of biologics across the blood–brain barrier with molecular Trojan horse technology. Biodrugs.

[267] Broadwell RD, Baker-Cairns BJ, Friden PM, Oliver C, Villegas JC (1996). Transcytosis of protein through the mammalian cerebral epithelium and endothelium. III. Receptor-mediated transcytosis through the blood–brain barrier of blood-borne transferrin and antibody against the transferrin receptor. Exp Neurol.

[268] Widera A, Norouziyan F, Shen WC (2003). Mechanisms of TfR-mediated transcytosis and sorting in epithelial cells and applications toward drug delivery. Adv Drug Deliv Rev.

[269] Zhang Y, Pardridge WM (2001). Mediated efflux of IgG molecules from brain to blood across the blood–brain barrier. J Neuroimmunol.

[270] Deane R, Sagare A, Hamm K, Parisi M, LaRue B, Guo H, Wu Z, Holtzman DM, Zlokovic BV (2005). IgG-assisted age-dependent clearance of Alzheimer’s amyloid beta peptide by the blood–brain barrier neonatal Fc receptor. J Neurosci.

[271] Garg A, Balthasar JP (2009). Investigation of the influence of FcRn on the distribution of IgG to the brain. AAPS J.

[272] Banks WA (2012). Drug delivery to the brain in Alzheimer’s disease: consideration of the blood–brain barrier. Adv Drug Deliv Rev.

[273] Cooper PR, Ciambrone GJ, Kliwinski CM, Maze E, Johnson L, Li QQ, Feng YQ, Hornby PJ (2013). Efflux of monoclonal antibodies from rat brain by neonatal Fc receptor, FcRn. Brain Res.

[274] Abuqayyas L, Balthasar JP (2013). Investigation of the Role of Fc gamma R and FcRn in mAb distribution to the brain. Mol Pharm.

[275] Finke JM, Ayres KR, Brisbin RP, Hill HA, Wing EE, Banks WA (2017). Antibody blood–brain barrier efflux is modulated by glycan modification. Biochim Biophys Acta Gen Subj.

[276] Fishman JB, Rubin JB, Handrahan JV, Connor JR, Fine RE (1987). Receptor-mediated transcytosis of transferrin across the blood–brain-barrier. J Neurosci Res.

[277] Crowe A, Morgan EH (1992). Iron and transferrin uptake by brain and cerebrospinal fluid in the rat. Brain Res.

[278] Moos T, Morgan EH (1998). Kinetics and distribution of [Fe-59-I-125]transferrin injected into the ventricular system of the rat. Brain Res.

[279] Burdo JR, Antonetti DA, Wolpert EB, Connor JR (2003). Mechanisms and regulation of transferrin and iron transport in a model blood–brain barrier system. Neuroscience.

[280] Simpson IA, Ponnuru P, Klinger ME, Myers RL, Devraj K, Coe CL, Lubach GR, Carruthers A, Connor JR (2015). A novel model for brain iron uptake: introducing the concept of regulation. J Cereb Blood Flow Metab.

[281] Burkhart A, Skjorringe T, Johnsen KB, Siupka P, Thomsen LB, Nielsen MS, Thomsen LL, Moos T (2016). Expression of iron-related proteins at the neurovascular unit supports reduction and reoxidation of iron for transport through the blood–brain barrier. Mol Neurobiol.

[282] Duck KA, Simpson IA, Connor JR (2017). Regulatory mechanisms for iron transport across the blood–brain barrier. Biochem Biophys Res Commun.

[283] Sweet WH, Selverstone B, Soloway S, Stetten D (1950). Studies of formation, flow and absorption of cerebrospinal fluid. II. Studies with heavy water in the normal man. Surg Forum.

[284] Sweet WH, Brownell GL, Scholl JA, Bowsher DR, Benda P, Stickley EE (1954). The formation, flow and absorption of cerebrospinal fluid—newer concepts based on studies with isotopes. Res Publ Assoc Res Nerv Ment Dis.

[285] Bering EA (1952). Water exchange of central nervous system and cerebrospinal fluid. J Neurosurg.

[286] Yudilevich DL, De Rose N (1971). Blood–brain transfer of glucose and other molecules measured by rapid indicator dilution. Am J Physiol.

[287] Eichling JO, Raichle ME, Grubb RL, Ter-Pogossian MM (1974). Evidence of the limitations of water as a freely diffusible tracer in brain of the rhesus monkey. Circ Res.

[288] Gjedde A, Andersson J, Eklof B (1975). Brain uptake of lactate, antipyrine, water and ethanol. Acta Physiol Scand.

[289] Takagi S, Ehara K, Finn RD (1987). Water extraction fraction and permeability-surface product after intravenous injection in rats. Stroke.

[290] Paulson OB, Hertz MM, Bolwig TG, Lassen NA (1977). Filtration and diffusion of water across blood–brain-barrier in man. Microvasc Res.

[291] Fenstermacher JD, Johnson JA (1966). Filtration and reflection coefficients of the rabbit blood–brain barrier. Am J Physiol.

[292] Fenstermacher JD, Staub NC, Taylor AE (1984). Volume regulation of the central nervous system. Edema.

[293] Patlak CS, Paulson OB (1981). The role of unstirred layers for water exchange across the blood–brain barrier. Microvasc Res.

[294] Johnson DC, Hoop B, Kazemi H (1983). Movement of CO_2_ and HCO_3_^−^ from blood to brain in dogs. J Appl Physiol Respir Environ Exerc Physiol.

[295] Sokoloff L (1992). The brain as a chemical machine. Prog Brain Res.

[296] Sokoloff L, Field J, Magoun HW, Hall VE (1960). The metabolism of the central nervous system in vivo. Handbook of physiology section 1 neurophysiology.

[297] Leusen I (1972). Regulation of cerebrospinal-fluid composition with reference to breathing. Physiol Rev.

[298] Mitchell RA, Herbert DA, Carman CT (1965). Acid-base constants and temperature coefficients for cerebrospinal fluid. J Appl Physiol.

[299] Crone C (1965). Facilitated transfer of glucose from blood into brain tissue. J Physiol (Lond).

[300] Oldendorf WH (1971). Brain uptake of radiolabeled amino acids, amines, and hexoses after arterial injection. Am J Physiol.

[301] Cutler RW, Sipe JC (1971). Mediated transport of glucose between blood and brain in the cat. Am J Physiol.

[302] Betz AL, Gilboe DD, Drewes LR (1974). Effects of anoxia on net uptake and unidirectional transport of glucose into the isolated dog brain. Brain Res.

[303] Dick AP, Harik SI, Klip A, Walker DM (1984). Identification and characterization of the glucose transporter of the blood–brain barrier by cytochalasin B binding and immunological reactivity. Proc Natl Acad Sci USA.

[304] Kalaria RN, Harik SI (1989). Reduced glucose transporter at the blood–brain barrier and in cerebral cortex in Alzheimer disease. J Neurochem.

[305] Pardridge WM, Boado RJ, Farrell CR (1990). Brain-type glucose transporter (GLUT-1) is selectively localized to the blood–brain barrier. Studies with quantitative western blotting and in situ hybridization. J Biol Chem.

[306] Ohtsuki S, Ikeda C, Uchida Y, Sakamoto Y, Miller F, Glacial F, Decleves X, Scherrmann J-M, Couraud P-O, Kubo Y (2013). Quantitative targeted absolute proteomic analysis of transporters, receptors and junction proteins for validation of human cerebral microvascular endothelial cell line hCMEC/D3 as a human blood–brain barrier model. Mol Pharm.

[307] Hawkins RA (1986). Transport of essential nutrients across the blood–brain barrier of individual structures. Fed Proc.

[308] Bachelard HS, Daniel PM, Love ER, Pratt OE (1972). The in vivo influx of glucose into the brain of the rat compared with the net cerebral uptake. J Physiol (Lond).

[309] Bachelard HS, Daniel PM, Love ER, Pratt OE (1973). The transport of glucose into the brain of the rat in vivo. Proc R Soc Lond Ser B Biol Sci.

[310] Lund-Andersen H (1979). Transport of glucose from blood to brain. Physiol Rev.

[311] Gjedde A, Diemer NH (1983). Autoradiographic determination of regional brain glucose content. J Cereb Blood Flow Metab.

[312] Holden JE, Mori K, Dienel GA, Cruz NF, Nelson T, Sokoloff L (1991). Modeling the dependence of hexose distribution volumes in brain on plasma glucose concentration: implications for estimation of the local 2-deoxyglucose lumped constant. J Cereb Blood Flow Metab.

[313] Gruetter R, Novotny EJ, Boulware SD, Rothman DL, Shulman RG (1996). ^1^H NMR studies of glucose transport in the human brain. J Cereb Blood Flow Metab.

[314] Barros LF, Bittner CX, Loaiza A, Porras OH (2007). A quantitative overview of glucose dynamics in the gliovascular unit. Glia.

[315] Simpson IA, Carruthers A, Vannucci SJ (2007). Supply and demand in cerebral energy metabolism: the role of nutrient transporters. J Cereb Blood Flow Metab.

[316] Pfeuffer J, Tkac I, Gruetter R (2000). Extracellular-intracellular distribution of glucose and lactate in the rat brain assessed noninvasively by diffusion-weighted ^1^H nuclear magnetic resonance spectroscopy in vivo. J Cereb Blood Flow Metab.

[317] Lowry OH, Passonneau JV (1964). The relationships between substrates and enzymes of glycolysis in brain. J Biol Chem.

[318] Thompson MF, Bachelard HS (1970). Cerebral-cortex hexokinase. Comparison of properties of solubilized mitochondrial and cytoplasmic activities. Biochem J.

[319] Buschiazzo PM, Terrell EB, Regen DM (1970). Sugar transport across the blood–brain barrier. Am J Physiol.

[320] MacAulay N, Zeuthen T (2010). Water transport between CNS compartments: contributions of aquaporins and cotransporters. Neuroscience.

[321] Deng D, Xu C, Sun P, Wu J, Yan C, Hu M, Yan N (2014). Crystal structure of the human glucose transporter GLUT1. Nature.

[322] Cura AJ, Carruthers A (2012). Role of monosaccharide transport proteins in carbohydrate assimilation, distribution, metabolism, and homeostasis. Compr Physiol.

[323] Widdas WF (1952). Inability of diffusion to account for placental glucose transfer in the sheep and consideration of the kinetics of a possible carrier transfer. J Physiol (Lond).

[324] Rosenberg T, Wilbrandt W (1957). Uphill transport induced by counterflow. J Gen Physiol.

[325] Regen DM, Morgan HE (1964). Studies of fhe glucose-transport system in the rabbit erythrocyte. Biochim Biophys Acta.

[326] Lieb WR, Stein WD (1974). Testing and characterizing the simple carrier. Biochim Biophys Acta.

[327] Betz AL, Gilboe DD, Drewes LR (1975). Accelerative exchange diffusion kinetics of glucose between blood and brain and its relation to transport during anoxia. Biochim Biophys Acta.

[328] Carruthers A (1990). Facilitated diffusion of glucose. Physiol Rev.

[329] Carruthers A (1991). Mechanisms for the facilitated diffusion of substrates across cell membranes. Biochemistry.

[330] Yan N (2017). A glimpse of membrane transport through structures-advances in the structural biology of the glut glucose transporters. J Mol Biol.

[331] Gjedde A, Christensen O (1984). Estimates of Michaelis–Menten constants for the two membranes of the brain endothelium. J Cereb Blood Flow Metab.

[332] Cunningham VJ, Hargreaves RJ, Pelling D, Moorhouse SR (1986). Regional blood–brain glucose transfer in the rat: a novel double-membrane kinetic analysis. J Cereb Blood Flow Metab.

[333] Knudsen GM, Pettigrew KD, Paulson OB, Hertz MM, Patlak CS (1990). Kinetic analysis of blood–brain barrier transport of d-glucose in man: quantitative evaluation in the presence of tracer backflux and capillary heterogeneity. Microvasc Res.

[334] Mason GF, Behar KL, Rothman DL, Shulman RG (1992). NMR determination of intracerebral glucose concentration and transport kinetics in rat brain. J Cereb Blood Flow Metab.

[335] Gjedde A, Bradbury MWB (1992). Blood–brain glucose transfer. Physiology and pharmacology of the blood–brain barrier.

[336] Patching SG (2017). Glucose transporters at the blood–brain barrier: function, regulation and gateways for drug delivery. Mol Neurobiol.

[337] Gruetter R, Ugurbil K, Seaquist ER (1998). Steady-state cerebral glucose concentrations and transport in the human brain. J Neurochem.

[338] Choi IY, Lee SP, Kim SG, Gruetter R (2001). In vivo measurements of brain glucose transport using the reversible Michaelis–Menten model and simultaneous measurements of cerebral blood flow changes during hypoglycemia. J Cereb Blood Flow Metab.

[339] Duarte JMN, Morgenthaler FD, Lei H, Poitry-Yamate C, Gruetter R (2009). Steady-state brain glucose transport kinetics re-evaluated with a four-state conformational model. Front Neuroenerget.

[340] Shestov AA, Emir UE, Kumar A, Henry PG, Seaquist ER, Oz G (2011). Simultaneous measurement of glucose transport and utilization in the human brain. Am J Physiol.

[341] Duarte JMN, Gruetter R (2012). Characterization of cerebral glucose dynamics in vivo with a four-state conformational model of transport at the blood–brain barrier. J Neurochem.

[342] Fillenz M (2005). The role of lactate in brain metabolism. Neurochem Int.

[343] Magistretti PJ, Allaman I (2015). A cellular perspective on brain energy metabolism and functional imaging. Neuron.

[344] Petit JM, Magistretti PJ (2016). Regulation of neuron-astrocyte metabolic coupling across the sleep-wake cycle. Neuroscience.

[345] Dienel GA (2017). Lack of appropriate stoichiometry: strong evidence against an energetically important astrocyte-neuron lactate shuttle in brain. J Neurosci Res.

[346] Diaz-Garcia CM, Mongeon R, Lahmann C, Koveal D, Zucker H, Yellen G (2017). Neuronal stimulation triggers neuronal glycolysis and not lactate uptake. Cell Metab.

[347] Sonnewald U (2014). Glutamate synthesis has to be matched by its degradation—where do all the carbons go?. J Neurochem.

[348] Dienel GA, McKenna MC (2014). A dogma-breaking concept: glutamate oxidation in astrocytes is the source of lactate during aerobic glycolysis in resting subjects. J Neurochem.

[349] Daniel PM, Love ER, Moorhouse SR, Pratt OE (1972). The movement of ketone bodies, glucose, pyruvate and lactate between blood and brain of rats. J Physiol (Lond).

[350] Drewes LR, Gilboe DD (1973). Glycolysis and the permeation of glucose and lactate in the isolated, perfused dog brain during anoxia and postanoxic recovery. J Biol Chem.

[351] Knudsen GM, Paulson OB, Hertz MM (1991). Kinetic analysis of the human blood–brain barrier transport of lactate and its influence by hypercapnia. J Cereb Blood Flow Metab.

[352] Boumezbeur F, Petersen KF, Cline GW, Mason GF, Behar KL, Shulman GI, Rothman DL (2010). The contribution of blood lactate to brain energy metabolism in humans measured by dynamic ^13^C nuclear magnetic resonance spectroscopy. J Neurosci.

[353] Quistorff B, Secher NH, Van Lieshout JJ (2008). Lactate fuels the human brain during exercise. FASEB J.

[354] Cruz NF, Adachi K, Dienel GA (1999). Rapid efflux of lactate from cerebral cortex during K^+^-induced spreading cortical depression. J Cereb Blood Flow Metab.

[355] Madsen PL, Cruz NF, Sokoloff L, Dienel GA (1999). Cerebral oxygen/glucose ratio is low during sensory stimulation and rises above normal during recovery: excess glucose consumption during stimulation is not accounted for by lactate efflux from or accumulation in brain tissue. J Cereb Blood Flow Metab.

[356] Dienel GA, Cruz NF (2003). Neighborly interactions of metabolically-activated astrocytes in vivo. Neurochem Int.

[357] Dienel GA, Cruz NF (2004). Nutrition during brain activation: does cell-to-cell lactate shuttling contribute significantly to sweet and sour food for thought?. Neurochem Int.

[358] Gandhi GK, Cruz NF, Ball KK, Dienel GA (2009). Astrocytes are poised for lactate trafficking and release from activated brain and for supply of glucose to neurons. J Neurochem.

[359] Cooper AJL, Plum F (1987). Biochemistry and physiology of brain ammonia. Physiol Rev.

[360] Lee W-J, Hawkins RA, Vina JR, Peterson DR (1998). Glutamine transport by the blood–brain barrier: a possible mechanism for nitrogen removal. Am J Physiol.

[361] Dunlop DS, van Elden W, Lajtha A (1975). A method for measuring brain protein synthesis rates in young and adult rats. J Neurochem.

[362] Dunlop DS (1978). Measuring protein synthesis and degradation rates in CNS tissue. Res Methods Neurochem.

[363] Dunlop DS, Kaufman H, Zanchin G, Lajtha A (1984). Protein synthesis rates in rats with portacaval shunts. J Neurochem.

[364] Buniatian HC, Lajtha A (1971). The urea cycle. Handbook of neurochemistry.

[365] Betz AL, Gilboe DD (1973). Effect of pentobarbital on amino acid and urea flux in the isolated dog brain. Am J Physiol.

[366] Cooper AJ, McDonald JM, Gelbard AS, Gledhill RF, Duffy TE (1979). The metabolic fate of 13N-labeled ammonia in rat brain. J Biol Chem.

[367] Taylor CJ, Nicola PA, Wang S, Barrand MA, Hladky SB (2006). Transporters involved in the regulation of intracellular pH (pH_i_) in primary cultured rat brain endothelial cells. J Physiol (Lond).

[368] Dejong CH, Deutz NE, Soeters PB (1993). Cerebral cortex ammonia and glutamine metabolism in two rat models of chronic liver insufficiency-induced hyperammonemia: influence of pair-feeding. J Neurochem.

[369] Banos G, Daniel PM, Moorhouse SR, Pratt OE (1973). The influx of amino acids into the brain of the rat in vivo: the essential compared with some non-essential amino acids. Proc R Soc Lond Ser B Biol Sci.

[370] Smith QR, Takasato Y (1986). Kinetics of amino acid transport at the blood–brain barrier studied using an in situ brain perfusion technique. Ann NY Acad Sci.

[371] Smith QR, Momma S, Aoyagi M, Rapoport SI (1987). Kinetics of neutral amino acid transport across the blood–brain barrier. J Neurochem.

[372] Pardridge WM, Oldendorf WH (1975). Kinetic analysis of blood–brain barrier transport of amino acids. Biochim Biophys Acta.

[373] Betz AL, Goldstein GW (1978). Polarity of the blood–brain barrier: neutral amino acid transport into isolated brain capillaries. Science.

[374] Hutchison HT, Eisenberg HM, Haber B (1985). High-affinity transport of glutamate in rat brain microvessels. Exp Neurol.

[375] del Pino MMS, Hawkins RA, Peterson DR (1992). Neutral amino acid transport by the blood–brain barrier membrane vesicle studies. J Biol Chem.

[376] Lorenzo AV, Snodgrass SR (1972). Leucine transport from the ventricles and the cranial subarachnoid space in the cat. J Neurochem.

[377] Davson H, Hollingsworth JG, Carey MB, Fenstermacher JD (1982). Ventriculo-cisternal perfusion of twelve amino acids in the rabbit. J Neurobiol.

[378] Brosnan JT, Man KC, Hall DE, Colbourne SA, Brosnan ME (1983). Interorgan metabolism of amino acids in streptozotocin-diabetic ketoacidotic rat. Am J Physiol.

[379] Pardridge WM, Connor JD, Crawford IL (1975). Permeability changes in the blood–brain barrier: causes and consequences. CRC Crit Rev Toxicol.

[380] Yudkoff M, Daikhin Y, Lin ZP, Nissim I, Stern J, Pleasure D, Nissim I (1994). Interrelationships of leucine and glutamate metabolism in cultured astrocytes. J Neurochem.

[381] Yudkoff M, Daikhin Y, Nelson D, Nissim I, Erecinska M (1996). Neuronal metabolism of branched-chain amino acids: flux through the aminotransferase pathway in synaptosomes. J Neurochem.

[382] Hutson SM, Berkich D, Drown P, Xu B, Aschner M, LaNoue KF (1998). Role of branched-chain aminotransferase isoenzymes and gabapentin in neurotransmitter metabolism. J Neurochem.

[383] Sakai R, Cohen DM, Henry JF, Burrin DG, Reeds PJ (2004). Leucine-nitrogen metabolism in the brain of conscious rats: its role as a nitrogen carrier in glutamate synthesis in glial and neuronal metabolic compartments. J Neurochem.

[384] Hutson SM, Lieth E, LaNoue KF (2001). Function of leucine in excitatory neurotransmitter metabolism in the central nervous system. J Nutr.

[385] Rothman DL, De Feyter HM, Maciejewski PK, Behar KL (2012). Is there in vivo evidence for amino acid shuttles carrying ammonia from neurons to astrocytes?. Neurochem Res.

[386] Sperringer JE, Addington A, Hutson SM (2017). Branched-chain amino acids and brain metabolism. Neurochem Res.

[387] O’Kane RL, Martinez-Lopez I, DeJoseph MR, Vina JR, Hawkins RA (1999). Na + -dependent glutamate transporters (EAAT1, EAAT2, and EAAT3) of the blood–brain barrier—A mechanism for glutamate removal. J Biol Chem.

[388] Zerangue N, Kavanaugh MP (1996). Flux coupling in a neuronal glutamate transporter. Nature.

[389] Levy LM, Warr O, Attwell D (1998). Stoichiometry of the glial glutamate transporter GLT-1 expressed inducibly in a Chinese hamster ovary cell line selected for low endogenous Na^+^-dependent glutamate uptake. J Neurosci.

[390] Owe SG, Marcaggi P, Attwell D (2006). The ionic stoichiometry of the GLAST glutamate transporter in salamander retinal glia. J Physiol (Lond).

[391] Helms HCC, Nielsen CU, Brodin B (2014). Glutamate efflux at the blood–brain barrier: cellular mechanisms and potential clinical relevance. Arch Med Res.

[392] Helms HCC, Aldana BI, Groth S, Jensen MM, Waagepetersen HS, Nielsen CU, Brodin B (2017). Characterization of the l-glutamate clearance pathways across the blood–brain barrier and the effect of astrocytes in an in vitro blood–brain barrier model. J Cereb Blood Flow Metab.

[393] Broer S (2008). Amino acid transport across mammalian intestinal and renal epithelia. Physiol Rev.

[394] Abdul-Ghani A-S, Marton M, Dobkin J (1978). Studies on the transport of glutamine in vivo between the brain and blood in the resting state and during afferent electrical stimulation. J Neurochem.

[395] Hosoya K-I, Tomi M, Ohtsuki S, Takanaga H, Saeki S, Kanai Y, Endou H, Naito M, Tsuruo T, Terasaki T (2002). Enhancement of L-cystine transport activity and its relation to xCT gene induction at the blood–brain barrier by diethyl maleate treatment. J Pharmacol Exp Ther.

[396] Benrabh H, Lefauconnier JM (1996). Glutamate is transported across the rat blood–brain barrier by a sodium-independent system. Neurosci Lett.

[397] Fotiadis D, Kanai Y, Palacin M (2013). The SLC3 and SLC7 families of amino acid transporters. Molec Aspects Med.

[398] O’Kane RL, Vina JR, Simpson I, Zaragoza R, Mokashi A, Hawkins RA (2006). Cationic amino acid transport across the blood–brain barrier is mediated exclusively by system y(+). Am J Physiol.

[399] Deves R, Boyd CA (1998). Transporters for cationic amino acids in animal cells: discovery, structure, and function. Physiol Rev.

[400] Rotoli BM, Closs EI, Barilli A, Visigalli R, Simon A, Habermeier A, Bianchi N, Gambari R, Gazzola GC, Bussolati O, Dall’Asta V (2009). Arginine transport in human erythroid cells: discrimination of CAT1 and 4F2hc/y(+)LAT2 roles. Pflügers Arch.

[401] Boado RJ, Li JY, Nagaya M, Zhang C, Pardridge WM (1999). Selective expression of the large neutral amino acid transporter at the blood–brain barrier. Proc Natl Acad Sci USA.

[402] Duelli R, Enerson BE, Gerhart DZ, Drewes LR (2000). Expression of large amino acid transporter LAT1 in rat brain endothelium. J Cereb Blood Flow Metab.

[403] Matsuo H, Tsukada S, Nakata T, Chairoungdua A, Kim DK, Cha SH, Inatomi J, Yorifuji H, Fukuda J, Endou H, Kanai Y (2000). Expression of a system L neutral amino acid transporter at the blood–brain barrier. NeuroReport.

[404] Oxender DL, Christensen HN (1963). Evidence for two types of mediation of neutral and amino-acid transport in Ehrlich cells. Nature.

[405] Oxender DL, Christensen HN (1963). Distinct mediating systems for the transport of neutral amino acids by the Ehrlich cell. J Biol Chem.

[406] Kanai Y, Segawa H, Miyamoto K, Uchino H, Takeda E, Endou H (1998). Expression cloning and characterization of a transporter for large neutral amino acids activated by the heavy chain of 4F2 antigen (CD98). J Biol Chem.

[407] Meier C, Ristic Z, Klauser S, Verrey F (2002). Activation of system L heterodimeric amino acid exchangers by intracellular substrates. EMBO J.

[408] Verrey F (2003). System L: heteromeric exchangers of large, neutral amino acids involved in directional transport. Pflügers Arch.

[409] Taslimifar M, Buoso S, Verrey F, Kurtcuoglu V (2018). Functional polarity of microvascular brain endothelial cells supported by neurovascular unit computational model of large neutral amino acid homeostasis. Front Physiol.

[410] Tarlungeanu DC, Deliu E, Dotter CP, Kara M, Janiesch PC, Scalise M, Galluccio M, Tesulov M, Morelli E, Sonmez FM (2016). Impaired amino acid transport at the blood brain barrier is a cause of autism spectrum disorder. Cell.

[411] Sinclair LV, Rolf J, Emslie E, Shi Y-B, Taylor PM, Cantrell DA (2013). Control of amino-acid transport by antigen receptors coordinates the metabolic reprogramming essential for T cell differentiation. Nat Immunol.

[412] Napolitano L, Scalise M, Galluccio M, Pochini L, Albanese LM, Indiveri C (2015). LAT1 is the transport competent unit of the LAT1/CD98 heterodimeric amino acid transporter. Int J Biochem Cell Biol.

[413] Cangiano C, Cardelli-Cangiano P, James JH, Rossi-Fanelli F, Patrizi MA, Brackett KA, Strom R, Fischer JE (1983). Brain microvessels take up large neutral amino acids in exchange for glutamine. Cooperative role of Na^+^-dependent and Na^+^-independent systems. J Biol Chem.

[414] Cardellicangiano P, Fiori A, Cangiano C, Barberini F, Allegra P, Peresempio V, Strom R (1987). Isolated brain microvessels as invitro equivalents of the blood–brain-barrier—selective removal by collagenase of the A-system of neutral amino-acid-transport. J Neurochem.

[415] Ennis SR, Kawai N, Ren XD, Abdelkarim GE, Keep RF (1998). Glutamine uptake at the blood–brain barrier is mediated by N-system transport. J Neurochem.

[416] Ruderisch N, Virgintino D, Makrides V, Verrey F (2011). Differential axial localization along the mouse brain vascular tree of luminal sodium-dependent glutamine transporters Snat1 and Snat3. J Cereb Blood Flow Metab.

[417] Davson H, Welch K (1971). The permeation of several materials into the fluids of the rabbit’s brain. J Physiol (Lond).

[418] Crone C (1984). Lack of selectivity to small ions in paracellular pathways in cerebral and muscle capillaries of the frog. J Physiol (Lond).

[419] Smith QR, Rapoport SI (1986). Cerebrovascular permeability coefficients to sodium, potassium, and chloride. J Neurochem.

[420] Netter FH (2011). The netter collection of medical illustrations.

[421] Sahar A, Hochwald GM, Ransohoff J (1969). Alternate pathway for cerebrospinal fluid absorption in animals with experimental obstructive hydrocephalus. Exp Neurol.

[422] Mawuenyega KG, Sigurdson W, Ovod V, Munsell L, Kasten T, Morris JC, Yarasheski KE, Bateman RJ (2010). Decreased clearance of CNS beta-amyloid in Alzheimer’s disease. Science.

[423] McInerney MP, Short JL, Nicolazzo JA (2017). Neurovascular alterations in alzheimer’s disease: transporter expression profiles and CNS drug access. AAPS J.

[424] Qosa H, Abuasal BS, Romero IA, Weksler B, Couraud P-O, Keller JN, Kaddoumi A (2014). Differences in amyloid-beta clearance across mouse and human blood–brain barrier models: kinetic analysis and mechanistic modeling. Neuropharmacology.

[425] Pappolla MA, Matsubara E, Vidal R, Pacheco-Quinto J, Poeggeler B, Zagorski M, Sambamurti K (2018). Melatonin treatment enhances abeta lymphatic clearance in a transgenic mouse model of amyloidosis. Curr Alzheimer Res.

[426] Boespflug EL, Iliff JJ (2018). The emerging relationship between interstitial fluid-cerebrospinal fluid exchange, amyloid-beta, and sleep. Biol Psychiatry.

[427] Haass C, Kaether C, Thinakaran G, Sisodia SS (2012). Trafficking and proteolytic processing of APP. Cold Spring Harb Perspect Med.

[428] Cirrito JR, May PC, O’Dell MA, Taylor JW, Parsadanian M, Cramer JW, Audia JE, Nissen JS, Bales KR, Paul SM (2003). In vivo assessment of brain interstitial fluid with microdialysis reveals plaque-associated changes in amyloid-β metabolism and half-life. J Neurosci.

[429] Bell RD, Sagare AP, Friedman AE, Bedi GS, Holtzman DM, Deane R, Zlokovic BV (2007). Transport pathways for clearance of human Alzheimer’s amyloid beta-peptide and apolipoproteins E and J in the mouse central nervous system. J Cereb Blood Flow Metab.

[430] Lomakin A, Chung DS, Benedek GB, Kirschner DA, Teplow DB (1996). On the nucleation and growth of amyloidβ-protein fibrils: detection of nuclei and quantitation of rate constants. Proc Natl Acad Sci USA.

[431] Harper JD, Lansbury PT (1997). Models of amyloid seeding in Alzheimer’s disease and scrapie: mechanistic truths and physiological consequences of the time-dependent solubility of amyloid proteins. Annu Rev Biochem.

[432] Iwata N, Higuchi M, Saido TC (2005). Metabolism of amyloid-beta peptide and Alzheimer’s disease. Pharmacol Ther.

[433] Hortschansky P, Schroeckh V, Christopeit T, Zandomeneghi G, Fandrich M (2005). The aggregation kinetics of Alzheimer’s beta-amyloid peptide is controlled by stochastic nucleation. Protein Sci.

[434] Yan P, Bero AW, Cirrito JR, Xiao Q, Hu X, Wang Y, Gonzales E, Holtzman DM, Lee J-M (2009). Characterizing the appearance and growth of amyloid plaques in APP/PS1 mice. J Neurosci.

[435] Ye L, Fritschi SK, Schelle J, Obermuller U, Degenhardt K, Kaeser SA, Eisele YS, Walker LC, Baumann F, Staufenbiel M, Jucker M (2015). Persistence of Abeta seeds in APP null mouse brain. Nat Neurosci.

[436] Thal DR (2015). Clearance of amyloid beta-protein and its role in the spreading of Alzheimer’s disease pathology. Front Aging Neurosci.

[437] Lambert MP, Barlow AK, Chromy BA, Edwards C, Freed R, Liosatos M, Morgan TE, Rozovsky I, Trommer B, Viola KL (1998). Diffusible, nonfibrillar ligands derived from Abeta1-42 are potent central nervous system neurotoxins. Proc Natl Acad Sci USA.

[438] Zerbinatti CV, Wozniak DF, Cirrito J, Cam JA, Osaka H, Bales KR, Zhuo M, Paul SM, Holtzman DM, Bu GJ (2004). Increased soluble amyloid-beta peptide and memory deficits in amyloid model mice overexpressing the low-density lipoprotein receptor-related protein. Proc Natl Acad Sci USA.

[439] Haass C, Selkoe DJ (2007). Soluble protein oligomers in neurodegeneration: lessons from the Alzheimer’s amyloid beta-peptide. Nat Rev Mol Cell Biol.

[440] Nisbet RM, Polanco J-C, Ittner LM, Gotz J (2015). Tau aggregation and its interplay with amyloid-beta. Acta Neuropathol.

[441] McIntee FL, Giannoni P, Blais S, Sommer G, Neubert TA, Rostagno A, Ghiso J (2016). In vivo differential brain clearance and catabolism of monomeric and oligomeric Alzheimer’s Abeta protein. Front Aging Neurosci.

[442] Lazarov O, Lee M, Peterson DA, Sisodia SS (2002). Evidence that synaptically released beta-amyloid accumulates as extracellular deposits in the hippocampus of transgenic mice. J Neurosci.

[443] Morrone CD, Liu MZ, Black SE, McLaurin J (2015). Interaction between therapeutic interventions for Alzheimer’s disease and physiological Aβ clearance mechanisms. Front Aging Neurosci.

[444] Bateman RJ, Munsell LY, Morris JC, Swarm R, Yarasheski KE, Holtzman DM (2006). Human amyloid-β synthesis and clearance rates as measured in cerebrospinal fluid in vivo. Nat Med.

[445] Iwata N, Tsubuki S, Takaki Y, Watanabe K, Sekiguchi M, Hosoki E, Kawashima-Morishima M, Lee HJ, Hama E, Sekine-Aizawa Y, Saido TC (2000). Identification of the major Abeta1-42-degrading catabolic pathway in brain parenchyma: suppression leads to biochemical and pathological deposition. Nat Med.

[446] Van Uden E, Mallory M, Veinbergs I, Alford M, Rockenstein E, Masliah E (2002). Increased extracellular amyloid deposition and neurodegeneration in human amyloid precursor protein transgenic mice deficient in receptor-associated protein. J Neurosci.

[447] Saido TC, Iwata N (2006). Metabolism of amyloid beta peptide and pathogenesis of Alzheimer’s disease. Towards presymptomatic diagnosis, prevention and therapy. Neurosci Res.

[448] Bu G (2009). Apolipoprotein E and its receptors in Alzheimer’s disease: pathways, pathogenesis and therapy. Nat Rev Neurosci.

[449] Saido T, Leissring MA (2012). Proteolytic degradation of amyloidβ-protein. Cold Spring Harb Perspect Med.

[450] Kanekiyo T, Cirrito JR, Liu C-C, Shinohara M, Li J, Schuler DR, Shinohara M, Holtzman DM, Bu G (2013). Neuronal clearance of amyloid-β by endocytic receptor LRP1. J Neurosci.

[451] Ries M, Sastre M (2016). Mechanisms of Aβ clearance and degradation by glial cells. Front Aging Neurosci.

[452] Zhao Z, Sagare AP, Ma Q, Halliday MR, Kong P, Kisler K, Winkler EA, Ramanathan A, Kanekiyo T, Bu G (2015). Central role for PICALM in amyloid-β blood–brain barrier transcytosis and clearance. Nat Neurosci.

[453] Storck SE, Meister S, Nahrath J, Meissner JN, Schubert N, Di Spiezio A, Baches S, Vandenbroucke RE, Bouter Y, Prikulis I (2016). Endothelial LRP1 transports amyloid-beta(1-42) across the blood–brain barrier. J Clin Invest.

[454] Nelson AR, Sweeney MD, Sagare AP, Zlokovic BV (2016). Neurovascular dysfunction and neurodegeneration in dementia and Alzheimer’s disease. Biochim Biophys Acta.

[455] Peng W, Achariyar TM, Li B, Liao Y, Mestre H, Hitomi E, Regan S, Kasper T, Peng S, Ding F (2016). Suppression of glymphatic fluid transport in a mouse model of Alzheimer’s disease. Neurobiol Dis.

[456] Kanekiyo T, Bu G (2014). The low-density lipoprotein receptor-related protein 1 and amyloid-beta clearance in Alzheimer’s disease. Front Aging Neurosci.

[457] Roberts KF, Elbert DL, Kasten TP, Patterson BW, Sigurdson WC, Connors RE, Ovod V, Munsell LY, Mawuenyega KG, Miller-Thomas MM (2014). Amyloid-beta efflux from the central nervous system into the plasma. Ann Neurol.

[458] Ramanathan A, Nelson AR, Sagare AP, Zlokovic BV (2015). Impaired vascular-mediated clearance of brain amyloid beta in Alzheimer’s disease: the role, regulation and restoration of LRP1. Front Aging Neurosci.

[459] Kanekiyo T, Liu CC, Shinohara M, Li J, Bu GJ (2012). LRP1 in brain vascular smooth muscle cells mediates local clearance of Alzheimer’s amyloid-β. J Neurosci.

[460] Deane R, Wu ZH, Sagare A, Davis J, Yan SD, Hamm K, Xu F, Parisi M, LaRue B, Hu HW (2004). LRP/amyloid β-peptide interaction mediates differential brain efflux of Aβ isoforms. Neuron.

[461] Jaeger LB, Dohgu S, Hwang MC, Farr SA, Murphy MP, Fleegal-DeMotta MA, Lynch JL, Robinson SM, Niehoff ML, Johnson SN (2009). Testing the neurovascular hypothesis of Alzheimer’s disease: lRP-1 antisense reduces blood–brain barrier clearance, increases brain levels of amyloid-beta protein, and impairs cognition. J Alzheimers Dis.

[462] Pflanzner T, Janko MC, Andre-Dohmen B, Reuss S, Weggen S, Roebroek AJM, Kuhlmann CRW, Pietrzik CU (2011). LRP1 mediates bidirectional transcytosis of amyloid-beta across the blood–brain barrier. Neurobiol Aging.

[463] Deane R, Wu ZH, Zlokovic BV (2004). RAGE (Yin) versus LRP (Yang) balance regulates Alzheimer amyloid beta-peptide clearance through transport across the blood–brain barrier. Stroke.

[464] Sagare AP, Bell RD, Zlokovic BV (2013). Neurovascular defects and faulty amyloid-beta vascular clearance in Alzheimer’s disease. J Alzheimers Dis.

[465] Zlokovic BV (2013). Cerebrovascular effects of apolipoprotein E: implications for Alzheimer disease. JAMA Neurol.

[466] Montagne A, Zhao Z, Zlokovic BV (2017). Alzheimer’s disease: a matter of blood–brain barrier dysfunction?. J Exp Med.

[467] Harold D, Abraham R, Hollingworth P, Sims R, Gerrish A, Hamshere ML, Pahwa JS, Moskvina V, Dowzell K, Williams A (2009). Genome-wide association study identifies variants at CLU and PICALM associated with Alzheimer’s disease. Nat Gen.

[468] Lambert J-C, Heath S, Even G, Campion D, Sleegers K, Hiltunen M, Combarros O, Zelenika D, Bullido MJ, Tavernier B (2009). Genome-wide association study identifies variants at CLU and CR1 associated with Alzheimer’s disease. Nat Gen.

[469] Sagare A, Deane R, Bell RD, Johnson B, Hamm K, Pendu R, Marky A, Lenting PJ, Wu Z, Zarcone T (2007). Clearance of amyloid-beta by circulating lipoprotein receptors. Nat Med.

[470] Zlokovic BV, Martel CL, Matsubara E, McComb JG, Zheng G, McCluskey RT, Frangione B, Ghiso J (1996). Glycoprotein 330/megalin: probable role in receptor-mediated transport of apolipoprotein J alone and in a complex with Alzheimer disease amyloid beta at the blood–brain and blood-cerebrospinal fluid barriers. Proc Natl Acad Sci USA.

[471] Shayo M, McLay RN, Kastin AJ, Banks WA (1997). The putative blood–brain barrier transporter for the beta-amyloid binding protein apolipoprotein J is saturated at physiological concentrations. Life Sci.

[472] Calero M, Rostagno A, Matsubara E, Zlokovic B, Frangione B, Ghiso J (2000). Apolipoprotein J (clusterin) and Alzheimer’s disease. Microsc Res Tech.

[473] Lam FC, Liu RH, Lu PH, Shapiro AB, Renoir JM, Sharom FJ, Reiner PB (2001). β-Amyloid efflux mediated by *p*-glycoprotein. J Neurochem.

[474] Vogelgesang S, Warzok RW, Cascorbi I, Kunert-Keil C, Schroeder E, Kroemer HK, Siegmund W, Walker LC, Pahnke J (2004). The role of *P*-glycoprotein in cerebral amyloid angiopathy; implications for the early pathogenesis of Alzheimer’s disease. Curr Alzheimer Res.

[475] Cirrito JR, Deane R, Fagan AM, Spinner ML, Parsadanian M, Finn MB, Jiang H, Prior JL, Sagare A, Bales KR (2005). *P*-glycoprotein deficiency at the blood–brain barrier increases amyloid-beta deposition in an Alzheimer disease mouse model. J Clin Invest.

[476] Vogelgesang S, Kuhnke D, Jedlitschky G, Jucker M, Mosyagin I, Pahnke J, Cascorbi I, Kroemer HK, Walker LC, Warzok RW (2006). *P*-glycoprotein (ABCB1) mediates transport of Alzheimer’s beta-amyloid peptides. Acta Neuropathol.

[477] Kuhnke D, Jedlitschky G, Grube M, Krohn M, Jucker M, Mosyagin I, Cascorbi I, Walker LC, Kroemer HK, Warzok RW, Vogelgesang S (2007). MDR1-*P*-glycoprotein (ABCB1) mediates transport of Alzheimer’s amyloid-β peptides–implications for the mechanisms of Aβ clearance at the blood–brain barrier. Brain Pathol.

[478] Silverberg GD, Messier AA, Miller MC, Machan JT, Majmudar SS, Stopa EG, Donahue JE, Johanson CE (2010). Amyloid efflux transporter expression at the blood–brain barrier declines in normal aging. J Neuropathol Exp Neurol.

[479] Wijesuriya HC, Bullock JY, Faull RLM, Hladky SB, Barrand MA (2010). ABC efflux transporters in brain vasculature of Alzheimer’s subjects. Brain Res.

[480] Hartz AMS, Miller DS, Bauer B (2010). Restoring blood–brain barrier *P*-glycoprotein reduces brain amyloid-beta in a mouse model of Alzheimer’s disease. Mol Pharmacol.

[481] Jeynes B, Provias J (2011). An investigation into the role of *P*-glycoprotein in Alzheimer’s disease lesion pathogenesis. Neurosci Lett.

[482] van Assema DME, Lubberink M, Bauer M, van der Flier WM, Schuit RC, Windhorst AD, Comans EFI, Hoetjes NJ, Tolboom N, Langer O (2012). Blood–brain barrier *P*-glycoprotein function in Alzheimer’s disease. Brain.

[483] Erickson MA, Banks WA (2013). Blood–brain barrier dysfunction as a cause and consequence of Alzheimer’s disease. J Cereb Blood Flow Metab.

[484] Pan WH, Kastin AJ (2014). Can sleep apnea cause Alzheimer’s disease?. Neurosci Biobehav Rev.

[485] Jedlitschky G, Grube M, Mosyagin I, Kroemer HK, Vogelgesang S (2014). Targeting CNS transporters for treatment of neurodegenerative diseases. Curr Pharm Des.

[486] Chiu C, Miller MC, Monahan R, Osgood DP, Stopa EG, Silverberg GD (2015). *P*-glycoprotein expression and amyloid accumulation in human aging and Alzheimer’s disease: preliminary observations. Neurobiol Aging.

[487] van Assema DME, van Berckel BNM (2016). Blood–brain barrier abc-transporter *p*-glycoprotein in Alzheimer’s disease: still a suspect?. Curr Pharm Des.

[488] Bruckmann S, Brenn A, Grube M, Niedrig K, Holtfreter S, Halbach OVU, Groschup M, Keller M, Vogelgesang S (2017). Lack of *P*-glycoprotein results in impairment of removal of beta-amyloid and increased intraparenchymal cerebral amyloid angiopathy after active immunization in a transgenic mouse model of Alzheimer’s disease. Curr Alzheimer Res.

[489] Pereira CD, Martins F, Wiltfang J, Silva O, Rebelo S (2018). ABC transporters are key players in Alzheimer’s disease. J Alzheimers Dis.

[490] Hartz AMS, Zhong Y, Shen AN, Abner EL, Bauer B (2018). Preventing *P*-gp ubiquitination lowers abeta brain levels in an Alzheimer’s disease mouse model. Front Aging Neurosci.

[491] Ito S, Ohtsuki S, Terasaki T (2006). Functional characterization of the brain-to-blood efflux clearance of human amyloid-β peptide (1-40) across the rat blood–brain barrier. Neurosci Res.

[492] Ohtsuki S, Ito S, Terasaki T (2010). Is *P*-glycoprotein involved in amyloid-β elimination across the blood–brain barrier in Alzheimer’s disease?. Clin Pharmacol Ther.

[493] Kohen R, Shofer JB, Korvatska O, Petrie EC, Wang LY, Schellenberg GD, Peskind ER, Wilkinson CW (2011). ABCB1 genotype and CSF beta-amyloid in Alzheimer disease. J Geriatr Psychiatry Neurol.

[494] Bello I, Salerno M (2015). Evidence against a role of *P*-glycoprotein in the clearance of the Alzheimer’s disease A beta(1-42) peptides. Cell Stress Chaperones.

[495] Tai LM, Loughlin AJ, Male DK, Romero IA (2009). *P*-glycoprotein and breast cancer resistance protein restrict apical-to-basolateral permeability of human brain endothelium to amyloid-beta. J Cereb Blood Flow Metab.

[496] Candela P, Gosselet F, Saint-Pol J, Sevin E, Boucau M-C, Boulanger E, Cecchelli R, Fenart L (2010). Apical-to-basolateral transport of amyloid-beta peptides through blood–brain barrier cells is mediated by the receptor for advanced glycation end-products and is restricted by *P*-glycoprotein. J Alzheimers Dis.

[497] Weller RO, Massey A, Newman TA, Hutchings M, Kuo YM, Roher AE (1998). Cerebral amyloid angiopathy: amyloid beta accumulates in putative interstitial fluid drainage pathways in Alzheimer’s disease. Am J Pathol.

[498] Weller RO, Massey A, Kuo YM, Roher AE (2000). Cerebral amyloid angiopathy: accumulation of A beta in interstitial fluid drainage pathways in Alzheimer’s disease. Ann NY Acad Sci.

[499] Yamaguchi H, Yamazaki T, Lemere CA, Frosch MP, Selkoe DJ (1992). Beta amyloid is focally deposited within the outer basement membrane in the amyloid angiopathy of Alzheimer’s disease. An immunoelectron microscopic study. Am J Pathol.

[500] Hawkes CA, Sullivan PM, Hands S, Weller RO, Nicoll JAR, Carare RO (2012). Disruption of arterial perivascular drainage of amyloid-beta from the brains of mice expressing the human APOE epsilon 4 allele. PLoS ONE.

[501] Keable A, Fenna K, Yuen HM, Johnston DA, Smyth NR, Smith C, Salman RA, Samarasekera N, Nicoll JAR, Attems J (2016). Deposition of amyloid beta in the walls of human leptomeningeal arteries in relation to perivascular drainage pathways in cerebral amyloid angiopathy. Biochim Biophys Acta.

[502] Hawkes CA, Jayakody N, Johnston DA, Bechmann I, Carare RO (2014). Failure of perivascular drainage of β-amyloid in cerebral amyloid angiopathy. Brain Pathol.

[503] Morris AWJ, Carare RO, Schreiber S, Hawkes CA (2014). The cerebrovascular basement membrane: role in the clearance of beta-amyloid and cerebral amyloid angiopathy. Front Aging Neurosci.

[504] Sharp MK, Diem AK, Weller RO, Carare RO (2016). Peristalsis with oscillating flow resistance: a mechanism for periarterial clearance of amyloid beta from the brain. Ann Biomed Eng.

[505] Hawkes CA, Hartig W, Kacza J, Schliebs R, Weller RO, Nicoll JA, Carare RO (2011). Perivascular drainage of solutes is impaired in the ageing mouse brain and in the presence of cerebral amyloid angiopathy. Acta Neuropathol.

[506] Zekonyte J, Sakai K, Nicoll JAR, Weller RO, Carare RO (2016). Quantification of molecular interactions between ApoE, amyloid-beta (A beta) and laminin: relevance to accumulation of A beta in Alzheimer’s disease. Biochim Biophys Acta.

[507] Zervas NT, Liszczak TM, Mayberg MR, Black PM (1982). Cerebrospinal fluid may nourish cerebral vessels through pathways in the adventitia that may be analogous to systemic vasa vasorum. J Neurosurg.

[508] Xu W, Xu F, Anderson ME, Kotarba AE, Davis J, Robinson JK, Van Nostrand WE (2014). Cerebral microvascular rather than parenchymal amyloid-beta protein pathology promotes early cognitive impairment in transgenic mice. J Alzheimer’s Dis.

[509] Weller RO, Subash M, Preston SD, Mazanti I, Carare RO (2008). Perivascular drainage of amyloid-beta peptides from the brain and its failure in cerebral amyloid angiopathy and Alzheimer’s disease. Brain Pathol.

[510] Pimentel-Coelho PM, Rivest S (2012). The early contribution of cerebrovascular factors to the pathogenesis of Alzheimer’s disease. Eur J Neurosci.

[511] Cupino TL, Zabel MK (2014). Alzheimer’s silent partner: cerebral amyloid angiopathy. Trans Stroke Res.

[512] Potter R, Patterson BW, Elbert DL, Ovod V, Kasten T, Sigurdson W, Mawuenyega K, Blazey T, Goate A, Chott R (2013). Increased in vivo amyloid-beta42 production, exchange, and loss in presenilin mutation carriers. Sci Transl Med.

[513] Silverberg GD, Miller MC, Messier AA, Majmudar S, Machan JT, Donahue JE, Stopa EG, Johanson CE (2010). Amyloid deposition and influx transporter expression at the blood–brain barrier increase in normal aging. J Neuropathol Exp Neurol.

[514] Kress BT, Iliff JJ, Xia M, Wang M, Wei HS, Zeppenfeld D, Xie L, Kang H, Xu Q, Liew JA (2014). Impairment of paravascular clearance pathways in the aging brain. Ann Neurol.

[515] Attwell D, Buchan AM, Charpak S, Lauritzen M, Macvicar BA, Newman EA (2010). Glial and neuronal control of brain blood flow. Nature.

[516] Hall CN, Reynell C, Gesslein B, Hamilton NB, Mishra A, Sutherland BA, O’Farrell FM, Buchan AM, Lauritzen M, Attwell D (2014). Capillary pericytes regulate cerebral blood flow in health and disease. Nature.

[517] Cruz NF, Ball KK, Dienel GA (2007). Functional imaging of focal brain activation in conscious rats: impact of [^14^C]glucose metabolite spreading and release. J Neurosci Res.

[518] Fox PT, Raichle ME, Mintun MA, Dence C (1988). Nonoxidative glucose consumption during focal physiologic neural activity. Science.

[519] Dalsgaard MK, Quistorff B, Danielsen ER, Selmer C, Vogelsang T, Secher NH (2004). A reduced cerebral metabolic ratio in exercise reflects metabolism and not accumulation of lactate within the human brain. J Physiol (Lond).

[520] Mann GE, Yudilevich DL, Sobrevia L (2003). Regulation of amino acid and glucose transporters in endothelial and smooth muscle cells. Physiol Rev.

[521] Farrell CL, Yang J, Pardridge WM (1992). GLUT-1 glucose transporter is present within apical and basolateral membranes of brain epithelial interfaces and in microvascular endothelia with and without tight junctions. J Histochem Cytochem.

[522] Cura AJ, Carruthers A (2010). Acute modulation of sugar transport in brain capillary endothelial cell cultures during activation of the metabolic stress pathway. J Biol Chem.

[523] Cura AJ, Carruthers A (2012). AMP kinase regulation of sugar transport in brain capillary endothelial cells during acute metabolic stress. Am J Physiol.

[524] Carruthers A, Helgerson AL (1989). The human erythrocyte sugar transporter is also a nucleotide binding protein. Biochemistry.

[525] Cserr HF (1974). Relationship between cerebrospinal fluid and interstitial fluid of brain. Fed Proc.

[526] Iliff JJ, Lee H, Yu M, Feng T, Logan J, Nedergaard M, Benveniste H (2013). Brain-wide pathway for waste clearance captured by contrast-enhanced MRI. J Clin Invest.

[527] Friden M, Bergstrom F, Wan H, Rehngren M, Ahlin G, Hammarlund-Udenaes M, Bredberg U (2011). Measurement of unbound drug exposure in brain: modeling of pH partitioning explains diverging results between the brain slice and brain homogenate methods. Drug Metab Dispos.

[528] Loryan I, Friden M, Hammarlund-Udenaes M (2013). The brain slice method for studying drug distribution in the CNS. Fluids Barriers CNS.

[529] Haydon DA, Hladky SB (1972). Ion transport across thin lipid membranes: a critical discussion of mechanisms in selected systems. Q Rev Biophys.

[530] Hladky SB (1992). Kinetic analysis of lipid soluble ions and carriers. Q Rev Biophys.

[531] Preston E, Haas N (1986). Defining the lower limits of blood–brain barrier permeability: factors affecting the magnitude and interpretation of permeability-area products. J Neurosci Res.

[532] Miah MK, Bickel U, Mehvar R (2017). Effects of hepatic ischemia-reperfusion injury on the blood–brain barrier permeability to [(14)C] and [(13)C]sucrose. Metab Brain Dis.

[533] Thorne RG, Hrabetova S, Nicholson C (2004). Diffusion of epidermal growth factor in rat brain extracellular space measured by integrative optical imaging. J Neurophysiol.

[534] Pardridge WM (1995). Transport of small molecules through the blood–brain-barrier—biology and methodology. Adv Drug Deliv Rev.

[535] Seeman P (1972). The membrane actions of anesthetics and tranquilizers. Pharmacol Rev.

[536] Kamlet MJ, Doherty RM, Abboud JLM, Abraham MH, Taft RW (1986). Solubility—a new look. ChemTech.

[537] Abraham MH, Whiting GS, Doherty RM, Shuely WJ (1990). Hydrogen-bonding. 13. A new method for the characterization of GLC stationary phases—the Laffort data set. J Chem Soc-Perkin Trans 2.

[538] Pardridge WM, Oldendorf WH (1975). Kinetics of blood–brain-barrier transport of hexoses. Biochim Biophys Acta.

[539] Gjedde A (1980). Rapid steady-state analysis of blood–brain glucose transfer in rat. Acta Physiol Scand.

[540] Mahler HR, Cordes EH (1971). Biological chemistry.

[541] Hladky SB (1975). Tests of the carrier model for ion transport by nonactin and trinactin. Biochim Biophys Acta.

[542] Benz R, Stark G (1975). Kinetics of macrotetrolide-induced ion transport across lipid bilayer membranes. Biochim Biophys Acta.

[543] Stark G, Ketterer B, Benz R, Läuger P (1971). The rate constants of valinomycin-mediated ion transport through lipid membranes. Biophys J.

[544] Benz R, Läuger P (1976). Kinetic analysis of carrier-mediated ion transport by the charge-pulse technique. J Membr Biol.

[545] Hladky SB, Leung JCH, Fitzgerald WJ (1995). The mechanism of ion conduction by valinomycin: analysis of charge pulse responses. Biophys J.

[546] Regen DM, Tarpley HL (1974). Anomalous transport kinetics and the glucose carrier hypothesis. Biochim Biophys Acta.

[547] King EL, Altman C (1956). A schematic method of deriving the rate laws for enzyme-catalyzed reactions. J Phys Chem.

[548] Cleland WW (1963). Kinetics of enzyme-catalyzed reactions with 2 or more substrates or products. 1. Nomenclature and rate equations. Biochim Biophys Acta.

[549] Cleland WW (1967). Enzyme Kinetics. Annu Rev Biochem.

[550] Cuppoletti J, Segel IH (1975). Kinetic analysis of active membrane transport systems: equations for net velocity and isotope exchange. J Theor Biol.

[551] Lieb WR, Stein WD (1974). Testing and characterizing the simple pore. Biochim Biophys Acta.

[552] Cunningham VJ (1986). The influence of transport and metabolism on brain glucose content. Ann NY Acad Sci.

[553] Gardner-Medwin AR (1980). Membrane transport and solute migration affecting the brain cell microenvironment. Neurosci Res Program Bull.

[554] Gardner-Medwin AR (1983). A study of the mechanisms by which potassium moves through brain-tissue in the rat. J Physiol (Lond).

[555] Gardner-Medwin AR, Nicholson C (1983). Changes of extracellular potassium activity induced by electric-current through brain-tissue in the rat. J Physiol (Lond).

[556] Gardner-Medwin AR (1983). Analysis of potassium dynamics in mammalian brain-tissue. J Physiol (Lond).

[557] Crone C, Levitt DG, Renkin EM, Michel CC, Geiger SR (1984). Capillary permeability to small solutes. Handbook of physiology section 2 the cardiovascular system volume 4 part 1 microcirculation.

[558] Kakee A, Terasaki T, Sugiyama Y (1997). Selective brain to blood efflux transport of para-aminohippuric acid across the blood–brain barrier: in vivo evidence by use of the brain efflux index method. J Pharmacol Exp Ther.

[559] Kikuchi R, Kusuhara H, Sugiyama D, Sugiyama Y (2003). Contribution of organic anion transporter 3 (Slc22a8) to the elimination of *p*-aminohippuric acid and benzylpenicillin across the blood–brain barrier. J Pharmacol Exp Ther.

[560] Smeets PHE, Van Aubel RAMH, Wouterse AC, Van Den Heuvel JJMW, Russel FGM (2004). Contribution of multidrug resistance protein 2 (MRP2/ABCC2) to the renal excretion of *p*-aminohippurate (PAH) and identification of MRP4 (ABCC4) as a novel PAH transporter. J Am Soc Nephrol.

[561] Kitazawa T, Terasaki T, Suzuki H, Kakee A, Sugiyama Y (1998). Efflux of taurocholic acid across the blood–brain barrier: interaction with cyclic peptides. J Pharmacol Exp Ther.

[562] Hosoya K, Asaba H, Terasaki T (2000). Brain-to-blood efflux transport of estrone-3-sulfate at the blood–brain barrier in rats. Life Sci.

[563] Asaba H, Hosoya K, Takanaga H, Ohtsuki S, Tamura E, Takizawa T, Terasaki T (2000). Blood–brain barrier is involved in the efflux transport of a neuroactive steroid, dehydroepiandrosterone sulfate, via organic anion transporting polypeptide 2. J Neurochem.

[564] Sugiyama D, Kusuhara H, Shitara Y, Abe T, Meier PJ, Sekine T, Endou H, Suzuki H, Sugiyama Y (2001). Characterization of the efflux transport of 17 beta-estradiol-d-17 beta-glucuronide from the brain across the blood–brain barrier. J Pharmacol Exp Ther.

[565] Kikuchi R, Kusuhara H, Abe T, Endou H, Sugiyama Y (2004). Involvement of multiple transporters in the efflux of 3-hydroxy-3-methylglutaryl-CoA reductase inhibitors across the blood–brain barrier. J Pharmacol Exp Ther.

[566] Mori S, Takanaga H, Ohtsuki S, Deguchi T, Kang Y-S, Hosoya K-I, Terasaki T (2003). Rat organic anion transporter 3 (rOAT3) is responsible for brain-to-blood efflux of homovanillic acid at the abluminal membrane of brain capillary endothelial cells. J Cereb Blood Flow Metab.

[567] Ohtsuki S, Asaba H, Takanaga H, Deguchi T, Hosoya KI, Otagiri M, Terasaki T (2002). Role of blood–brain barrier organic anion transporter 3 (OAT3) in the efflux of indoxyl sulfate, a uremic toxin: its involvement in neurotransmitter metabolite clearance from the brain. J Neurochem.

[568] Li L, Agarwal S, Elmquist WF (2013). Brain efflux index to investigate the influence of active efflux on brain distribution of pemetrexed and methotrexate. Drug Metab Dispos.

[569] Suzuki T, Zaima C, Moriki Y, Fukami T, Tomono K (2007). *P*-glycoprotein mediates brain-to-blood efflux transport of buprenorphine across the blood–brain barrier. J Drug Target.

[570] Takasawa K, Terasaki T, Suzuki H, Sugiyama Y (1997). In vivo evidence for carrier-mediated efflux transport of 3′-azido-3′-deoxythymidine and 2′,3′-dideoxyinosine across the blood–brain barrier via a probenecid-sensitive transport system. J Pharmacol Exp Ther.

[571] Kalaria RN, Gravina SA, Schmidley JW, Perry G, Harik SI (1988). The glucose transporter of the human brain and blood–brain barrier. Ann Neurol.

[572] Farrell CL, Pardridge WM (1991). Blood–brain barrier glucose transporter is asymmetrically distributed on brain capillary endothelial lumenal and ablumenal membranes: an electron microscopic immunogold study. Proc Natl Acad Sci USA.

[573] Cornford EM, Hyman S, Swartz BE (1994). The human brain GLUT1 glucose-transporter—ultrastructural localization to the blood–brain-barrier endothelia. J Cereb Blood Flow Metab.

[574] Vannucci SJ, Maher F, Simpson IA (1997). Glucose transporter proteins in brain: delivery of glucose to neurons and glia. Glia.

[575] Simpson IA, Vannucci SJ, DeJoseph MR, Hawkins RA (2001). Glucose transporter asymmetries in the bovine blood–brain barrier. J Biol Chem.

[576] Plum CM (1974). Free amino acid levels in the cerebrospinal fluid of normal humans and their variation in cases of epilepsy and Spielmeyer-Vogt-Batten disease. J Neurochem.

[577] Franklin GM, Dudzinski DS, Cutler RW (1975). Amino acid transport into the cerebrospinal fluid of the rat. J Neurochem.

[578] McGale EH, Pye IF, Stonier C, Hutchinson EC, Aber GM (1977). Studies of the inter-relationship between cerebrospinal fluid and plasma amino acid concentrations in normal individuals. J Neurochem.

[579] Hamberger A, Nystrom B (1984). Extra- and intracellular amino acids in the hippocampus during development of hepatic encephalopathy. Neurochem Res.

[580] Lerma J, Herranz AS, Herreras O, Abraira V, Martin del Rio R (1986). In vivo determination of extracellular concentration of amino acids in the rat hippocampus. A method based on brain dialysis and computerized analysis. Brain Res.

[581] Dolgodilina E, Imobersteg S, Laczko E, Welt T, Verrey F, Makrides V (2016). Brain interstitial fluid glutamine homeostasis is controlled by blood–brain barrier SLC7A5/LAT1 amino acid transporter. J Cereb Blood Flow Metab.

[582] Jacobson I, Sandberg M, Hamberger A (1985). Mass transfer in brain dialysis devices—a new method for the estimation of extracellular amino acids concentration. J Neurosci Methods.

[583] Hawkins RA, Mans AM, Biebuyck JF (1982). Amino acid supply to individual cerebral structures in awake and anesthetized rats. Am J Physiol.

[584] Mans AM, Biebuyck JF, Shelly K, Hawkins RA (1982). Regional blood–brain barrier permeability to amino acids after portacaval anastomosis. J Neurochem.

[585] Pell JM, Bergman EN (1983). Cerebral metabolism of amino acids and glucose in fed and fasted sheep. Am J Physiol.

[586] Felig P, Wahren J, Ahlborg G (1973). Uptake of individual amino acids by the human brain. Proc Soc Exp Biol Med.

[587] Lying-Tunell U, Lindblad BS, Malmlund HO, Persson B (1980). Cerebral blood-flow and metabolic-rate of oxygen, glucose, lactate, pyruvate, ketone-bodies and amino-acids. Acta Neurol Scand.

[588] Eriksson LS, Law DH, Hagenfeldt L, Wahren J (1983). Nitrogen-metabolism of the human-brain. J Neurochem.

[589] Grill V, Bjorkman O, Gutniak M, Lindqvist M (1992). Brain uptake and release of amino acids in nondiabetic and insulin-dependent diabetic subjects: important role of glutamine release for nitrogen balance. Metabolism.

[590] Strauss GI, Knudsen GM, Kondrup J, Moller K, Larsen FS (2001). Cerebral metabolism of ammonia and amino acids in patients with fulminant hepatic failure. Gastroenterology.

[591] Ohno K, Pettigrew KD, Rapoport SI (1978). Lower limits of cerebrovascular permeability to nonelectrolytes in the conscious rat. Am J Physiol.

[592] Amtorp O (1980). Estimation of capillary permeability of inulin, sucrose and mannitol in rat brain cortex. Acta Physiol Scand.

[593] Preston E, Haas N, Allen M (1984). Reduced permeation of 14C-sucrose, ^3^H-mannitol and ^3^H-inulin across blood–brain barrier in nephrectomized rats. Brain Res Bull.

[594] Sisson WB, Oldendorf WH (1971). Brain distribution spaces of mannitol-3H, inulin-14C, and dextran-14C in the rat. Am J Physiol.

[595] Daniel PM, Lam DK, Pratt OE (1985). Comparison of the vascular permeability of the brain and the spinal cord to mannitol and inulin in rats. J Neurochem.

[596] Davson H, Spaziani E (1959). The blood–brain barrier and the extracellular space of brain. J Physiol (Lond).

[597] Reed DJ, Woodbury DM (1963). Kinetics of movement of iodide, sucrose, inulin and radio-iodinated serum albumin in the central nervous system and cerebrospinal fluid of the rat. J Physiol (Lond).

[598] Cameron IR, Davson H, Segal MB (1969). The effect of hypercapnia on the blood–brain barrier to sucrose in the rabbit. Yale J Biol Med.

[599] Smith QR, Ziylan YZ, Robinson PJ, Rapoport SI (1988). Kinetics and distribution volumes for tracers of different sizes in the brain plasma space. Brain Res.

[600] Preston E, Webster J (2002). Differential passage of [14C]sucrose and [3H]inulin across rat blood–brain barrier after cerebral ischemia. Acta Neuropathol.

[601] Kakee A, Tersaki T, Sugiyama Y (1996). Brain efflux index as a novel method of analyzing efflux transport at the blood–brain barrier. J Pharmacol Exp Ther.

[602] Enzmann DR, Pelc NJ (1993). Cerebrospinal fluid flow measured by phase-contrast cine MR. AJNR Am J Neuroradiol..

[603] Levin VA, Fenstermacher JD, Patlak CS (1970). Sucrose and inulin space measurements of cerebral cortex in four mammalian species. Am J Physiol.

[604] Patlak CS, Fenstermacher JD (1975). Measurements of dog blood–brain transfer constants by ventriculocisternal perfusion. Am J Physiol.

[605] Blasberg RG, Patlak C, Fenstermacher JD (1975). Intrathecal chemotherapy: brain tissue profiles after ventriculocisternal perfusion. J Pharmacol Exp Ther.

[606] Reulen HJ, Czernicki Z (2010). Bulk flow and diffusion revisited, and clinical applications. Brain edema XIV.

[607] Bedussi B, van Lier MGJTB, Bartstra JW, de Vos J, Siebes M, VanBavel E, Bakker ENTP (2015). Clearance from the mouse brain by convection of interstitial fluid towards the ventricular system. Fluids Barriers CNS.

[608] Plog BA, Dashnaw ML, Hitomi E, Peng W, Liao Y, Lou N, Deane R, Nedergaard M (2015). Biomarkers of traumatic injury are transported from brain to blood via the glymphatic system. J Neurosci.

[609] Guyton AC, Granger HJ, Taylor AE (1971). Interstitial fluid pressure. Physiol Rev.

[610] Day TD (1952). The permeability of interstitial connective tissue and the nature of the interfibrillary substance. J Physiol (Lond).

[611] Preston BN, Davies M, Ogston AG (1965). The composition and physicochemical properties of hyaluronic acids prepared from ox synovial fluid and from a case of mesothelioma. Biochem J.

[612] Thorne RG, Lakkaraju A, Rodriguez-Boulan E, Nicholson C (2008). In vivo diffusion of lactoferrin in brain extracellular space is regulated by interactions with heparan sulfate. Proc Natl Acad Sci USA.

[613] Benveniste H, Lee H, Ding F, Sun Q, Al-Bizri E, Makaryus R, Probst S, Nedergaard M, Stein EA, Lu H (2017). Anesthesia with dexmedetomidine and low-dose isoflurane increases solute transport via the glymphatic pathway in rat brain when compared with high-dose isoflurane. Anesthesiology.

[614] Liberman YA, Topaly VP (1969). Permeability of bimolecular phospholipid membranes for fat soluble ions. Itogi nauki tekniki Seriia Biofizika.

[615] Le Blanc OHJ (1970). Single ion conductances in lipid bilayers. Biophys Soc Annu Meet Abstr.

[616] Haydon DA, Myers VB (1973). Surface charge, surface dipoles and membrane conductance. Biochim Biophys Acta.

[617] Hladky SB, Haydon DA (1973). Membrane conductance and surface potential. Biochim Biophys Acta.

[618] Pickar AD, Benz R (1978). Transport of oppositely charged lipophilic probe ions in lipid bilayer membranes having various structures. J Membr Biol.

[619] Hladky SB (1979). The carrier mechanism. Curr Topics Membr Transport.

[620] Sweet DH, Chan LMS, Walden R, Yang X-P, Miller DS, Pritchard JB (2003). Organic anion transporter 3 (Slc22a8) is a dicarboxylate exchanger indirectly coupled to the Na^+^ gradient. Am J Physiol.

[621] Li L, Meier PJ, Ballatori N (2000). Oatp2 mediates bidirectional organic solute transport: a role for intracellular glutathione. Mol Pharmacol.

[622] Satlin LM, Amin V, Wolkoff AW (1997). Organic anion transporting polypeptide mediates organic anion/HCO3^−^ exchange. J Biol Chem.

[623] Leuthold S, Hagenbuch B, Mohebbi N, Wagner CA, Meier PJ, Stieger B (2009). Mechanisms of pH-gradient driven transport mediated by organic anion polypeptide transporters. Am J Physiol.

[624] Huwyler J, Pardridge WM (1998). Examination of blood–brain barrier transferrin receptor by confocal fluorescent microscopy of unfixed isolated rat brain capillaries. J Neurochem.

[625] Yu YJ, Zhang Y, Kenrick M, Hoyte K, Luk W, Lu YM, Atwal J, Elliott JM, Prabhu S, Watts RJ, Dennis MS (2011). Boosting brain uptake of a therapeutic antibody by reducing its affinity for a transcytosis target. Sci Transl Med.

[626] Roberts RL, Fine RE, Sandra A (1993). Receptor-mediated endocytosis of transferrin at the blood–brain barrier. J Cell Sci.

[627] Moos T, Morgan EH (2001). Restricted transport of anti-transferrin receptor antibody (OX26) through the blood–brain barrier in the rat. J Neurochem.

[628] Moos T, Nielsen TR, Skjorringe T, Morgan EH (2007). Iron trafficking inside the brain. J Neurochem.

[629] Lundgaard I, Li BM, Xie LL, Kang HY, Sanggaard S, Haswell JDR, Sun W, Goldman S, Blekot S, Nielsen M (2015). Direct neuronal glucose uptake heralds activity-dependent increases in cerebral metabolism. Nat Commun.

[630] Lundgaard I, Lu ML, Yang E, Peng W, Mestre H, Hitomi E, Deane R, Nedergaard M (2016). Glymphatic clearance controls state-dependent changes in brain lactate concentration. J Cereb Blood Flow Metab.

[631] Fellows LK, Boutelle MG, Fillenz M (1992). Extracellular brain glucose levels reflect local neuronal activity: a microdialysis study in awake, freely moving rats. J Neurochem.

[632] Fray AE, Boutelle M, Fillenz M (1997). Extracellular glucose turnover in the striatum of unanaesthetized rats measured by quantitative microdialysis. J Physiol (Lond).

[633] McNay EC, Gold PE (1999). Extracellular glucose concentrations in the rat hippocampus measured by zero-net-flux: effects of microdialysis flow rate, strain, and age. J Neurochem.

[634] Abi-Saab WM, Maggs DG, Jones T, Jacob R, Srihari V, Thompson J, Kerr D, Leone P, Krystal JH, Spencer DD (2002). Striking differences in glucose and lactate levels between brain extracellular fluid and plasma in conscious human subjects: effects of hyperglycemia and hypoglycemia. J Cereb Blood Flow Metab.

[635] McNay EC, Sherwin RS (2004). From artificial cerebro-spinal fluid (aCSF) to artificial extracellular fluid (aECF): microdialysis perfusate composition effects on in vivo brain ECF glucose measurements. J Neurosci Methods.

[636] de Vries MG, Arseneau LM, Lawson ME, Beverly JL (2003). Extracellular glucose in rat ventromedial hypothalamus during acute and recurrent hypoglycemia. Diabetes.

[637] Bongiovanni R, Mchaourab AS, McClellan F, Elsworth J, Double M, Jaskiw GE (2016). Large neutral amino acids levels in primate cerebrospinal fluid do not confirm competitive transport under baseline conditions. Brain Res.

[638] Chikhale EG, Ng KY, Burton PS, Borchardt RT (1994). Hydrogen bonding potential as a determinant of the in vitro and in situ blood–brain barrier permeability of peptides. Pharm Res.

[639] Thorne RG, Hammarlund-Udenaes M (2014). Primer on central nervous system structure/function and vasculature, ventricular system, and fluids of the brain. Drug delivery to the brain.

[640] Motulsky H, Christopoulos A (2004). Fitting models to biological data using linear and nonlinear regression.

[641] Cooper AJL, Jeitner TM (2016). Central role of glutamate metabolism in the maintenance of nitrogen homeostasis in normal and hyperammonemic brain. Biomolecules.

[642] Martinez-Hernandez A, Bell KP, Norenberg MD (1977). Glutamine synthetase: glial localization in brain. Science.

[643] Shank RP, Campbell GL, Lajtha A (1983). Glutamate. Handbook of neurochemistry.

[644] Sibson NR, Dhankhar A, Mason GF, Rothman DL, Behar KL, Shulman RG (1998). Stoichiometric coupling of brain glucose metabolism and glutamatergic neuronal activity. Proc Natl Acad Sci USA.

[645] Yudkoff M (2017). Interactions in the metabolism of glutamate and the branched-chain amino acids and ketoacids in the CNS. Neurochem Res.

[646] Frigerio F, Casimir M, Carobbio S, Maechler P (2008). Tissue specificity of mitochondrial glutamate pathways and the control of metabolic homeostasis. Biochim Biophys Acta.

[647] Spanaki C, Kotzamani D, Petraki Z, Drakos E, Plaitakis A (2014). Heterogeneous cellular distribution of glutamate dehydrogenase in brain and in non-neural tissues. Neurochem Res.

[648] Rothman DL (2001). Studies of metabolic compartmentation and glucose transport using in vivo MRS. NMR Biomed.

[649] Hyder F, Patel AB, Gjedde A, Rothman D, Behar KL, Shulman RG (2006). Neuronal-glial glucose oxidation and glutamatergic-GABAergic function. J Cereb Blood Flow Metab.

[650] Yudkoff M (1997). Brain metabolism of branched-chain amino acids. Glia.

[651] Lebon V, Petersen KF, Cline GW, Shen J, Mason GF, Dufour S, Behar KL, Shulman GI, Rothman DL (2002). Astroglial contribution to brain energy metabolism in humans revealed by ^13^C nuclear magnetic resonance spectroscopy: elucidation of the dominant pathway for neurotransmitter glutamate repletion and measurement of astrocytic oxidative metabolism. J Neurosci.

[652] Mason GF, Petersen KF, de Graaf RA, Shulman GI, Rothman DL (2007). Measurements of the anaplerotic rate in the human cerebral cortex using ^13^C magnetic resonance spectroscopy and [1-13C] and [2-13C] glucose. J Neurochem.

[653] Rothman DL, Behar KL, Hyder F, Shulman RG (2003). In vivo NMR studies of the glutamate neurotransmitter flux and neuroenergetics: implications for brain function. Annu Rev Physiol.

[654] Rothman DL, De Feyter HM, de Graaf RA, Mason GF, Behar KL (2011). ^13^C MRS studies of neuroenergetics and neurotransmitter cycling in humans. NMR Biomed.

[655] Hull J, Hindy ME, Kehoe PG, Chalmers K, Love S, Conway ME (2012). Distribution of the branched chain aminotransferase proteins in the human brain and their role in glutamate regulation. J Neurochem.

[656] Conway ME, Hutson SM (2016). BCAA metabolism and NH3 homeostasis. Adv Neurobiol.

[657] O’Kane RL, Vina JR, Simpson I, Hawkins RA (2004). Na^+^-dependent neutral amino acid transporters A, ASC, and N of the blood–brain barrier: mechanisms for neutral amino acid removal. Am J Physiol.

[658] Zhao Z, Nelson AR, Betsholtz C, Zlokovic BV (2015). Establishment and dysfunction of the blood–brain barrier. Cell.

[659] Dienel GA. Brain glucose metabolism: Integration of energetics with function. Physiol Rev **(in press)**. 10.1152/physrev.00062.2017 30565508

